# The NLRP3–CASP1 Axis Contributes to Pyroptosis in Bovine Mammary Epithelial Cells During Clinical Mastitis

**DOI:** 10.3390/antiox15030385

**Published:** 2026-03-19

**Authors:** Bohao Zhang, Zhen Yang, Yumeng Gao, Na Chen, Weitao Dong, Yong Zhang, Xingxu Zhao, Quanwei Zhang

**Affiliations:** 1 College of Life Science and Technology, Gansu Agriculture University, Lanzhou 730070, China; zhangbh@st.gsau.edu.cn (B.Z.); 1073324020324@st.gsau.edu.cn (Z.Y.); 1073324020322@st.gsau.edu.cn (Y.G.); 1073323020311@st.gsau.edu.cn (N.C.); zhychy@163.com (Y.Z.); 2 College of Veterinary Medicine, Gansu Agriculture University, Lanzhou 730070, China; dongwt@gsau.edu.cn; 3 Gansu Key Laboratory of Animal Generational Physiology and Reproductive Regulation, Lanzhou 730070, China

**Keywords:** clinical mastitis, inflammation, pyroptosis, NLRP3 inflammasome, caspase-1, mammary epithelial cells

## Abstract

Pyroptosis is a pro-inflammatory form of programmed cell death mediated by inflammasomes and caspases and has been implicated in various inflammatory diseases. However, its function and regulatory role in dairy cows with clinical mastitis (CM) remain poorly understood. This study was conducted to investigate the differentially expressed proteins (DEPs) involved in biological processes (BPs) and the Kyoto Encyclopedia of Genes and Genomes pathways related to inflammasome-mediated pyroptosis based on proteomic data and to further explore their potential involvement in mastitis using in vivo and in vitro models. Histopathological analysis revealed morphological features consistent with pyroptosis in the mammary glands of CM-affected cows, including mammary epithelial cell (MEC) membrane disruption, increased reactive oxygen species production, elevated TUNEL–gasdermin D (GSDMD)-positive staining, and inflammatory cell infiltration. Proteomic profiling identified 276 DEPs and 17 BPs, among which NOD-like receptor family pyrin domain-containing 3 (NLRP3) was identified as a key candidate associated with cytokine production, immune defense, and inflammatory responses. Pathway enrichment analysis indicated that NLRP3, caspase-1 (CASP1), and GSDMD were enriched in the NOD-like receptor signaling pathway and were closely associated with mastitis. Immunohistochemical and molecular analyses demonstrated cytoplasmic localization and significant upregulation of NLRP3, CASP1, and GSDMD at both the mRNA and protein levels in CM-affected tissues. In both in vitro and in vivo models, a dose-dependent increase in the expression of pyroptosis-related targets and pro-inflammatory cytokines was observed with the progression of inflammation. Moreover, the pharmacological inhibition of CASP1 attenuated pyroptosis-associated changes and inflammatory responses in BMECs. Collectively, these findings suggest that the NLRP3–CASP1 axis is associated with inflammation-related pyroptosis in bovine mastitis and may represent a potential therapeutic target for clinical mastitis.

## 1. Introduction

Bovine mastitis is a localized inflammatory disorder of the mammary gland (MG) triggered by physical, chemical, or microbial insults and remains one of the most prevalent and economically burdensome diseases in the global dairy industry [1]. Clinical mastitis (CM) is a well-recognized subtype of bovine mastitis, characterized by visible clinical signs including elevated body temperature, mammary gland swelling and redness, induration of mammary tissue, reduced milk yield, and abnormal milk appearance such as watery secretion, clots, flakes, or even bloody milk in severe cases [1,2]. Beyond impairing milk production and quality, CM compromises animal welfare and dairy safety and may lead to irreversible mammary tissue damage, resulting in substantial economic losses worldwide [1,3]. The disease is primarily initiated by pathogenic bacteria, including staphylococci (e.g., *Staphylococcus aureus*), coliforms (e.g., *Escherichia coli*), and streptococci (e.g., *Streptococcus agalactiae*), that invade the MG through the teat canal and provoke the excessive activation of the innate immune response [1]. This inflammatory cascade is frequently accompanied by oxidative stress, which exacerbates epithelial injury and disrupts mammary tissue homeostasis [4,5]. Although antibiotic therapy remains the mainstay of treatment, its limited effectiveness in preventing recurrence and the increasing risk of antimicrobial resistance underscore the urgent need for mechanism-based therapeutic strategies.

Pyroptosis is an inflammatory type of programmed cell death driven by caspase activation and gasdermin D (GSDMD)-dependent membrane pore formation, ultimately resulting in the secretion of pro-inflammatory cytokines such as interleukin (IL)-1β and IL-18 [6,7,8]. Increasing evidence suggests that pyroptosis can be induced by inflammatory signals and cellular damage associated with oxidative stress and serves as a potent amplifier of inflammatory tissue injury [9,10]. Consistently, dysregulated pyroptosis has been implicated in a wide spectrum of inflammatory diseases including sepsis, atherosclerosis, and intestinal inflammation [7,11]. However, the contribution of pyroptosis to bovine mastitis, particularly within mammary epithelial cells (MECs), remains insufficiently characterized.

Among the pyroptosis-related pathways, the NOD-like receptor family pyrin domain-containing 3 (NLRP3) inflammasome is the most extensively studied and is tightly regulated by redox signaling [7,9]. In inflammatory settings, NLRP3 functions as a molecular sensor of pathogen-associated molecular patterns (PAMPs), damage-associated molecular patterns (DAMPs), and oxidative stress-related signals, particularly mitochondrial reactive oxygen species (ROS), thereby contributing to inflammatory responses and tissue injury [7,12,13]. Upon activation, NLRP3 recruits and activates caspase-1 (CASP1), which cleaves pro-IL-1β, pro-IL-18, and GSDMD, ultimately inducing pyroptotic cell death [9,11]. The NLRP3–CASP1–GSDMD axis therefore constitutes a central molecular hub integrating oxidative stress, innate immune activation, and inflammatory tissue injury [14]. In bovine mastitis, activation of the NLRP3 inflammasome in MECs is thought to contribute to excessive inflammation and epithelial damage. In addition to the canonical NLRP3–CASP1 pathway, lipopolysaccharide (LPS) derived from Gram-negative bacteria can activate the non-canonical pyroptotic pathway via caspase-4 (CASP4), directly cleaving GSDMD and inducing rapid inflammatory cell death [8,15]. Moreover, LPS-induced activation of nuclear factor kappa-light-chain-enhancer of activated B cells (NF-κB) and mitogen-activated protein kinase (MAPK) signaling enhances ROS production and primes NLRP3 inflammasome activation, thereby amplifying mammary inflammation [16,17]. Nevertheless, the expression landscape, regulatory interactions, and functional relevance of pyroptosis-associated molecules in bovine mammary tissue remain largely undefined, particularly at the proteomic level.

Therefore, this study employed data-independent acquisition (DIA) proteomics integrated with bioinformatics analyses to comprehensively identify critical pyroptosis-associated differentially expressed proteins (DEPs) in MG tissues from healthy and CM-affected cows. Histological validation was performed in bovine MG tissues, and complementary in vivo and in vitro experiments—including mouse mastitis models and bovine mammary epithelial cells (BMECs)—were conducted to define the expression patterns, functional roles, and regulatory mechanisms of critical pyroptotic targets during mastitis progression. Collectively, this study provides mechanistic insights into the involvement of pyroptotic signaling in cow mastitis and highlights potential molecular targets for early diagnosis and precision intervention.

## 2. Materials and Methods

### 2.1. Sample Preparation and Collection

Lactating Holstein cows of the same age (6 years) and parity (2–3) and similar physical condition were obtained from a large-scale dairy operation located in Wuzhong City, Ningxia Hui Autonomous Region, China. All cows were maintained under uniform management conditions and were fed a total mixed ration (TMR) designed for Holstein cows (Table S1). The health status of their MGs was assessed following standard veterinary protocols. Cows showing clinical signs of mammary gland swelling and redness, induration of mammary tissue, reduced milk yield, and abnormal milk appearance (e.g., watery secretion, clots, flakes, or bloody milk) were preliminarily diagnosed with clinical mastitis. Additionally, milk samples were collected for the somatic cell count (SCC), differential somatic cell count (DSCC) analysis, and California mastitis test (CMT), as well as pathogen isolation and identification, as previously described [18,19,20]. Subsequently, healthy cows (Con group) and clinical mastitis-affected cows (CM group) were classified based on diagnostic results (Supplementary Tables S2 and Figure S1). Each cow was considered one independent biological replicate (*n* = 3 per group). Specifically, cows without typical clinical signs, SCC ≤ 1 × 10^5^ cells/mL, DSCC ≤ 65%, and negative CMT were considered healthy cows. Conversely, cows exhibiting typical mastitis symptoms, SCC ≥ 1.3 × 10^6^ cells/mL, DSCC > 65%, and positive CMT were defined as clinical mastitis cows. MG tissues were obtained immediately following slaughter and either rapidly frozen in liquid nitrogen or chemically preserved with 4% paraformaldehyde or 2.5% glutaraldehyde for subsequent analyses. For all subsequent molecular and histological analyses, each biological sample was analyzed with at least three independent technical replicates unless otherwise specified. All sample collection procedures were conducted in compliance with the institutional animal care regulations authorized by the Animal Care Commission of Gansu Agricultural University (GSAU-Eth-LST-2021-003).

### 2.2. Hematoxylin–Eosin (H&E) Staining and Scanning Electron Microscopy (SEM)

Fixed MGs were processed for paraffin embedding and cut into sections with a microtome (Leica, Wetzlar, Germany). These tissue sections were subjected to dewaxing and stepwise rehydration before H&E staining [18]. Images were obtained with an upright optical microscope (Nikon, Tokyo, Japan). Pyroptotic morphological features were further examined using SEM technology [21]. Specimens were further fixed with 1% osmium tetroxide, then passed through a graded ethanol dehydration series followed by isoamyl acetate treatment, mounted on stubs, sputter-coated with gold, and examined using an SEM (HITACHI, Tokyo, Japan) at 12 kV.

### 2.3. TUNEL–GSDMD, Immunofluorescence (IF), and ROS Staining

Pyroptotic cells were detected using a TUNEL assay kit (Servicebio, Wuhan, China) [22,23]. Samples were subsequently co-stained with anti-GSDMD antibodies. IF staining was performed as described previously [18,24]. Briefly, the samples were exposed to primary antibodies (Table S3) at appropriate dilution and labeled with Alexa Fluor^®^ secondary antibodies [24]. For ROS staining, MG tissue sections and cells seeded in 12-well plates were pretreated as previously described [22]. ROS staining was then detected using dihydroethidium fluorescence probes (Servicebio, Wuhan, China). Cell nuclei were visualized by staining with DAPI (Solarbio, Beijing, China). Fluorescence images were subsequently obtained with a fluorescence imaging platform (Olympus, Tokyo, Japan).

### 2.4. Enzyme-Linked Immunosorbent Assay (ELISA) Analysis

The levels of IL-1β, IL-18, toll-like receptor 4 (TLR4), tumor necrosis factor-alpha (TNF-α), and IL-6 in the serum or cell culture supernatants were quantified using commercial ELISA kits (Jingmei biotechnology, Yancheng, China) [25]. ELISA measurements were performed using three independent biological replicates, and each sample was assayed in triplicate technical replicates.

### 2.5. DIA Proteomic Sequencing

DIA-based proteomic analysis was performed using the same biological samples obtained from the experimental animals described above. Peptides were separated using an UltiMate 3000 nanoLC system (Thermo Fisher Scientific, Waltham, MA, USA) coupled to a timsTOF Pro2 mass spectrometer (Bruker Daltonics, Bremen, Germany). Peptide samples (200 ng) were loaded onto a C18 analytical column (15 cm × 75 μm, 1.7 μm particle size, 120 Å pore size; IonOpticks, Melbourne, Australia) and separated with a 60 min gradient at a flow rate of 400 nL/min and a column temperature of 50 °C. The mobile phase consisted of solvent A (0.1% formic acid in water) and solvent B (80% acetonitrile containing 0.1% formic acid). The gradient increased from 4% to 28% B over 25 min, followed by 44% over 10 min and 90% over 10 min, then re-equilibrated to 4%. Mass spectrometric data were acquired using the diaPASEF mode with 22 precursor isolation windows (40 Th each) covering the *m*/*z* range of 349–1229. The acquisition scheme consisted of a 13-scan diaPASEF cycle with variable repetition steps (2–5). Collision energy was dynamically adjusted according to ion mobility, ranging from 59 eV at 1/K0 = 1.6 Vs/cm^2^ to 20 eV at 1/K0 = 0.6 Vs/cm^2^.

### 2.6. Bioinformatics Analysis

Raw data were processed using Spectronaut X (Biognosys AG, Schlieren, Switzerland) with the Pulsar search engine against the *Bos taurus* reference database (NCBI_GCF_002263795.1). Peptide- and protein-level identifications were filtered at a false discovery rate (*FDR*) of ≤1%, and protein quantification was based on the average intensity of the top three peptides passing the 1% *FDR* threshold. Protein abundance values were normalized using a local normalization algorithm, and missing data were processed by removing proteins absent in more than 50% of samples followed by missing value imputation (MVI). DEPs were defined as those with |log_2_ fold change (FC)| > 0.58 (equivalent to FC > 1.5) and *Q* < 0.05. The raw dataset has been deposited in the ProteomeXchange database (accession number: IPX0013254001/PXD068044). Processed data were subjected to Gene Ontology (GO) annotation and Kyoto Encyclopedia of Genes and Genomes (KEGG) pathway enrichment analysis for the screening of potential target DEPs (*p* < 0.05 and *Q* < 0.05). In this analysis, biological processes (BPs) of GO terms related to cytokine production, immune defense, and inflammatory responses were further examined. After overlapping and intersecting DEPs among these BPs, pathways containing NLRP3 and pyroptosis-associated DEPs were selected for further analysis. Data visualization was performed using R packages and the OmicShare web-based analysis interface [26]. Protein–protein interaction (PPI) analysis was performed with the software STRING v11.5 and Cytoscape 3.9.1 with the ClueGO plugin [27]. The signal transduction diagram was predicted and constructed using Adobe Illustrator 2024 (Adobe Systems, San Jose, CA, USA).

### 2.7. Immunohistochemistry (IHC) Staining

Paraffin-embedded MG tissue sections underwent antigen retrieval followed by blocking treatment according to previously described protocols [18,24]. Subsequently, the sections were exposed to the respective primary antibodies. IHC staining was performed using the Streptavidin–Biotin Complex and the 3,3′-diaminobenzidine kit (Servicebio, Wuhan, China) [18,24]. For negative controls, primary antibodies were replaced with PBS. Images were acquired using a light microscopy system (Nikon, Tokyo, Japan). Each experiment was independently repeated three times.

### 2.8. RNA Extraction, cDNA Synthesis, and qPCR Assays

Total RNA was extracted from tissues and cell samples as described previously [18,24]. After evaluating RNA concentration and integrity, 1 μg of total RNA was converted into generate cDNA using a reverse transcription kit (Accurate Biology, Changsha, China). The mRNA levels were quantified using a LightCycler 96 real-time PCR system (Roche, Basel, Switzerland). qPCR primers (Table S4) were designed using the software Premier v5.0 and synthesized by Qingke Biotech (Yangling, China). β-Actin served as the internal reference gene. All experiments were performed in triplicate. Data were analyzed using the 2^−ΔΔCT^ method [28].

### 2.9. Western Blot (WB)

Total protein was extracted and quantified using RIPA buffer and a bicinchoninic acid protein assay kit (Servicebio, Wuhan, China). Subsequently, WB was conducted to evaluate the expression levels of proteins following previously reported procedures [18]. β-Actin served as the internal reference for normalization. All bands were visualized using a Chemiluminescence Imaging System (Servicebio, Wuhan, China). Exposure times were optimized to avoid saturation of the major signals, and integrated optical density (IOD) values were quantified with the software Image-Pro Plus 6.0 (Media Cybernetics, Rockville, MD, USA). All blot assays were performed in triplicate.

### 2.10. Cell Culture and Treatment

BMECs (ATCC, Beijing, China) were maintained in DMEM containing 10% fetal bovine serum (Gibco, Grand Island, NY, USA) at 37 °C in a humidified incubator supplied with 5% CO_2_. Before stimulation, cells were seeded into culture plates and allowed to grow until the monolayer reached approximately 60–70% confluence. Cells were then incubated in phenol red-free DMEM under serum-deprived conditions for 12 h. Subsequently, BMECs were treated with LPS (Solarbio, Beijing, China) and/or VX765 (Selleck, Shanghai, China) for 24 h. All treatments were conducted in triplicate.

### 2.11. Cell Viability Assays

Cell viability was assessed using a cell counting kit 8 (CCK8; NCM Biotech, Suzhou, China) [18]. BMECs were seeded into 96-well plates and exposed to different concentrations of LPS (0–200 μg/mL) and/or VX765 (0–10 μmol/L) for 24 h. Following treatment, CCK8 reagent was added to each well, and the plates were further incubated for 1.5 h. The absorbance at 450 nm was measured with a microplate reader (Thermo Scientific, Waltham, MA, USA). All experiments were repeated independently at least three times.

### 2.12. Establishment of the Mouse Mastitis Model

To establish the in vivo mastitis model, 12 lactating BALB/c mice (expected to deliver within one week) were used and allocated into control (Con) and LPS-treated mastitis (LPS) groups (*n* = 6 per group), as previously described [29]. After being separated from their pups for 3 h, mice were anesthetized with pentobarbital and injected with LPS (50 μL, 0.2 mg/mL) into the fourth pair of mammary glands. After 24 h, the animals were sacrificed, and their mammary tissues were harvested for subsequent analysis. For downstream molecular and histological analyses, each sample was analyzed with at least three technical replicates where applicable.

### 2.13. Statistical Analysis

Data are expressed as the mean ± SD unless otherwise stated. Experiments were conducted with at least three biological replicates, each including three technical replicates. Statistical analysis was carried out using SPSS version 23.0 (SPSS Inc., Chicago, IL, USA). Differences between the two groups were evaluated using Student’s *t*-test, whereas comparisons among multiple groups were analyzed by one-way ANOVA followed by Tukey’s multiple comparison test. For proteomics data, differential expression analysis was conducted with *p*-values adjusted using the Benjamini–Hochberg FDR correction, and adjusted *Q* < 0.05 was regarded as significant. Graphs were constructed using Prism 9.0 (GraphPad Software Inc., San Diego, CA, USA). All experiments were performed in triplicate. *p* < 0.05 was considered statistically significantly.

## 3. Results

### 3.1. Inflammatory and Pyroptotic Phenotypes in MGs of Dairy Cows with CM

**Figure 1:**
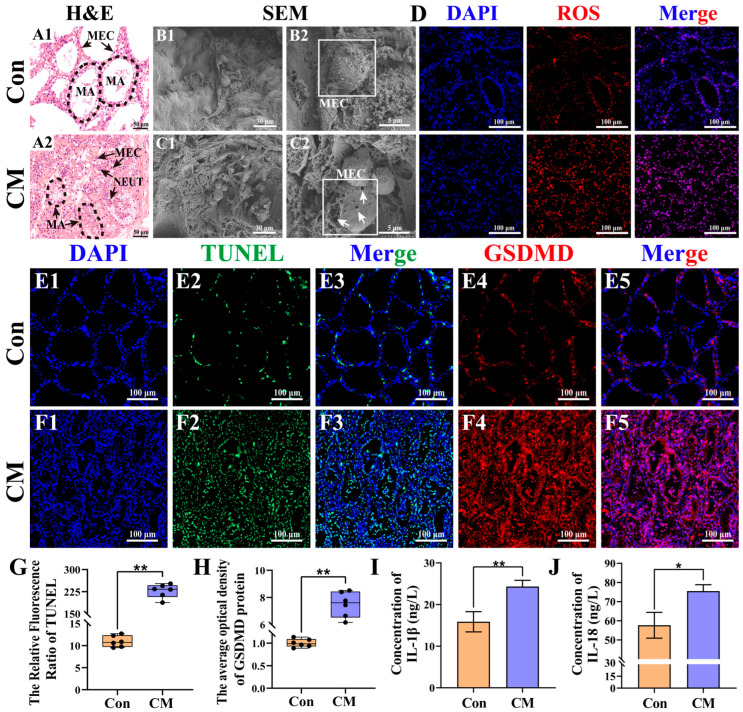
Phenotypes related to inflammation and pyroptosis in the MGs of dairy cows. (**A**) Histological examination of MGs from the Con (**A1**) and CM (**A2**) groups was performed using H&E staining. (**B**,**C**) Ultrastructural morphology of cells in the MGs of the Con (**B1**,**B2**) and CM (**C1**,**C2**) groups was observed using SEM. (**D**) ROS signals in the MGs of the Con and CM groups. (**E**,**F**) TUNEL–GSDMD staining was conducted to assess DNA fragmentation and GSDMD expression in the MGs of the Con (**E1**–**E5**) and CM (**F1**–**F5**) groups. (**G**,**H**) The relative optical density of TUNEL (**G**) and GSDMD (**H**) fluorescence from the TUNEL–GSDMD assay. (**I**,**J**) Detection of IL-1β and IL-18 protein expression in dairy serum using ELISA. MAs, mammary alveoli. MECs, mammary epithelial cells. NEUT, neutrophil. A scale bar of 100 μm, 50 μm, 30 μm, and 5 μm represents 200×, 400×, 1500×, and 6000×, respectively. Data are presented as the mean ± SEM (*n* = 3 cows per group, biological replicates), with three technical replicates for each biological sample. ** represents *p* < 0.01. * represents *p* < 0.05.

Phenotypes related to inflammation and pyroptosis were examined in the MGs of the two groups (Figure 1). H&E staining showed well-preserved mammary alveoli (MAs) with neatly arranged MECs and no histopathological signs of inflammation in the Con group (Figure 1(A1)). In contrast, MAs in the CM group appeared collapsed, accompanied by extensive MEC exfoliation and massive neutrophil infiltration, with many MECs showing cytoplasmic swelling and a loss of cellular integrity—morphological features consistent with pyroptotic cell death (Figure 1(A2)). SEM analysis showed that MECs in the Con group maintained well-preserved ultrastructural integrity, characterized by intact cell contours and continuous plasma membranes without any perforations or ruptures (Figure 1B). In contrast, the MGs from the CM group displayed a reduced number of secretory MECs, the disappearance of lipid droplets and protein particles, and disrupted cell membranes characterized by perforations and ruptures, features consistent with pyroptosis (Figure 1C). ROS fluorescence staining revealed stronger ROS signals in the CM group compared with the Con group (Figure 1D). TUNEL–GSDMD staining showed weakly positive signals in the Con group, whereas strong positive staining was observed in the CM group (Figure 1E,F). The positive rates of both TUNEL and GSDMD in the CM group were significantly higher than those in the Con group (*p* < 0.01, Figure 1G,H). Additionally, compared with the Con group, CM cows exhibited significantly increased serum levels of pyroptosis-related pro-inflammatory factors IL-1β and IL-18 (*p* < 0.01 or *p* < 0.05, Figure 1I,J). These results suggest the possible presence of inflammation and pyroptosis in the MGs of dairy cows with CM, particularly in MECs.

### 3.2. Identification of Inflammatory and Pyroptosis-Associated Candidate Proteins Based on DIA Proteomics and Bioinformatics Analysis

**Figure 2:**
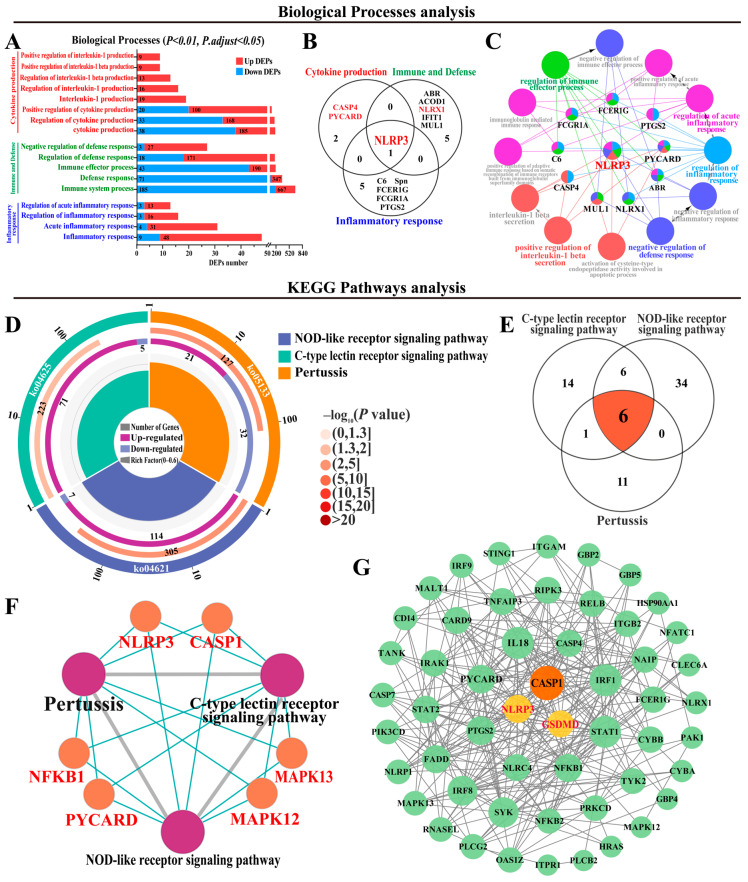
The identification of the candidate DEPs associated with inflammatory and pyroptosis based on GO terms and KEGG pathways. (**A**) The selected 17 BPs associated with cytokine production, immune and defense, and inflammatory response according to GO terms. (**B**) The Venn diagram of candidate DEPs in cytokine production, immune and defense, and inflammatory response. (**C**) The PPI network analysis of candidate BPs and DEPs. (**D**) The enrichment circle diagram of KEGG pathways related to NLRP3. (**E**) The Venn diagram of three KEGG classifications related to NLRP3. (**F**) The ClueGO analysis of six shared DEPs. (**G**) The PPI network of DEPs from the three pathways.

Based on the inflammatory and pyroptosis-like features observed in the MGs with CM, GO and pathway enrichment analyses were performed to explore DEPs potentially involved in inflammasome activation and inflammatory cell death (Figure 2). A total of eight BPs including 519 DEPs related to cytokine production, five BPs including 1402 DEPs related to immune defense, and four BPs including 108 DEPs related to inflammatory response were identified, respectively (Figure 2A, Table S5). Among these, three DEPs were shared across cytokine production; six DEPs were shared across immune defense; and six DEPs were shared across inflammatory response. Notably, NLRP3, a unique candidate DEP, was involved in all 17 BPs (Figure 2B). PPI network analysis indicated that ten DEPs directly interacted with the 12 BPs, particularly NLRP3, which plays a critical role in cytokine production, immune defense, and inflammatory response (Figure 2C). Pathway enrichment analysis further identified three NLRP3-associated pathways (NOD-like receptor signaling, C-type lectin receptor signaling, and pertussis) (Figure 2D, Table S6). A total of 72 DEPs, including 65 upregulated and seven downregulated DEPs, were identified after overlapping the shared DEPs, particularly six shared DEPs (Figure 2E). ClueGO analysis of these six shared DEPs revealed their interactions with the enriched pathways (Figure 2F). In the PPI network of these 72 DEPs, NLRP3, CASP1, and GSDMD exhibited the highest connectivity (Figure 2G), suggesting a potential involvement of the NLRP3–CASP1–GSDMD axis in CM-associated inflammatory and pyroptosis-related responses.

### 3.3. Distribution and Expression Patterns of Pyroptosis-Associated mRNAs and Proteins in the MGs of Dairy Cows

**Figure 3:**
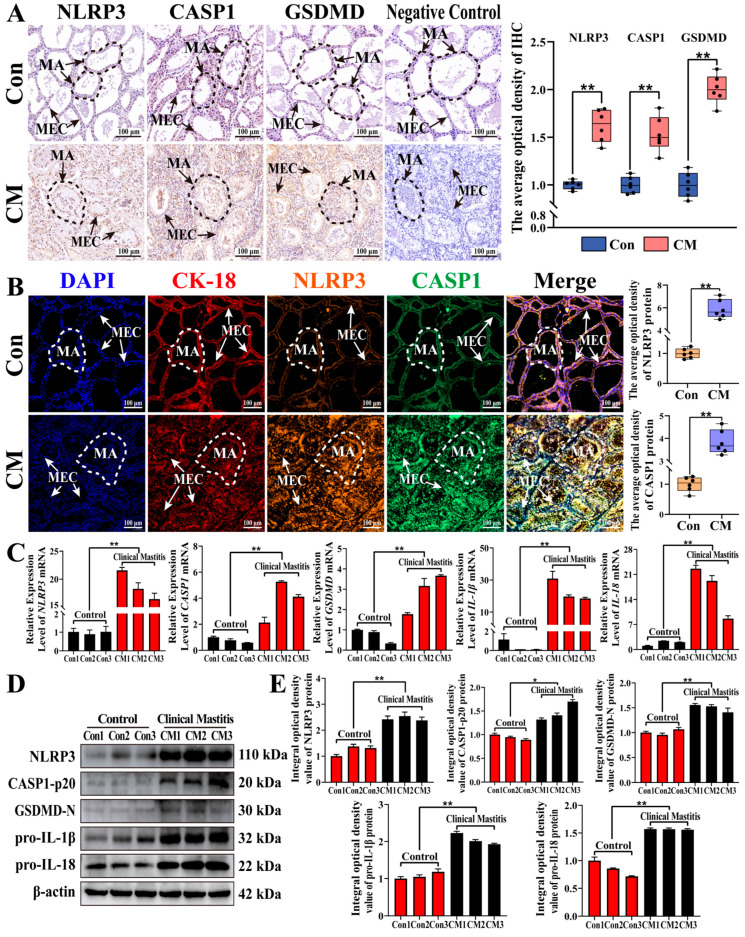
The distribution and expression pattern analysis of pyroptosis-related mRNA and proteins in the MGs. (**A**) The intracellular location analysis of NLRP3, CASP1, and GSDMD proteins in the MGs using IHC staining. (**B**) The co-localization analysis of CK-18, NLRP3, and CASP1 in the MGs using IF staining. (**C**) The mRNA expression levels of *NLRP3, CASP1*, *GSDMD*, *IL-1β*, and *IL-18* in the MGs of the Con and CM groups. (**D**,**E**) The protein expression levels of NLRP3, cleaved CASP1 (CASP1-p20), cleaved GSDMD (GSDMD-N), pro-IL-1β, and pro-IL-18 monitored by WB assay, and the relative integral optical density of bands. MAs, mammary alveoli. MECs, mammary epithelial cells. Scale bars of 100 μm represent 200×. Data are presented as the mean ± SEM (*n* = 3 cows per group, biological replicates), with three technical replicates for each biological sample. ** represents *p* < 0.01. * represents *p* < 0.05.

The distribution and expression of pyroptosis-associated mRNAs and proteins were analyzed in the MGs of the two groups (Figure 3). IHC staining demonstrated that NLRP3, CASP1, and GSDMD were mainly distributed in the cytoplasm of MECs, and their expression was significantly upregulated in the MGs of the CM group relative to the Con group, while no staining signal was detected in the negative controls (*p* < 0.01, Figure 3A). IF analysis revealed that DAPI and cytokeratin-18 (CK-18, a marker of MECs) were arranged neatly in the MAs with intact alveolar luminal areas in the Con group, whereas they appeared scattered in the MAs with disrupted alveolar structures in the CM group. CK-18, NLRP3, and CASP1 were co-localized in the cytoplasm of MECs, with significantly increased fluorescence intensities of NLRP3 and CASP1 in the CM group (*p* < 0.01, Figure 3B). Moreover, the mRNA levels of *NLRP3*, *CASP1, GSDMD*, *IL-1β*, and *IL-18* were markedly higher in the MGs from the CM group than in those from the Con group (*p* < 0.01, Figure 3C). Western blot analysis further confirmed the significant upregulation of NLRP3, cleaved CASP1 (CASP1-p20), cleaved GSDMD (GSDMD-N), pro-IL-1β, and pro-IL-18 in the CM group (*p* < 0.01, Figure 3D,E). These results indicate that NLRP3, CASP1, and GSDMD were highly expressed in the MECs of cows with CM, supporting their potential involvement in inflammasome-associated inflammatory responses and pyroptosis-like processes.

### 3.4. Expression Dynamics of Inflammasome-Associated mRNAs and Proteins and Pyroptosis-like Responses in LPS-Stimulated BMECs

**Figure 4:**
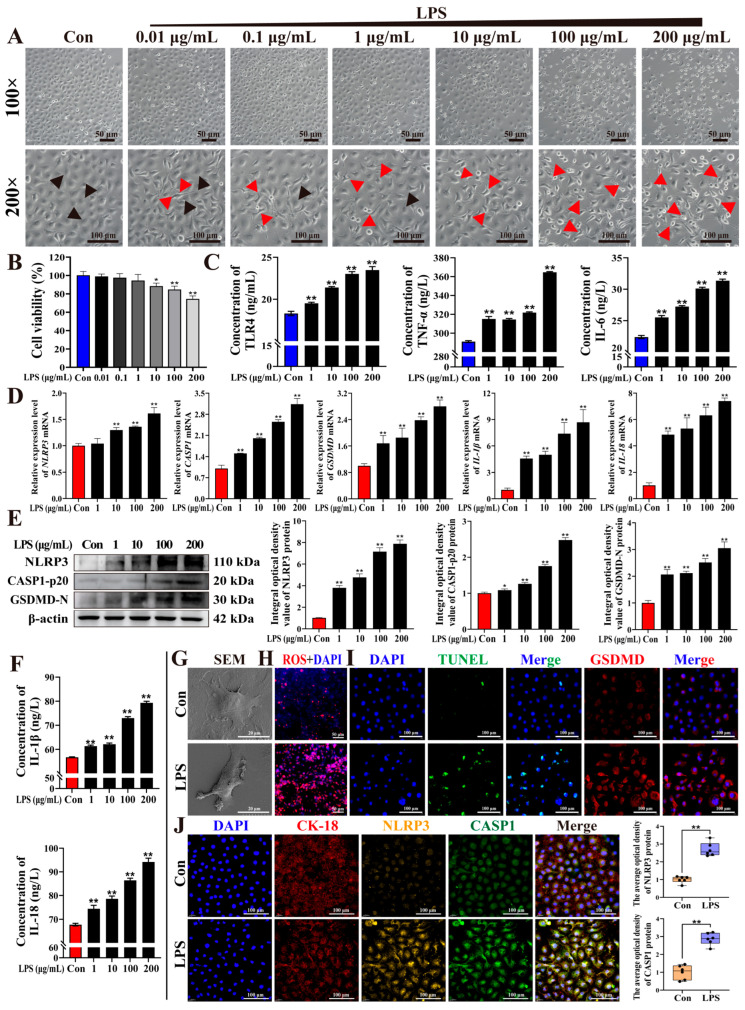
LPS-induced inflammatory injury and pyroptosis in BMECs. (**A**) Morphological changes in BMECs treated with various concentrations of LPS for 24 h. (**B**) Cytotoxicity testing of BMECs treated with LPS. (**C**) Expression levels of TLR4, TNF-α, and IL-6 in the cell supernatant of BMECs treated with LPS. (**D**) Relative mRNA expression of *NLRP3*, *CASP1*, *GSDMD*, *IL-1β*, and *IL-18* in LPS-treated BMECs determined by qPCR. (**E**) Protein levels of NLRP3, CASP1-p20, and GSDMD-N detected. (**F**) IL-1β and IL-18 levels in culture supernatants measured by ELISA. (**G**) Ultrastructural morphology of BMECs in the Con and LPS groups observed using SEM. (**H**) ROS signals in BMECs treated with or without LPS. (**I**) TUNEL–GSDMD staining assessing DNA fragmentation and GSDMD expression in BMECs. (**J**) Co-localization analysis of CK-18, NLRP3, and CASP1 in BMECs using IF staining. A scale bar of 100 μm, 50 μm, and 20 μm represents 200×, 100×, and 2000×, respectively. Experiments were performed with three independent biological replicates, each measured in triplicate technical replicates. ** represents *p* < 0.01. * represents *p* < 0.05.

To investigate the potential roles of pyroptosis in mastitis, BMECs were treated with different concentrations of LPS (Figure 4). BMECs in the Con group exhibited a typical cobblestone-like morphology with tightly arranged cells, whereas LPS-treated BMECs displayed dose-dependent morphological alterations, including cellular swelling, rounding, and detachment from neighboring cells, which are consistent with pyroptosis-associated cellular features (Figure 4A). Cytotoxicity analysis revealed a concentration-dependent decrease in cell viability following LPS treatment (0.01–200 μg/mL, Figure 4B). In parallel, the expression of pro-inflammatory mediators, including TLR4, TNF-α, and IL-6, progressively increased with rising LPS concentrations (1–200 μg/mL, Figure 4C). Moreover, the mRNA expression levels of *NLRP3*, *CASP1*, *GSDMD*, *IL-1β*, and *IL-18* were significantly increased in a dose-dependent manner following LPS stimulation (1–200 μg/mL, Figure 4D). Western blot analysis further revealed increased protein levels of NLRP3, CASP1-p20, and GSDMD-N (Figure 4E). Consistently, ELISA analysis showed significantly elevated levels of the secreted inflammatory cytokines IL-1β and IL-18 in the culture supernatants of LPS-treated BMECs (Figure 4F). Accordingly, 100 μg/mL LPS was selected to establish the inflammatory model for subsequent experiments. SEM analysis revealed that BMECs in the Con group exhibited smooth plasma membranes and abundant microvilli, indicating intact ultrastructural integrity. In contrast, LPS-treated BMECs appeared swollen and rounded, with disrupted membranes and prominent surface bubbling structures (Figure 4G). ROS staining demonstrated markedly stronger fluorescence intensity in the LPS group than in the Con group (Figure 4H). Consistently, TUNEL–GSDMD staining showed weakly positive signals in the Con group, while markedly stronger staining was detected in the LPS group (Figure 4I). IF analysis further revealed that CK-18, NLRP3, and CASP1 were co-localized in the cytoplasm of BMECs, with significantly increased fluorescence intensities of NLRP3 and CASP1 in the LPS group (*p* < 0.01, Figure 4J). Collectively, these findings indicate that LPS stimulation was associated with marked inflammatory responses in BMECs and was accompanied by activation-related changes in the NLRP3–CASP1–GSDMD signaling axis and the emergence of pyroptosis-like features.

### 3.5. Involvement of the NLRP3–CASP1 Axis in LPS-Induced Inflammasome-Associated Pyroptosis-like Responses in BMECs

**Figure 5:**
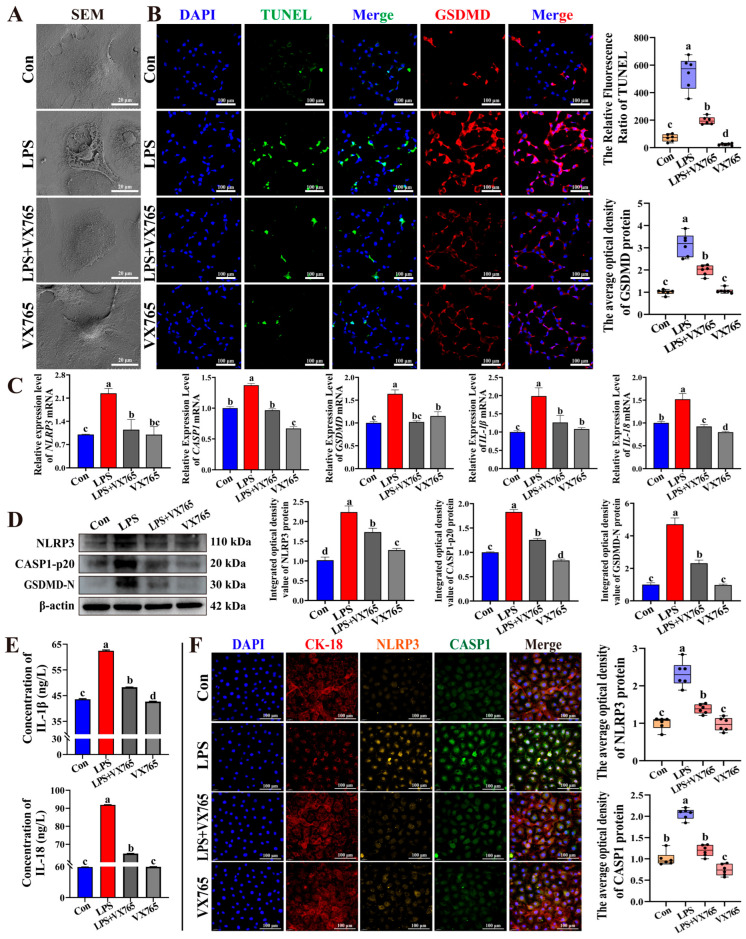
Pyroptosis activation via the NLRP3–CASP1 axis during LPS-induced inflammatory response in BMECs. (**A**) The ultrastructural morphology of BMECs observed using SEM. (**B**) TUNEL–GSDMD staining assessing DNA fragmentation and GSDMD expression in BMECs. (**C**) The relative mRNA expression of *NLRP3*, *CASP1*, *GSDMD*, *IL-1β*, and *IL-18* in BMECs treated with LPS and/or VX765 determined by qPCR. (**D**) The protein levels of NLRP3, CASP1-p20, and GSDMD-N detected by Western blot. (**E**) IL-1β and IL-18 levels in culture supernatants measured by ELISA. (**F**) The co-localization analysis of CK-18, NLRP3, and CASP1 in BMECs treated with LPS and/or VX765 using IF staining. Experiments were performed with three independent biological replicates, each measured in triplicate technical replicates. Different letters denote statistically significant differences (*p* < 0.01).

To further elucidate the role of the NLRP3–CASP1 axis in LPS-induced inflammatory injury, the selective CASP1 inhibitor VX765 was employed (Figure 5). Based on the cytotoxicity assessment, VX765 at 2.5 μmol/L was selected as the working concentration for subsequent experiments (Supplementary Figure S2). SEM analysis revealed that LPS stimulation induced prominent pyroptosis-like ultrastructural alterations, including cellular swelling, membrane disruption, and bubble-like surface protrusions, whereas these changes were markedly alleviated by VX765 treatment (Figure 5A). TUNEL–GSDMD staining revealed markedly stronger fluorescence signals in the LPS group than in the Con group, whereas VX765 treatment markedly reduced these signals (Figure 5B). Furthermore, LPS stimulation significantly upregulated the mRNA expression levels of *NLRP3*, *CASP1*, *GSDMD*, *IL-1β*, and *IL-18*, whereas VX765 treatment markedly suppressed these increases (*p* < 0.01, Figure 5C). Western blot analysis further demonstrated that the protein levels of NLRP3, CASP1-p20, and GSDMD-N induced by LPS were significantly reduced following VX765 treatment (*p* < 0.01, Figure 5D). Consistently, ELISA analysis showed that LPS-induced secretion of IL-1β and IL-18 was significantly attenuated by VX765 treatment (*p* < 0.01, Figure 5E). IF analysis further confirmed that the elevated fluorescence intensities of NLRP3 and CASP1 induced by LPS were significantly attenuated following VX765 treatment (*p* < 0.01, Figure 5F). Collectively, these findings indicate that LPS-induced inflammatory injury and pyroptosis-like responses in BMECs were partially attenuated by CASP1 inhibition and were associated with the activation of the NLRP3–CASP1–GSDMD signaling axis.

### 3.6. Evidence for Activation of NLRP3–CASP1-Associated Pyroptosis-like Signaling in an LPS-Induced Mouse Mastitis Model

**Figure 6:**
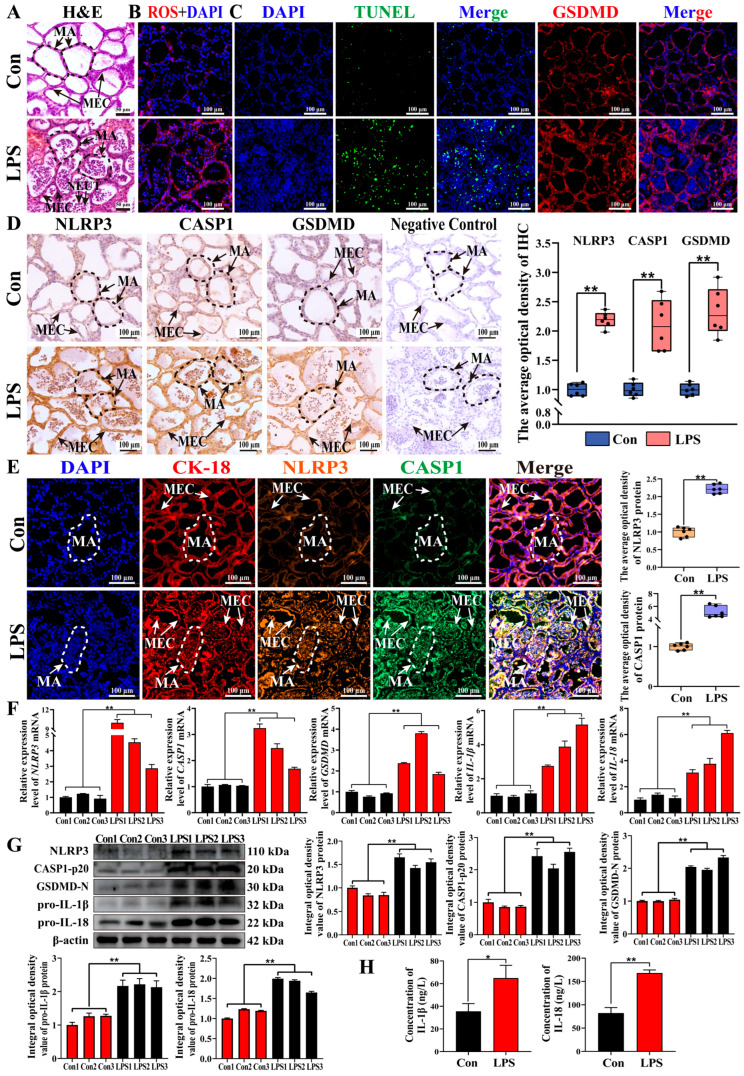
The expression and localization of pyroptosis-related targets in mouse MG tissues. (**A**) The representative images of mouse MGs stained with H&E. (**B**) The ROS staining of mouse MGs. (**C**) TUNEL–GSDMD staining was conducted to assess DNA fragmentation and GSDMD expression in mouse MGs. (**D**) The intracellular location analysis of NLRP3, CASP1, and GSDMD proteins in mouse MGs using IHC staining. (**E**) The co-localization analysis of CK-18, NLRP3, and CASP1 in mouse MG tissues using IF staining. (**F**) The mRNA expression levels of *NLRP3, CASP1*, *GSDMD*, *IL-1β*, and *IL-18* in mouse MGs. (**G**) The protein expression levels of NLRP3, CASP1-p20, GSDMD-N, pro-IL-1β, and pro-IL-18 monitored by WB assay, and the relative integral optical density of bands. (**H**) The detection of IL-1β and IL-18 protein expression in mouse serum using ELISA. MAs, mammary alveoli; MECs, mammary epithelial cells. Scale bars of 100 μm represent 200×. Data are presented as the mean ± SEM (*n* = 6 mice per group, biological replicates), with three technical replicates for each biological sample. ** represents *p* < 0.01. * represents *p* < 0.05.

The involvement of inflammasome- and pyroptosis-associated signaling was evaluated in an LPS-induced mouse mastitis model (Figure 6). H&E staining revealed intact MAs with neatly arranged MECs and no evidence of inflammation in the Con group. In contrast, the MAs in the LPS group appeared collapsed, with abundant exfoliated MECs and massive inflammatory cell infiltration (Figure 6A). The representative ROS fluorescence staining images showed a marked increase in ROS signals in the LPS group when compared with the Con group (Figure 6B). TUNEL–GSDMD staining produced weak fluorescence signals in the Con group, while markedly stronger signals were detected in the LPS group (Figure 6C). IHC analysis demonstrated that NLRP3, CASP1, and GSDMD were mainly distributed within the cytoplasm of MECs and were significantly upregulated in the MGs from LPS-treated mice, while no staining was detected in the negative controls (Figure 6D). IF staining further confirmed that CK-18, NLRP3, and CASP1 were co-localized in MECs, with stronger signals observed in the LPS group (*p* < 0.01, Figure 6E). Additionally, the LPS challenge significantly increased the mRNA expression of *NLRP3*, *CASP1*, *GSDMD*, *IL-1β*, and *IL-18* in MG tissues (*p* < 0.01, Figure 6F). Furthermore, the NLRP3, CASP1-p20, GSDMD-N, pro-IL-1β, and pro-IL-18 protein levels were significantly upregulated in the MGs of LPS-treated mice (*p* < 0.01, Figure 6G). In addition, compared with the Con group, the LPS group exhibited significantly increased serum levels of IL-1β and IL-18 (*p* < 0.01 or *p* < 0.05, Figure 6H). Collectively, these results indicate that LPS exposure is associated with the activation of the NLRP3–CASP1–GSDMD signaling pathway and pyroptosis-like features in MECs in vivo, supporting the role played by inflammasome-associated inflammatory cell death in LPS-induced mouse mastitis.

## 4. Discussion

Pyroptosis is a form of programmed cell death characterized by plasma membrane rupture and the release of inflammatory mediators [6,8,9,10]. Increasing evidence indicates that pyroptosis is closely associated with oxidative stress-driven inflammatory signaling [30], suggesting its potential involvement in the progression of bovine mastitis. Upon exposure to pathogen-associated or injury signals, often accompanied by excessive ROS accumulation, inflammatory pathways such as TLR4, NF-κB, and MAPK are activated, leading to inflammasome assembly, cytokine release, and pyroptotic cell death [17,31]. The subsequent release of inflammatory mediators further amplifies immune cell activation, thereby establishing a feed-forward amplification loop between oxidative stress and inflammation, which may ultimately contribute to epithelial barrier disruption and immune dysregulation [5,32]. Pyroptosis-associated tissue injury is typically accompanied by characteristic histopathological changes, including cellular swelling, membrane perforation [9,10], and increased ROS and TUNEL–GSDMD positivity [33]. These features are consistent with our histopathological observations, supporting a close association between mastitis-related inflammation and pyroptosis. Therefore, elucidating the mechanisms underlying pyroptosis activation may facilitate the development of targeted therapeutic strategies for mastitis.

Bioinformatic analyses based on DIA proteomic data identified NLRP3 as a central node associated with cytokine production, immune defense, and inflammatory responses. Notably, NLRP3 is a redox-sensitive inflammasome sensor that integrates pathogen-derived stimuli with oxidative stress-induced cellular damage [7,11,12]. Consistent with this role, NLRP3 activation has been shown to facilitate inflammasome assembly, which triggers CASP1 activation, the maturation of pro-inflammatory cytokines, and ultimately pyroptotic cell death with the release of inflammatory mediators [34]. These observations support the involvement of NLRP3 in MEC pyroptosis and inflammatory responses during bovine CM. Pathway enrichment further linked NLRP3 to the NOD-like receptor (NLR) signaling pathway and the C-type lectin receptor (CLR) signaling pathway, suggesting that multiple innate immune pathways converge on NLRP3 inflammasome priming and activation. Upon pathogen invasion or cellular damage, oxidative stress-amplified DAMPs or PAMPs activate inflammasomes, including NLRP3, leading to CASP1-mediated pyroptosis [35]. In parallel, CLRs further promote NLRP3 activation through NF-κB and MAPK signaling, enhancing pro-inflammatory cytokine release and immune responses, thus reinforcing the oxidative stress–inflammation feedback loop [17,31]. Collectively, the results support the involvement of NLRP3 in MEC pyroptosis and in regulating tissue homeostasis and immune responses during mastitis. Additionally, PPI and ClueGO analyses revealed interactions between NLRP3 and key inflammatory regulators, such as MAPK12, MAPK13, prostaglandin-endoperoxide synthase 2 (PTGS2), and signal transducer and activator of transcription 1 (STAT1), which may further enhance NLRP3-associated inflammatory responses [36,37,38]. Importantly, CASP1, activated downstream of NLRP3, cleaves pro-IL-1β and pro-IL-18 into their active forms and processes GSDMD to form membrane pores, thereby promoting pyroptotic cell death and exacerbating tissue inflammation [9,10]. Collectively, these findings suggest that NLRP3–CASP1–GSDMD activation represents a critical molecular axis contributing to inflammatory injury during clinical mastitis.

Previous studies have highlighted the pivotal roles of the NLRP3–CASP1–GSDMD axis in diverse inflammatory diseases. For example, NLRP3 activation in immune cells promotes cytokine release, endothelial injury, and plaque development, thereby accelerating the progression of atherosclerosis [39]. In Crohn’s disease and ulcerative colitis, NLRP3 activation has been shown to promote pyroptosis in intestinal epithelial cells, enhancing immune responses and sustaining inflammation [40]. Similarly, CASP1 activation exacerbates cardiac inflammation and ischemic injury following myocardial infarction [41], whereas GSDMD activation has been implicated in intensifying neuroinflammation and neurodegeneration in Alzheimer’s disease [42]. Collectively, these observations support the concept that NLRP3-driven inflammasome signaling constitutes a conserved mechanism of inflammatory injury across tissues. In our study, NLRP3 and CASP1 were localized in the cytoplasm of MECs. Increased TUNEL–GSDMD-positive cells and the activation of pyroptosis-related targets in the MG tissues of the CM group suggest that the NLRP3–CASP1 axis may contribute to MEC dysfunction and MG inflammation under oxidative stress. These findings further support the involvement of NLRP3–CASP1 upregulation in mediating MEC pyroptosis and inflammation.

In the LPS-induced BMEC inflammation model, we observed cellular swelling, rounding, detachment, and the upregulation of pyroptosis-related targets and inflammatory mediators. Notably, the pharmacological inhibition of CASP1 markedly attenuated these alterations and reduced NLRP3 expression, suggesting that CASP1 activity may contribute to sustaining NLRP3 activation and establishing a feed-forward inflammatory circuit. Previous studies have shown that CASP1 inhibition can reduce NF-κB and activator protein-1 (AP-1) activity, thereby indirectly suppressing *NLRP3* transcription and impairing IL-18 maturation, leading to the inhibition of JAK–STAT signaling [43,44,45]. These findings support an important role for CASP1 in regulating NLRP3-mediated inflammasome signaling and pyroptosis during mastitis. Consistent with this concept, CASP1 activation has been reported to promote pyroptosis and enhance IL-1β and IL-18 release in acute pancreatitis and diabetes, thereby exacerbating inflammation and tissue injury [46,47]. In addition, CASP1 can cleave poly (ADP-ribose) polymerase-1 (PARP1), disrupting DNA repair and enhancing cell death, which further amplifies inflammatory damage [48]. These observations are in line with the increased expression of pyroptosis-related targets and enhanced TUNEL–GSDMD positivity observed in the murine mastitis model. Recent studies have further demonstrated that the inhibition of CASP1 activity using small molecules or gene-editing approaches reduces IL-1β and IL-18 secretion, alleviates inflammation, and improves disease outcomes in autoimmune and chronic inflammatory disorders [43,44,45]. Collectively, these results emphasize the potential importance of the NLRP3–CASP1 axis in MEC pyroptosis and mastitis-associated inflammation and provide a mechanistic basis for the development of targeted therapeutic strategies.

**Figure 7:**
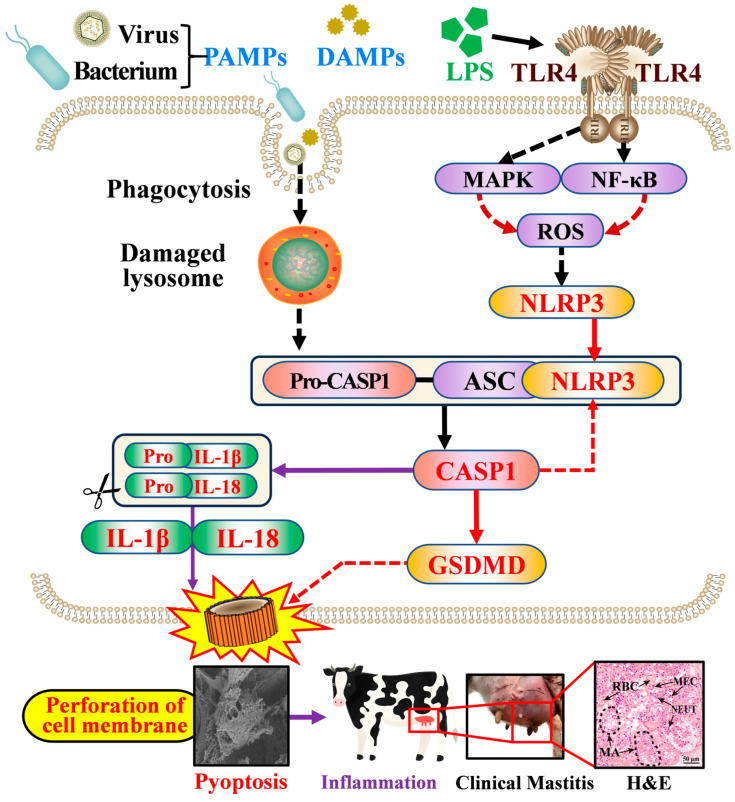
The proposed mechanism by which the NLRP3–CASP1 axis promotes inflammation in bovine mastitis via GSDMD activation through the canonical pyroptosis pathway.

Taking these results together with those of previous studies, the potential mechanism underlying NLRP3–CASP1-mediated pyroptosis during mastitis is supported (Figure 7). Pathogen-derived PAMPs and host-derived DAMPs may be internalized through phagocytosis, leading to lysosomal destabilization and the release of intracellular danger signals that promote NLRP3 inflammasome activation [7,9]. In parallel, Gram-negative bacterial LPS is recognized by TLR4, triggering the activation of the NF-κB and MAPK signaling pathways, which promote NLRP3 priming and activation, a process further amplified by ROS accumulation [11]. However, for Gram-positive bacteria, such as *Staphylococcus aureus*, immune activation occurs through the recognition of peptidoglycan by TLR2, leading to NF-κB and MAPK activation, which similarly contributes to inflammasome priming and the subsequent inflammatory response. Upon activation, NLRP3 assembles with ASC/Pycard and pro-CASP1 to form the inflammasome complex, leading to CASP1 activation. Activated CASP1 subsequently cleaves GSDMD, generating the N-terminal fragment (GSDMD-N), which forms pores in the plasma membrane, thereby promoting membrane rupture, cellular swelling, and pyroptotic cell death [9,15,42]. Meanwhile, CASP1 processes pro-IL-1β and pro-IL-18 into their mature forms, thereby amplifying inflammatory responses and tissue injury and ultimately contributing to the development of clinical mastitis. Notably, the inhibition of key mediators within this pathway, either pharmacologically or genetically, has been reported to suppress canonical pyroptosis [43,44,45], supporting the therapeutic potential of targeting the NLRP3–CASP1 axis for mastitis intervention.

This study has several limitations. First, although the basic pathological response of mastitis is similar, different etiological agents may result in differences in the specific mechanisms and outcomes of the immune response. All CM cases included in this study were attributable to *Escherichia coli*, potentially limiting the extrapolation of our findings to mastitis caused by other etiological agents. Additionally, the small sample size (*n* = 3 per group) may have limited statistical power and the ability to capture biological variability among animals. Second, although bioinformatics analyses highlighted the NLRP3–CASP1–GSDMD axis as a core mechanism underlying pyroptosis in *Escherichia coli*-induced bovine mastitis, we did not specifically investigate the non-canonical pyroptosis pathway mediated by CASP4 [8,15]. Third, further in vitro and in vivo studies are required to evaluate NLRP3–CASP1-targeted interventions and to more fully elucidate the dynamic interplay between oxidative stress and pyroptosis during mastitis progression. Nonetheless, our findings underscore the involvement of NLRP3–CASP1-mediated MEC pyroptosis, providing new insights into the molecular pathogenesis of bovine mastitis and offering a conceptual framework for the development of targeted therapeutic strategies and drug discovery.

## 5. Conclusions

In this study, pyroptosis-like features were observed in the MG tissues of Holstein cows affected by CM. The bioinformatics analysis of DIA proteomic data identified 17 BPs and 276 DEPs associated with cytokine production, inflammatory response, and immune defense. Pathway analysis suggested that NLRP3, as a key candidate target, may activate GSDMD via the canonical CASP1-mediated pyroptosis pathway, thereby promoting MEC pyroptosis and MG tissue inflammation in CM-affected cows. Elevated levels of TUNEL–GSDMD significantly upregulated the mRNA expression of *NLRP3*, *CASP1*, *GSDMD*, *IL-1β*, and *IL-18*, and increased protein levels of NLRP3, CASP1-p20, GSDMD-N, pro-IL-1β, and pro-IL-18 were observed in CM-affected MGs. Results from both the in vitro and in vivo models showed the significant upregulation of pyroptotic proteins and pro-inflammatory cytokines in the inflammation model group. Pharmacological CASP1 inhibition attenuated pyroptosis-associated changes and inflammatory responses, suggesting that CASP1 is involved in these processes. Collectively, these findings provide new insights into inflammasome-associated mechanisms in CM and support the potential relevance of the NLRP3–CASP1 axis as a candidate therapeutic target for bovine mastitis.

## Data Availability

The original contributions presented in this study are included in the article/Supplementary Materials. Further inquiries can be directed to the corresponding author.

## Supplementary Materials

The following supporting information can be downloaded at: https://www.mdpi.com/article/10.3390/antiox15030385/s1, Figure S1: Pathogen isolation and identification. Figure S2: Cytotoxicity assessment of VX765 using CCK-8. Table S1: Composition of the total mixed ration (TMR) for Holstein cows. Table S2: Milk quality analysis and sample screening. Table S3: Antibodies and dilution ratio. Table S4: qPCR primer information. Table S5: DIA proteomic BPs analysis. Table S6: DIA proteomic pathways analysis.

## Abbreviations

The following abbreviations are used in this manuscript:

CM	Clinical mastitis
MG	Mammary gland
GSDMD	Gasdermin-D
IL	Interleukin
MECs	Mammary epithelial cells
NLRP3	NOD-like receptor family pyrin domain-containing 3
PAMPs	Pathogen-associated molecular patterns
DAMPs	Damage-associated molecular patterns
ROS	Reactive oxygen species
CASP1	Caspase-1
LPS	Lipopolysaccharide
CASP4	Caspase-4
NF-κB	Nuclear factor kappa-light-chain-enhancer of activated B cells
MAPK	Mitogen-activated protein kinase
DIA	Data-independent acquisition
DEP	Differentially expressed protein
BMECs	Bovine mammary epithelial cells
TMR	Total mixed ration
CMT	California Mastitis Test
SCC	Somatic cell count
DSCC	Differential somatic cell count
H&E	Hematoxylin and eosin
SEM	Staining and scanning electron microscope
IF	Immunofluorescence
OCT	Optimal cutting temperature
DAPI	4′,6-Diamidino-2-phenylindole
ELISA	Enzyme-linked immunosorbent assay
TLR4	Toll-like receptor 4
TNF-α	Tumor necrosis factor-alpha
GO	Gene Ontology
KEGG	Kyoto Encyclopedia of Genes and Genomes
BP	Biological processes
PPI	Protein–protein interaction
IHC	Immunohistochemistry
SABC	Streptavidin–biotin complex
DAB	3-diaminobenzidine
PBS	Phosphate buffered saline
qPCR	Quantitative polymerase chain reaction
BCA	Bicinchoninic acid
IOD	Integrated optical density
CCK8	Cell counting kit 8
MA	Mammary alveoli
NEUT	Neutrophil
CK-18	Cytokeratin-18
CLR	C-type lectin receptor
PTGS2	Prostaglandin-endoperoxide synthase 2
STAT1	Signal transducer and activator of transcription 1
AP-1	Activator protein-1
