# Supplementary material for: The NLRP3–CASP1 Axis Contributes to Pyroptosis in Bovine Mammary Epithelial Cells During Clinical Mastitis
Source: Antioxidants (Basel). 2026 Mar 19;15(3):385. doi: 10.3390/antiox15030385 (PMC13023787; doi:10.3390/antiox15030385)
Supplement: Supplementary file 1 [file antioxidants-15-00385-s001.zip › antioxidants-4116480-supplementary.pdf]

Figure S1. Pathogen isolation and identification

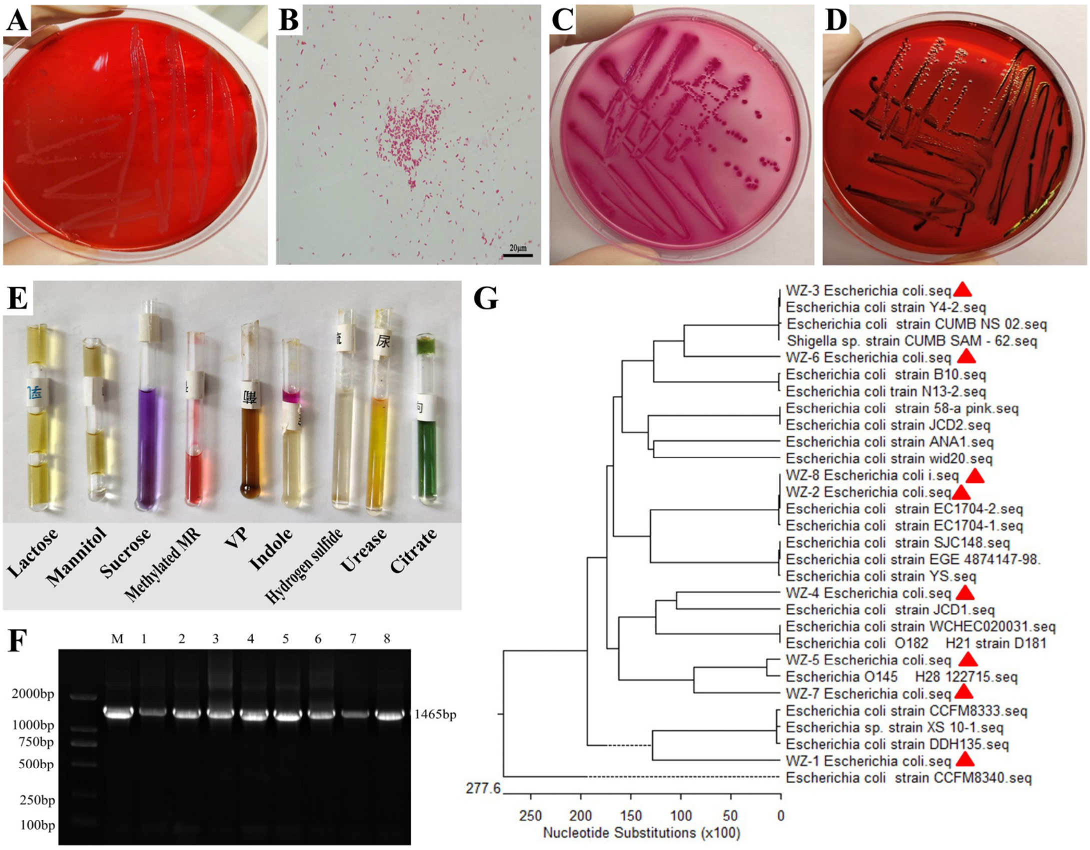

Figure S1. Pathogen isolation and identification. **(A)** Detect colony morphology on blood agar plates. **(B)** Gram staining of Escherichia coli. **(C)** MacConkey agar medium for detecting the morphology of Escherichia coli. **(D)** Eosin-methylene blue staining for detecting the morphology of Escherichia coli. **(E)** Biochemical identification results of Escherichia coli strains. **(F)** 16S amplification results of the strain. **(G)** Phylogenetic tree analysis of Escherichia coli.

- The milk samples were cultured on different agar media. The results showed that colorless, transparent, single colonies with neat edges were successfully purified on the blood agar plate, indicating that the strain had been purified successfully (Figure S1A). Gram staining under the microscope showed pink-colored (Gram-negative) bacteria with blunt-ended, short rod shapes, suggesting that the strain was Escherichia coli (Figure S1B). Using selective media, 8 strains were found to grow on MacConkey agar, producing smooth, raised, pink colonies (Figure S1C). On eosin methylene blue (EMB) agar, black colonies with a metallic sheen were observed (Figure S1D).

- Biochemical identification of the 8 suspected Escherichia coli strains was performed using biochemical tubes (Figure S1E). The results showed that the biochemical tubes for lactose and mannitol turned from purple to yellow, with gas bubbles formed, indicating that the strains could utilize lactose and mannitol to produce acid and gas; however, no color change or gas formation occurred in the sucrose tube, indicating the strains did not utilize sucrose. The methyl red test turned red, indicating that the bacteria fermented sugar in the medium, producing a large amount of acid, while the VP test showed no color change. The indole test turned red, indicating that the bacteria broke down tryptophan to produce indole. No color change was observed in the hydrogen sulfide and urease tests, indicating that the strains did not break down urea. The citrate utilization test did not change color, suggesting the strains did not utilize citrate.
- PCR amplification was performed using bacterial 16S universal primers (Figure S1F), and the expected bands were clearly present. BLAST sequence comparison of the results showed that all 8 strains were Escherichia coli. The 16S rRNA sequences of the isolated 8 strains were named WZ-1 Escherichia coli, WZ-2 Escherichia coli, WZ-3 Escherichia coli, and WZ-8 Escherichia coli. The sequencing results were compared with 24 other Escherichia coli strains with 99.8%–100% homology, and a phylogenetic tree was constructed using DNAs-star software (Figure S1G).
- **These results suggest that Escherichia coli may be the primary pathogen responsible for bovine mastitis, providing important microbiological evidence for the development of clinical treatment and preventive measures.**

Figure S2. Cytotoxicity assessment of VX765 using CCK-8

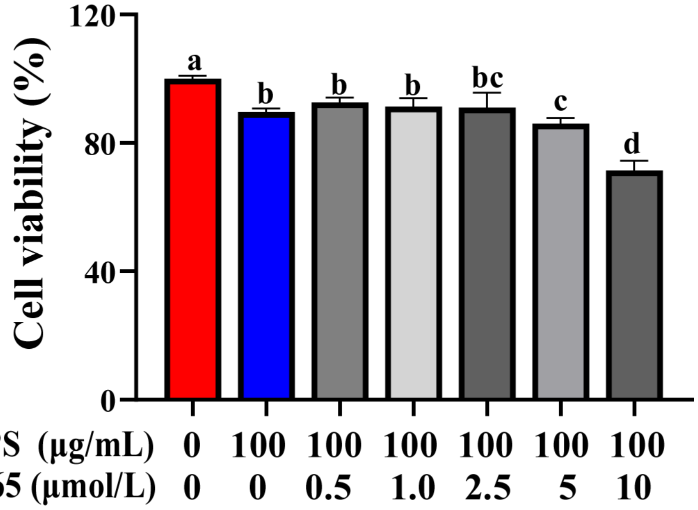

Figure S2. Cytotoxicity assessment of VX765 using CCK-8.

- Based on cytotoxicity assessment, VX765 at 2.5 μmol/L was selected as the working concentration for subsequent experiments (Supplementary Fig.S2).

Table S1 Composition of the total mixed ration (TMR) for Holstein cows

(A) Composition of the concentrate mixture for lactating Holstein cows (per 1000 kg diet)

Ingredient	Amount (kg)	Crude protein (kg)
Corn	520.0	38.0
Soybean meal	150.0	64.5
Rapeseed meal	80.0	24.0
Cottonseed meal	60.0	25.8
Wheat bran	70.0	12.0
Beet pulp	40.0	0.0
Milk enhancer	15.0	0.0
Salt	10.0	0.0
Sodium bicarbonate	15.0	0.0
Magnesium oxide	3.0	0.0
Anti-mold agent	1.0	0.0
Premix	10.0	0.0
**Total**	**1000.0**	**174.3**

Note: The crude protein content of this concentrate mixture is 17.4%.
Energy: Moderate level (NEL=6.8 MJ/kg DM).

(B) Total Mixed Ration (TMR) Formula for Lactating Cows (kg/day)

Ingredient	Amount (kg)	Dry Matter (kg)
Concentrate	9.5	9.0
Corn silage	4.5	4.0
Wheat straw	3.0	0.0
Green hay	17.0	5.0
Apple pomace	1.5	1.4
Distillery by-products	8.0	2.4
Soybean meal	3.0	1.2
**Total**	**50.0**	**24.6**

Note: This TMR formula is suitable for lactating cows producing between 23-28 kg of milk per day.
Feeding Sequence: The recommended feeding sequence is as follows: silage → hay → concentrate mixture, ensuring stable rumen pH.
Additive Recommendations: It is advised to include 100 g/head/day of sodium bicarbonate and 30 g of magnesium oxide in the concentrate to further prevent acidosis.
Vitamin and Mineral Premix: Supplementation of 80,000 IU of vitamin A, 20,000 IU of vitamin D, and 600 mg of vitamin E per head per day is recommended.
Moisture Control: Due to the high moisture content of soybean meal and brewery grains, proper measures should be taken to prevent mold and spoilage.

Table S2 Milk Quality Analysis and Sample Screening

				Milk quality analysis RAW data														CMT analysis RAW data					Result	Sample selection
No	postpartum	Date	Time	Temp	Fat	Prot.Cru.	Lactose	Total Solids	Conductivity	H.index	Acetone	BHB	BUN	SCC (cells/mL)	SCC Analysis	DSCC (%)	DSCC Analysis	Left Fore	Right Fore	Left Hind	Right Hind	CMT Analysis	Omprehensive analysis	
Con-1	40 days	2022/1/17	17:06:28	39.08	1.14	3.18	5.4	10.45	847.8	0.52	0.19	0.14	10.5	4000		0						Health	Health	Con-1 (Con group)
Con-2	40 days	2022/1/17	17:05:28	36.85	1.31	3.82	5.58	11.58	830	0.42	0.21	0.14	13.8	5000		0						Health	Health	Con-2 (Con group)
Con-3	15 days	2022/1/17	16:24:37	41.61	1.66	3.52	5.48	11.46	813.9	0.45	0.15	0.13	17.4	9000		0						Health	Health	Con-3 (Con group)
CM-1	40 days	2022/1/17	16:58:40	36.78	3.6	4.1	3.78	12.44	1216.7	0.88	0.07	0.16	17.6	33719000	Clinical mastitis	86.9	Clinical mastitis		+++	++		Clinical mastitis	Clinical mastitis	CM-1 (CM group)
CM-2	40 days	2022/1/17	16:48:35	39.08	1.54	3.16	4.67	10.02	1151.7	0.8	0.14	0.19	8	5911000	Clinical mastitis	86.3	Clinical mastitis	+++	++	+++	++	Clinical mastitis	Clinical mastitis	CM-2 (CM group)
CM-3	15 days	2022/1/17	16:27:01	40.09	1.66	3.36	5.32	11.08	887.9	0.51	0.11	0.1	9.6	3142000	Clinical mastitis	84.6	Clinical mastitis			+++	++	Clinical mastitis	Clinical mastitis	CM-3 (CM group)
1	15 days	2022/1/17	16:20:17	38.11	2.5	3.21	5.34	11.7	818.6	0.55	0.09	0.12	12.5	35000		0						Health	Health	
2	15 days	2022/1/17	16:20:23	41.27	2.44	3.8	5.22	12.24	866.6	0.55	0.12	0.09	12.4	85000		61.7	Mild inflammation					Health	Suspected	
3	15 days	2022/1/17	16:20:35	41.91	3.16	3.93	5.37	13.4	752.1	0.44	0.15	0.15	15.3	42000		0				+		Suspected	Suspected	
4	15 days	2022/1/17	16:20:47	41.75	3.53	4.61	4.85	14.13	843	0.51	0.1	0.1	10.7	157000		55.7			+			Suspected	Suspected	
5	15 days	2022/1/17	16:20:59	41.69	3.03	3.75	5.27	12.92	790.8	0.49	0.1	0.12	9.5	22000		0						Health	Health	
6	15 days	2022/1/17	16:21:11	41.73	1.89	3.82	5.32	11.83	874.1	0.5	0.1	0.09	18.4	77000		65.8	Mild inflammation			+	+	Suspected	Suspected	
7	15 days	2022/1/17	16:21:22	42.45	0.64	3.76	5.02	10.34	970.9	0.55	0.2	0.05	11.7	236000	Mild inflammation	66.1	Mild inflammation		+++	+++	++	Clinical mastitis	subclinical mastitis	
8	15 days	2022/1/17	16:21:34	41.9	2.68	4.37	4.76	12.83	989.2	0.6	0.02	0.06	10.9	832000	Clinical mastitis	84.8	Clinical mastitis	+	++	+++	+++	Clinical mastitis	Clinical mastitis	
9	15 days	2022/1/17	16:21:46	41.32	2.83	3.6	5.27	12.47	823.8	0.51	0.05	0.08	15.8	29000		0						Health	Health	
10	15 days	2022/1/17	16:21:49	40.86	1.64	3.29	5.04	10.74	922.9	0.59	0.46	0.27	9.2	166000		73.2	Mild inflammation	+	+	++	+	subclinical mastitis	Subclinical mastitis	
11	15 days	2022/1/17	16:22:01	41.58	4.01	3.44	5.15	13.35	835	0.49	0.31	0.25	12.4	100000		62	Mild inflammation			+	++	subclinical mastitis	Subclinical mastitis	
12	15 days	2022/1/17	16:22:13	41.46	2.57	3.8	5.31	12.58	791.5	0.46	0.2	0.16	11.7	30000		0				+		Suspected	Suspected	
13	15 days	2022/1/17	16:22:25	42.18	0.95	3.65	5.04	10.48	925.7	0.59	0.24	0.11	5.9	56000		51.2			++			subclinical mastitis	Suspected	
14	15 days	2022/1/17	16:22:37	41.81	2.01	3.77	5.33	11.89	876.3	0.46	0.19	0.12	13.5	12000		0		+	+			Suspected	Suspected	
15	15 days	2022/1/17	16:22:49	41.6	1.01	3.52	5.42	10.78	833	0.46	0.29	0.19	14	24000		0						Health	Health	
16	15 days	2022/1/17	16:23:01	41.39	3.13	3.38	5.19	12.47	812.8	0.49	0.18	0.16	11.8	51000		28.1						Health	Health	
17	15 days	2022/1/17	16:23:13	41.1	2.03	3.8	5.29	11.96	835.4	0.48	0.14	0.12	14	27000		0						Health	Health	
18	15 days	2022/1/17	16:23:25	41.12	1.65	3.34	5.6	11.32	792.4	0.44	0.21	0.17	15.3	14000		0						Health	Health	
19	15 days	2022/1/17	16:23:37	40.87	7.23	4.28	4.4	17.11	941.6	0.77	1.34	0.31	14	656000	Clinical mastitis	43.7		+++	+	+	++	Clinical mastitis	Suspected clinical mastitis	
20	15 days	2022/1/17	16:23:49	41.21	1.34	3.21	5.3	10.48	887.4	0.51	0.25	0.14	11.2	36000		0						Health	Health	
21	15 days	2022/1/17	16:24:01	41.88	3.26	3.53	5.41	13.09	760.4	0.44	0.31	0.23	11.3	75000		66.4	Mild inflammation					Health	Suspected	
22	15 days	2022/1/17	16:24:13	41.47	1.84	3.66	5.25	11.59	897.9	0.47	0.19	0.14	12.6	11000		0						Health	Health	
23	15 days	2022/1/17	16:24:25	41.58	2.49	3.83	5.11	12.31	886.1	0.49	0.19	0.17	13.2	40000		0		++		+		subclinical mastitis	Suspected	
24	15 days	2022/1/17	16:24:49	41.18	3.28	4.09	4.62	12.95	990.3	0.66	0	0.04	10.5	279000	Mild inflammation	74.3	Mild inflammation					Health	Subclinical mastitis	
25	15 days	2022/1/17	16:25:01	41.13	2.52	3.78	4.89	12.04	939.1	0.55	0.15	0.13	12.7	57000		61.4	Mild inflammation	+				Suspected	Suspected	
26	15 days	2022/1/17	16:25:13	41.13	2.15	3.88	4.72	11.64	1027.8	0.64	0.12	0.11	4.8	101000		56.1						Health	Health	
27	15 days	2022/1/17	16:25:25	40.26	10.06	4.12	3.48	18.81	1120.5	1.09	0.22	0.31	15.8	1834000	Clinical mastitis	82.2	Clinical mastitis	+	+++	++	+++	Clinical mastitis	Clinical mastitis	
28	15 days	2022/1/17	16:25:37	40.27	2.84	3.94	4.74	12.4	967.5	0.62	0.04	0.06	15.2	63000		57.1						Health	Health	
29	15 days	2022/1/17	16:25:49	40.55	1.83	3.66	5.31	11.6	840.7	0.49	0.12	0.11	14.8	32000		0						Health	Health	
30	15 days	2022/1/17	16:26:01	40	2.18	4.29	4.85	12.37	966.9	0.58	0.15	0.08	10.7	690000	Clinical mastitis	76.6	Clinical mastitis			+++	+	Clinical mastitis	Clinical mastitis	
31	15 days	2022/1/17	16:26:13	40.49	2.28	3.51	5.2	11.84	862.6	0.5	0.17	0.17	15.9	36000		0						Health	Health	
32	15 days	2022/1/17	16:26:25	40.47	3.69	3.97	5.04	13.8	810.2	0.43	0.31	0.22	11.9	26000		0						Health	Health	
33	15 days	2022/1/17	16:26:37	40.46	2.27	3.46	5.43	11.97	809.7	0.46	0.2	0.19	10.8	29000		0						Health	Health	
34	15 days	2022/1/17	16:26:49	40.12	3.65	3.69	5.27	13.39	814.1	0.5	0.11	0.13	11.6	91000		47						Health	Health	
35	15 days	2022/1/17	16:27:13	39.89	2.64	3.81	4.74	12.09	1005.6	0.68	0.09	0.09	6.7	132000		51.2						Health	Health	
36	15 days	2022/1/17	16:27:25	39.57	5.07	3.22	4.81	13.73	859	0.57	0.38	0.22	11.7	71000		65.5	Mild inflammation					Health	Suspected	
37	15 days	2022/1/17	16:27:37	39.5	2.85	3.54	5.17	12.3	829.1	0.51	0.15	0.14	10.7	32000		0						Health	Health	
38	40 days	2022/1/17	16:46:59	40.34	1.98	2.94	5.13	10.48	952.7	0.64	0.1	0.12	11.6	15000		0						Health	Health	
39	40 days	2022/1/17	16:47:11	40.16	1.58	3.47	5.35	11.18	879.8	0.5	0.11	0.14	11.8	29000		0						Health	Health	
40	40 days	2022/1/17	16:47:23	40.54	2.6	3.64	4.95	11.88	967.7	0.63	0.05	0.1	10.9	106000		63.6	Mild inflammation	++	+			subclinical mastitis	Subclinical mastitis	
41	40 days	2022/1/17	16:47:35	40.38	2.9	3.34	5.46	12.42	806.5	0.5	0.1	0.15	12.1	20000		0						Health	Health	
42	40 days	2022/1/17	16:47:47	40.01	1.24	3.41	5.3	10.66	918.8	0.55	0.15	0.12	10.6	15000		0						Health	Health	
43	40 days	2022/1/17	16:47:59	40.04	4.58	3.4	5.16	13.87	836.3	0.59	0.08	0.12	9.9	28000		0						Health	Health	
44	40 days	2022/1/17	16:48:11	39.73	3.39	2.98	5.33	12.33	814.4	0.49	0.2	0.19	7.1	79000		44.7		+				Suspected	Suspected	
45	40 days	2022/1/17	16:48:23	39.92	0.52	3.03	5.39	9.57	909.1	0.52	0.27	0.16	13.7	14000		0						Health	Health	
46	40 days	2022/1/17	16:48:47	38.54	3.5	2.8	5.35	12.24	806.3	0.54	0.09	0.18	10.2	62000		65.8	Mild inflammation					Health	Suspected	
47	40 days	2022/1/17	16:48:59	39.47	3.18	2.69	4.94	11.3	966.6	0.66	0.09	0.13	12.3	36000		0						Health	Health	
48	40 days	2022/1/17	16:49:11	39.27	2.77	3.22	4.73	11.19	1055.1	0.71	0.22	0.28	15.3	257000	Mild inflammation	46.7			++	++	++	subclinical mastitis	Subclinical mastitis	
49	40 days	2022/1/17	16:49:23	39.44	1.7	3.33	5.44	11.15	874.1	0.52	0.16	0.14	9.5	20000		0						Health	Health	
50	40 days	2022/1/17	16:49:35	39.21	2.46	3.27	5.42	11.83	853.3	0.56	0.07	0.12	10.9	13000		0						Health	Health	
51	40 days	2022/1/17	16:49:47	39.08	1.74	3.4	5.42	11.29	845.3	0.52	0.24	0.2	9.7	94000		73	Mild inflammation					Health	Suspected	
52	40 days	2022/1/17	16:49:59	38.83	1.48	3.44	5.44	11.14	872.5	0.49	0.19	0.15	5.9	75000		63.2	Mild inflammation				++	subclinical mastitis	Subclinical mastitis	
53	40 days	2022/1/17	16:50:11	38.82	1.94	3.56	5.48	11.73	812.9	0.51	0.12	0.12	8.4	56000		71.7	Mild inflammation			+	+	Suspected	Suspected	

No	postpartum	Date	Time	Temp	Fat	Prot.Cru.	Lactose	Total Solids	Conductivity	H.index	Acetone	BHB	BUN	SCC (cells/mL)	SCC Analysis	DSCC (%)	DSCC Analysis	Left Fore	Right Fore	Left Hind	Right Hind	CMT Analysis	Omprehensive analysis	Sample selection
				Milk quality analysis RAW data														CMT analysis RAW data					Result	
54	40 days	2022/1/17	16:50:23	37.75	1.72	3.04	5.28	10.67	919.9	0.59	0.15	0.15	7.7	62000		46.5				+		Suspected	Suspected	
55	40 days	2022/1/17	16:50:35	37.45	1.68	3.16	5.29	10.79	878.8	0.58	0.2	0.17	11.7	203000	Mild inflammation	63.3	Mild inflammation				+	Suspected	Subclinical mastitis	
56	40 days	2022/1/17	16:50:47	39.93	0.88	3.49	5.6	10.7	866.3	0.45	0.27	0.15	12.4	11000		0						Health	Health	
57	40 days	2022/1/17	16:50:59	40.76	1.84	3.55	5.22	11.32	908	0.58	0.08	0.08	10.8	21000		0						Health	Health	
58	40 days	2022/1/17	16:51:11	41.45	2.03	3.64	5.49	11.95	790.3	0.48	0.15	0.15	9.7	19000		0						Health	Health	
59	40 days	2022/1/17	16:51:23	40.93	1.65	3.68	5.64	11.86	808.6	0.43	0.12	0.13	8.8	9000		0						Health	Health	
60	40 days	2022/1/17	16:54:47	36.47	1.24	3.22	5.49	10.71	864.6	0.5	0.14	0.12	10.5	131000		65.3	Mild inflammation	++				subclinical mastitis	Subclinical mastitis	
61	40 days	2022/1/17	16:54:54	38.27	1.72	3.1	5.56	11.11	805.3	0.48	0.25	0.19	10.2	15000		0						Health	Health	
62	40 days	2022/1/17	16:55:05	38.11	1.23	3.26	5.49	10.75	855.4	0.48	0.25	0.18	13.2	9000		0						Health	Health	
63	40 days	2022/1/17	16:55:17	37.38	2.22	3.34	5.26	11.54	871.7	0.55	0.14	0.11	10.7	12000		0						Health	Health	
64	40 days	2022/1/17	16:55:29	37.41	0.89	3.38	5.51	10.55	816.1	0.46	0.23	0.13	12.4	9000		0						Health	Health	
65	40 days	2022/1/17	16:55:41	37.46	1.75	3.41	5.22	11.08	920.5	0.58	0.16	0.14	11.1	64000		62.1	Mild inflammation					Health	Suspected	
66	40 days	2022/1/17	16:55:53	37.76	0.86	3.29	5.38	10.25	927.1	0.53	0.21	0.14	13.2	10000		0						Health	Health	
67	40 days	2022/1/17	16:56:05	38.15	2.28	3.11	5.1	11.16	953.1	0.66	0.09	0.14	6.7	146000		66.4	Mild inflammation					Health	Suspected	
68	40 days	2022/1/17	16:56:16	38.66	1.18	3	5.25	10.08	931.2	0.57	0.21	0.16	10.3	15000		0						Health	Health	
69	40 days	2022/1/17	16:56:28	38.6	2.05	3.28	5.09	11.08	941.5	0.6	0.18	0.14	8.3	125000		62.5	Mild inflammation				+	Suspected	Suspected	
70	40 days	2022/1/17	16:56:40	38.39	1.78	2.73	5.19	10.28	972.1	0.6	0.14	0.11	9.8	204000	Mild inflammation	71.7	Mild inflammation				+	Suspected	Subclinical mastitis	
71	40 days	2022/1/17	16:56:52	37.99	1.9	3.23	5.26	10.97	914.4	0.58	0.19	0.17	10.2	142000		73.7	Mild inflammation	+				Suspected	Suspected	
72	40 days	2022/1/17	16:57:04	37.64	2.45	3.72	5.43	12.38	855.4	0.47	0.12	0.1	11.8	17000		0						Health	Health	
73	40 days	2022/1/17	16:57:16	37.31	1.61	3.44	5.29	11.18	875.5	0.54	0.16	0.11	13.1	39000		0						Health	Health	
74	40 days	2022/1/17	16:57:28	36.92	0.82	3.06	5.23	9.88	934.9	0.58	0.22	0.16	8.7	27000		0			+			Suspected	Suspected	
75	40 days	2022/1/17	16:57:40	36.67	0.77	3.26	5.58	10.38	827.7	0.47	0.3	0.19	13	17000		0						Health	Health	
76	40 days	2022/1/17	16:57:52	36.57	1.53	3.27	5.44	10.9	827.8	0.51	0.26	0.16	11.2	85000		65.7	Mild inflammation					Health	Suspected	
77	40 days	2022/1/17	16:58:04	37.15	1.54	3.63	5.1	11.1	960.1	0.58	0.15	0.11	9.9	20000		0						Health	Health	
78	40 days	2022/1/17	16:58:16	37.22	1.2	3.09	5.4	10.34	870.5	0.54	0.27	0.18	11.9	9000		0						Health	Health	
79	40 days	2022/1/17	16:58:28	37.32	1.06	3.27	5.28	10.34	922.8	0.57	0.17	0.13	8.1	26000		0						Health	Health	
80	40 days	2022/1/17	16:58:52	36.5	2.02	3.45	5.17	11.41	894.1	0.56	0.17	0.13	9.8	177000		87	Clinical mastitis					Health	Suspected	
81	40 days	2022/1/17	16:59:04	36.26	1.24	3.77	5.45	11.29	857.8	0.49	0.18	0.14	9	50000		0						Health	Health	
82	40 days	2022/1/17	16:59:16	36.28	1.56	3.5	5.31	11.07	868.4	0.55	0.16	0.12	11.2	26000		0			++		+	subclinical mastitis	Suspected	
83	40 days	2022/1/17	16:59:28	36.12	0.91	3.25	5.69	10.55	785.3	0.43	0.39	0.2	11.9	112000		81.4	Clinical mastitis					Health	Suspected	
84	40 days	2022/1/17	16:59:40	35.81	0.62	3.26	5.61	10.16	839	0.46	0.28	0.19	12.6	14000		0						Health	Health	
85	40 days	2022/1/17	16:59:52	37.4	0.96	3.65	5.51	11	822.6	0.45	0.22	0.13	10.5	16000		0			+			Suspected	Suspected	
86	40 days	2022/1/17	17:00:04	38.17	2.07	3.78	5.37	12.06	832.1	0.47	0.13	0.11	12.8	17000		0		+				Suspected	Suspected	
87	40 days	2022/1/17	17:00:16	38.64	1.12	3.47	5.18	10.49	960.7	0.57	0.19	0.15	10.3	495000	Mild inflammation	82.7	Clinical mastitis			++	+++	Clinical mastitis	Suspected clinical mastitis	
88	40 days	2022/1/17	17:00:28	38.99	1.4	3.05	4.89	10.03	991.2	0.68	0.14	0.13	9	974000	Clinical mastitis	75.1	Clinical mastitis	++	+++	++		Clinical mastitis	Clinical mastitis	
89	40 days	2022/1/17	17:00:40	38.23	1.45	3.56	5.47	11.35	811.7	0.48	0.14	0.14	12	15000		0						Health	Health	
90	40 days	2022/1/17	17:00:52	38.31	0.52	3.28	4.94	9.47	1046	0.65	0.35	0.18	9.4	190000		89.6	Clinical mastitis		+			Suspected	Suspected	
91	40 days	2022/1/17	17:01:04	37.86	0.88	3.45	5.36	10.46	890.7	0.53	0.26	0.16	11.5	8000		0						Health	Health	
92	40 days	2022/1/17	17:01:16	37.79	1.41	3.49	5.41	11.1	829.8	0.5	0.22	0.07	8.6	12000		0						Health	Health	
93	40 days	2022/1/17	17:01:28	37.32	2.81	3.99	5.42	13.07	819.4	0.47	0.08	0.1	12.3	89000		76.6	Clinical mastitis					Health	Suspected	
94	40 days	2022/1/17	17:01:40	37.65	0.41	3.89	5.29	10.56	923.2	0.48	0.47	0.21	13.2	20000		0						Health	Health	
95	40 days	2022/1/17	17:01:52	37.58	1.01	3.08	5.48	10.26	864.1	0.5	0.25	0.19	12.1	11000		0						Health	Health	
96	40 days	2022/1/17	17:02:04	38.67	0.89	3.04	5.33	9.93	983.5	0.55	0.18	0.16	9.8	44000		0						Health	Health	
97	40 days	2022/1/17	17:02:16	38.81	2.93	3.12	4.22	10.8	1213.7	0.93	0.17	0.27	10.9	111000		71.3	Mild inflammation	+	+			Suspected	Suspected	
98	40 days	2022/1/17	17:02:28	38.74	1.38	2.68	5.4	9.99	857.8	0.56	0.15	0.15	7.7	125000		81.7	Clinical mastitis					Health	Suspected	
99	40 days	2022/1/17	17:02:40	38.44	3.17	3.28	4.85	12	991.3	0.66	0.11	0.16	11.7	302000	Mild inflammation	82.6	Clinical mastitis			++	+++	Clinical mastitis	Suspected clinical mastitis	
100	40 days	2022/1/17	17:02:52	37.8	2.6	3.49	5.34	12.17	848.7	0.52	0.14	0.16	13.4	17000		0						Health	Health	
101	40 days	2022/1/17	17:03:04	37.56	0.84	3.08	5.43	10.03	903.3	0.52	0.21	0.15	9.4	13000		0						Health	Health	
102	40 days	2022/1/17	17:03:16	37.31	3.31	3.33	4.93	12.22	962.8	0.67	0.09	0.18	13.1	180000		47.2		+			+	Suspected	Suspected	
103	40 days	2022/1/17	17:03:28	37.16	0.96	3.47	5.35	10.56	890.1	0.52	0.21	0.13	13.2	32000		0						Health	Health	
104	40 days	2022/1/17	17:03:40	36.5	1	3.32	5.53	10.57	839.7	0.49	0.22	0.17	8.3	39000		0						Health	Health	
105	40 days	2022/1/17	17:03:52	36.55	1.68	3.33	5.56	11.35	793.5	0.47	0.18	0.14	12.1	23000		0						Health	Health	
106	40 days	2022/1/17	17:04:04	38.32	0.87	3.1	5.11	9.71	1005.4	0.62	0.31	0.17	12.7	107000		86.2	Clinical mastitis					Health	Suspected	
107	40 days	2022/1/17	17:04:16	38.5	1.05	3.54	5.48	10.82	848.9	0.48	0.25	0.14	11.3	26000		0						Health	Health	
108	40 days	2022/1/17	17:04:28	38.21	1.21	3.42	5.35	10.78	859.9	0.53	0.28	0.19	11.1	11000		0						Health	Health	
109	40 days	2022/1/17	17:04:40	37.92	1.25	3.34	5.23	10.56	941.6	0.57	0.13	0.11	11.5	8000		0						Health	Health	
110	40 days	2022/1/17	17:04:52	37.41	1.28	3.49	5.55	11.06	817.2	0.47	0.25	0.16	10	9000		0						Health	Health	
111	40 days	2022/1/17	17:05:16	36.97	0.94	3.44	5.6	10.69	842.8	0.49	0.3	0.17	6.9	19000		0						Health	Health	
112	40 days	2022/1/17	17:05:40	36.11	1.27	3.57	4.94	10.57	1001.9	0.63	0.2	0.14	10.1	136000		71	Mild inflammation				++	subclinical mastitis	Subclinical mastitis	
113	40 days	2022/1/17	17:05:52	37.71	1.03	3.48	4.64	9.8	1149.4	0.74	0.2	0.17	6.1	127000		64.9	Mild inflammation	+	++			subclinical mastitis	Subclinical mastitis	
114	40 days	2022/1/17	17:06:04	38.69	0.27	5.77	1.87	9.73	1880.3	1.33	0.27	0.17	-0.8	1214000	Clinical mastitis	80.3	Clinical mastitis	+++	++	+++		Clinical mastitis	Clinical mastitis	
115	40 days	2022/1/17	17:06:16	38.94	0.96	3.32	4.48	9.35	1189.5	0.8	0.36	0.27	7.7	199000		40.7				+	+	Health	Suspected	

				Milk quality analysis RAW data														CMT analysis RAW data					Result	Sample selection
No	postpartum	Date	Time	Temp	Fat	Prot.Cru.	Lactose	Total Solids	Conductivity	H.index	Acetone	BHB	BUN	SCC (cells/mL)	SCC Analysis	DSCC (%)	DSCC Analysis	Left Fore	Right Fore	Left Hind	Right Hind	CMT Analysis	Omprehensive analysis	
116	40 days	2022/1/17	17:06:40	38.48	0.56	3.32	5.09	9.66	973.6	0.58	0.35	0.13	10.4	331000	Mild inflammation	86.3	Clinical mastitis					Health	Suspected	
117	40 days	2022/1/17	17:06:52	38.28	0.63	3.36	5.3	10.03	923.8	0.54	0.2	0.14	12.2	46000		0						Health	Health	
118	40 days	2022/1/17	17:07:04	37.59	2.36	3.15	5.34	11.51	867.7	0.55	0.12	0.13	9.3	29000		0						Health	Health	
119	40 days	2022/1/17	17:07:16	37.84	1.26	3.26	5.51	10.74	788.5	0.5	0.21	0.16	9.8	10000		0						Health	Health	
120	40 days	2022/1/17	17:07:28	37.09	2.04	3.25	5.49	11.5	839.2	0.5	0.22	0.19	14.9	17000		0						Health	Health	
121	40 days	2022/1/17	17:07:40	36.8	1.49	3.37	5.46	11.02	795.6	0.5	0.23	0.16	9.6	10000		0					+	Suspected	Suspected	
122	40 days	2022/1/17	17:07:52	36.75	2.11	3.16	5.03	10.88	971	0.68	0.05	0.12	9.7	435000	Mild inflammation	87.2	Clinical mastitis		++	+++		Clinical mastitis	Suspected clinical mastitis	
123	40 days	2022/1/17	17:08:04	37.43	1.95	3.47	5.59	11.74	826	0.46	0.18	0.15	12.2	14000		0						Health	Health	
124	40 days	2022/1/17	17:08:16	37.82	0.62	2.95	5.44	9.7	872.6	0.51	0.24	0.15	9.1	24000		0						Health	Health	
125	100 days	2022/1/17	18:27:49	39.13	1.91	3.68	5.5	11.92	851.4	0.46	0.13	0.13	15.5	62000		47.3						Health	Health	
126	100 days	2022/1/17	18:28:01	40.34	2.4	3.28	5.51	11.93	793.7	0.48	0.12	0.19	9.7	88000		77.7	Clinical mastitis					Health	Suspected	
127	100 days	2022/1/17	18:28:13	40.44	2.71	3.41	5.36	12.2	867.4	0.54	0.03	0.11	12.1	437000	Mild inflammation	83.4	Clinical mastitis					Health	Suspected	
128	100 days	2022/1/17	18:28:25	40.61	1.41	3.36	5.57	11.09	860.1	0.47	0.33	0.22	14.4	15000		0						Health	Health	
129	100 days	2022/1/17	18:28:37	40.15	2.26	3.58	5.31	11.9	853.1	0.52	0.08	0.16	11.4	85000		69.4	Mild inflammation					Health	Suspected	
130	100 days	2022/1/17	18:28:49	40.26	2.04	3.3	5.5	11.52	794.3	0.48	0.15	0.15	11.9	130000		81.2	Clinical mastitis					Health	Suspected	
131	100 days	2022/1/17	18:29:01	39.67	2.12	3.43	5.15	11.49	895.1	0.6	0.08	0.11	9.6	60000		41.4						Health	Health	
132	100 days	2022/1/17	18:29:13	40.41	2.36	3.67	5.42	12.21	829	0.48	0.09	0.12	13.2	36000		0						Health	Health	
133	100 days	2022/1/17	18:29:25	40	2.43	3.52	5.17	11.82	918.8	0.59	0.1	0.16	14.9	167000		49.6			++			subclinical mastitis	Suspected	
134	100 days	2022/1/17	18:29:37	39.87	1.16	3.56	5.33	10.84	916.2	0.52	0.1	0.09	12.1	22000		0						Health	Health	
135	100 days	2022/1/17	18:29:49	39.38	1.35	3.59	5.36	11.1	852.6	0.51	0.14	0.11	12.7	28000		0						Health	Health	
136	100 days	2022/1/17	18:30:01	39.35	1.39	3.31	5.18	10.65	906.1	0.58	0.15	0.14	13.6	25000		0						Health	Health	
137	100 days	2022/1/17	18:30:13	39.65	2.68	4.03	5.53	13.13	764.4	0.41	0.09	0.12	14.6	27000		0						Health	Health	
138	100 days	2022/1/17	18:30:25	39.36	2.23	3.77	5.36	12.12	832.3	0.54	0.1	0.12	13.2	89000		75.3	Clinical mastitis		+++	++		Clinical mastitis	Suspected clinical mastitis	
139	100 days	2022/1/17	18:30:37	39.68	1.41	3.31	5.6	11.07	843.1	0.45	0.23	0.16	11.1	17000		0						Health	Health	
140	100 days	2022/1/17	18:31:42	37.28	2.83	3.78	5.31	12.72	847.3	0.54	0.1	0.12	10.7	155000		63.1	Mild inflammation					Health	Suspected	
141	100 days	2022/1/17	18:31:49	38.01	2.46	3.62	5.46	12.29	792.2	0.51	0	0.07	16.8	33000		0				+		Suspected	Suspected	
142	100 days	2022/1/17	18:32:01	37.89	1.78	3.46	5.22	11.19	883.1	0.55	0.06	0.09	14.4	74000		72.5	Mild inflammation				+	Suspected	Suspected	
143	100 days	2022/1/17	18:32:12	38.09	1.99	3.7	5.4	11.93	886.7	0.5	0.07	0.1	14.3	17000		0						Health	Health	
144	100 days	2022/1/17	18:32:24	37.73	1.5	3.52	5.21	11.04	904.3	0.54	0.17	0.13	14.9	115000		67	Mild inflammation	+	+	+	++	subclinical mastitis	subclinical mastitis	
145	100 days	2022/1/17	18:32:36	37.64	1.76	3.49	5.22	11.23	884.3	0.57	0.09	0.11	9.3	67000		64.3	Mild inflammation		+		+	Suspected	Suspected	
146	100 days	2022/1/17	18:32:48	39.72	1.37	3.36	5.17	10.64	953.2	0.58	0.15	0.12	12.7	287000	Mild inflammation	85	Clinical mastitis	+			+	Suspected	Suspected	
147	100 days	2022/1/17	18:33:00	39.78	3.58	4.29	5.12	13.98	842	0.49	0	0.07	14.3	30000		0						Health	Health	
148	100 days	2022/1/17	18:33:11	39.95	2.48	3.82	4.98	12.21	969.7	0.58	0.08	0.11	13.2	53000		48				+	+	Suspected	Suspected	
149	100 days	2022/1/17	18:33:23	39.81	3.02	4.09	5.24	13.24	809.4	0.53	0	0.06	13.3	111000		53.8				+	+	Suspected	Suspected	
150	100 days	2022/1/17	18:33:35	39.63	0.81	3.34	5.25	10.15	936.2	0.55	0.2	0.11	12.3	12000		0						Health	Health	
151	100 days	2022/1/17	18:33:47	39.33	1.74	3.87	4.69	11.15	1034.9	0.7	0.17	0.14	10.2	896000	Clinical mastitis	65	Mild inflammation	+++	++	+++	+	Clinical mastitis	Suspected clinical mastitis	
152	100 days	2022/1/17	18:33:59	39.14	1.81	3.57	4.7	10.73	1003.9	0.71	0.17	0.16	11.1	37000		0				++		subclinical mastitis	Suspected	
153	100 days	2022/1/17	18:34:11	39.17	1.74	3.5	5.42	11.44	836.5	0.48	0.14	0.12	15.4	15000		0						Health	Health	
154	100 days	2022/1/17	18:34:23	39.08	1.52	3.84	4.71	10.91	1099.9	0.65	0.19	0.19	13	525000	Clinical mastitis	60.6	Mild inflammation	+++	+	++	+++	Clinical mastitis	Suspected clinical mastitis	
155	100 days	2022/1/17	18:34:35	38.4	0.82	3.15	5.42	10.16	857.1	0.49	0.29	0.13	10.2	9000		0						Health	Health	
156	100 days	2022/1/17	18:34:47	38.91	2.26	3.38	5.48	11.91	806.5	0.53	0.1	0.16	11.6	52000		73.8	Mild inflammation	+				Suspected	Suspected	
157	100 days	2022/1/17	18:34:59	39.43	0.99	3.64	5.3	10.83	919.7	0.54	0.18	0.14	10.6	14000		0		+	+	+	++	subclinical mastitis	Suspected	
158	100 days	2022/1/17	18:35:11	39.22	2.15	3.57	5.41	11.84	830.2	0.52	0.1	0.17	14.6	63000		62.1	Mild inflammation			+	++	subclinical mastitis	Subclinical mastitis	
159	100 days	2022/1/17	18:35:23	39.31	0.42	3.89	5.3	10.42	939.4	0.5	0.24	0.13	14.7	49000		0				++		subclinical mastitis	Suspected	
160	100 days	2022/1/17	18:35:35	39.13	1.43	3.64	5.41	11.23	853.6	0.51	0.14	0.12	10.9	14000		0						Health	Health	
161	100 days	2022/1/17	18:35:47	38.27	4.48	3.02	4.73	12.72	960.1	0.69	0.11	0.17	15.9	54000		61	Mild inflammation		+			Suspected	Suspected	
162	100 days	2022/1/17	18:35:59	38.54	2.01	3.66	5.14	11.48	900.1	0.58	0.1	0.1	11.7	53000		67.7	Mild inflammation	+	++	++	+	subclinical mastitis	Subclinical mastitis	
163	100 days	2022/1/17	18:36:11	38.74	1.03	3.45	5.6	10.83	837	0.45	0.2	0.14	12.6	8000		0						Health	Health	
164	100 days	2022/1/17	18:36:23	38.52	0.91	3.44	5.3	10.37	924.3	0.52	0.17	0.11	15.4	10000		0						Health	Health	
165	100 days	2022/1/17	18:36:35	37.47	1.79	3.75	5.16	11.53	920.8	0.55	0.12	0.12	12.6	110000		63.6	Mild inflammation	++	+	+		subclinical mastitis	Subclinical mastitis	
166	100 days	2022/1/17	18:36:47	39.42	1.07	3.44	5.43	10.7	860.7	0.5	0.17	0.1	12.2	9000		0						Health	Health	
167	100 days	2022/1/17	18:36:59	39.68	1.88	3.71	5.51	11.86	825.8	0.47	0.13	0.11	13.6	53000		75.5	Clinical mastitis		+	+++	+	Clinical mastitis	Suspected clinical mastitis	
168	100 days	2022/1/17	18:37:11	39.64	1.42	4.05	5.34	11.72	861.7	0.47	0.14	0.08	14.1	24000		0						Health	Health	
169	100 days	2022/1/17	18:37:23	39.83	1.18	3.6	5.46	11.04	801.5	0.49	0.22	0.13	14.3	25000		0						Health	Health	
170	100 days	2022/1/17	18:37:35	39.41	2.18	3.6	4.39	10.83	1150	0.8	0.11	0.14	9.7	347000	Mild inflammation	49.6		++	+	+++	+++	Clinical mastitis	Suspected clinical mastitis	
171	100 days	2022/1/17	18:37:47	39.31	1.63	3.45	5.36	11.19	859	0.52	0.13	0.13	13.3	9000		0						Health	Health	
172	100 days	2022/1/17	18:37:59	39.31	2	3.44	5.21	11.43	915.2	0.58	0.08	0.13	12.1	46000		0				+	+	Suspected	Suspected	
173	100 days	2022/1/17	18:38:11	39.21	0.9	3.23	5.38	10.25	893.7	0.51	0.28	0.17	16.8	18000		0						Health	Health	
174	100 days	2022/1/17	18:38:23	39.07	1.31	3.75	5	10.85	967.9	0.62	0.17	0.12	9.7	481000	Mild inflammation	71.9	Mild inflammation	++			+++	Clinical mastitis	subclinical mastitis	
175	100 days	2022/1/17	18:38:35	38.63	1.62	3.64	5.08	11.17	999.7	0.57	0.09	0.1	11.5	202000	Mild inflammation	52.7			+	+++	++	Clinical mastitis	Suspected clinical mastitis	
176	100 days	2022/1/17	18:38:47	39.38	1.67	3.2	4.85	10.3	1044.5	0.72	0.07	0.1	12.7	73000		39.7				+	+	Suspected	Suspected	
177	100 days	2022/1/17	18:38:59	39.47	0.93	3.16	5.39	10.24	924.8	0.54	0.23	0.16	11.1	15000		0				+	+	Suspected	Suspected	

				Milk quality analysis RAW data														CMT analysis RAW data					Result	Sample selection
No	postpartum	Date	Time	Temp	Fat	Prot.Cru.	Lactose	Total Solids	Conductivity	H.index	Acetone	BHB	BUN	SCC (cells/mL)	SCC Analysis	DSCC (%)	DSCC Analysis	Left Fore	Right Fore	Left Hind	Right Hind	CMT Analysis	Omprehensive analysis	
178	100 days	2022/1/17	18:39:11	39.66	1.72	3.29	5.46	11.1	794.7	0.51	0.16	0.13	14.7	107000		67	Mild inflammation		+	+	++	subclinical mastitis	Subclinical mastitis	
179	100 days	2022/1/17	18:39:23	39.41	1.48	3.98	5.59	11.91	780	0.4	0.27	0.16	13.4	34000		0						Health	Health	
180	100 days	2022/1/17	18:39:35	39.5	0.8	3.26	5.39	10.11	902	0.52	0.22	0.15	14.2	27000		0						Health	Health	
181	100 days	2022/1/17	18:39:47	39.11	4	3.72	4.58	13	957.3	0.69	0.06	0.14	15.1	224000	Mild inflammation	59		+	+	+	+	Suspected	Suspected	
182	100 days	2022/1/17	18:39:59	38.89	1.5	3.89	5.1	11.41	921.2	0.56	0.1	0.09	13.4	18000		0						Health	Health	
183	100 days	2022/1/17	18:40:11	39.05	1.2	3.84	5.19	11.03	944.2	0.52	0.16	0.09	13.1	14000		0						Health	Health	
184	100 days	2022/1/17	18:40:23	38.98	0.75	3.29	5.23	10.04	919.2	0.57	0.22	0.15	8.7	16000		0						Health	Health	
185	100 days	2022/1/17	18:40:35	38.45	1.47	3.83	5.32	11.55	814.3	0.5	0.19	0.12	10.4	77000		74.1	Mild inflammation			++		subclinical mastitis	Subclinical mastitis	
186	100 days	2022/1/17	18:40:47	40.05	1.61	3.37	5.15	10.77	907.6	0.61	0.11	0.13	11.5	93000		43		+	+	+	+	Suspected	Suspected	
187	100 days	2022/1/17	18:40:59	40.28	3	3.74	5.24	12.71	893.3	0.53	0.09	0.11	19.4	22000		0			+		+	Suspected	Suspected	
188	100 days	2022/1/17	18:41:11	40.38	2.82	4.21	5.14	13.21	838.7	0.52	-0.01	0.05	12.5	28000		0						Health	Health	
189	100 days	2022/1/17	18:41:23	40.29	1.49	3.74	5.15	11.24	931.7	0.56	0.09	0.08	11.8	21000		0						Health	Health	
190	100 days	2022/1/17	18:41:35	40.4	1.6	3.66	5.42	11.44	824.4	0.49	0.09	0.1	13.2	20000		0						Health	Health	
191	100 days	2022/1/17	18:41:47	39.81	3.27	3.74	4.95	12.69	887.4	0.65	0.01	0.07	13.1	180000		71	Mild inflammation			+++	++	Clinical mastitis	Suspected clinical mastitis	
192	100 days	2022/1/17	18:41:59	39.97	2.09	3.68	5.37	11.97	836.3	0.49	0.09	0.12	16.3	74000		71.5	Mild inflammation			++		subclinical mastitis	Subclinical mastitis	
193	100 days	2022/1/17	18:42:11	39.49	1.25	3.37	5.45	10.77	846.9	0.5	0.12	0.11	12.8	17000		0						Health	Health	
194	100 days	2022/1/17	18:42:23	39.73	1.84	3.39	4.86	10.77	989.6	0.68	0.05	0.08	12.4	9000		0						Health	Health	
195	100 days	2022/1/17	18:42:35	38.98	2.47	3.78	5.52	12.56	785.6	0.46	0.08	0.09	15.3	15000		0						Health	Health	
196	100 days	2022/1/17	18:42:47	39.19	1.52	3.78	5.28	11.46	851	0.53	0.13	0.09	13.4	10000		0						Health	Health	
197	100 days	2022/1/17	18:42:59	39.53	0.91	3.82	5.29	10.97	866.8	0.49	0.25	0.14	12.5	19000		0						Health	Health	
198	100 days	2022/1/17	18:43:11	39.21	1.79	3.27	5.21	10.95	932.1	0.59	0.1	0.13	11.6	35000		0					++	subclinical mastitis	Suspected	
199	100 days	2022/1/17	18:43:23	39.19	1.02	3.4	4.92	10.05	1017.5	0.62	0.23	0.13	12.1	32000		0				+		Suspected	Suspected	
200	100 days	2022/1/17	18:43:35	38.97	0.89	3.69	5.37	10.84	818.7	0.49	0.22	0.12	10.3	46000		0						Health	Health	
201	100 days	2022/1/17	18:43:47	38.72	1.91	4.03	5.28	12.16	837.8	0.5	0.06	0.08	13.4	30000		0						Health	Health	
202	100 days	2022/1/17	18:43:59	38.4	2.02	3.89	5.34	12.18	814.7	0.47	0.11	0.12	15.4	53000		74.9	Mild inflammation				+	Suspected	Suspected	
203	100 days	2022/1/17	18:44:11	38.4	3.93	3.47	4.92	13.07	911.6	0.57	0.05	0.13	12.9	165000		66	Mild inflammation			+	+	Suspected	Suspected	
204	100 days	2022/1/17	18:44:23	38.37	1.41	3.56	5.71	11.4	774.6	0.42	0.19	0.15	11.2	6000		0						Health	Health	
205	100 days	2022/1/17	18:44:35	37.74	0.81	3.7	5.4	10.74	837.6	0.48	0.3	0.18	9.8	68000		83.7	Clinical mastitis			+++	++	Clinical mastitis	Suspected clinical mastitis	
206	100 days	2022/1/17	18:44:47	39.33	0.74	3.42	5.21	10.06	958.2	0.57	0.19	0.12	12.7	18000		0						Health	Health	
207	100 days	2022/1/17	18:44:59	39.31	0.63	3.38	5.2	9.96	938.6	0.56	0.28	0.13	9.9	7000		0						Health	Health	
208	100 days	2022/1/17	18:45:11	39.45	1.61	3.53	5.04	10.94	958.7	0.62	0.13	0.1	15.8	104000		65.7	Mild inflammation		+	++	++	subclinical mastitis	Subclinical mastitis	
209	100 days	2022/1/17	18:45:23	39.15	1.05	3.49	5.53	10.75	859	0.47	0.26	0.17	14.3	78000		73.8	Mild inflammation	+	+	++	+	subclinical mastitis	Subclinical mastitis	
210	100 days	2022/1/17	18:45:35	39.61	1.04	3.06	5.48	10.21	830.8	0.51	0.19	0.13	13.7	6000		0						Health	Health	
211	100 days	2022/1/17	18:45:47	39.03	1.84	4.17	5.28	12.23	827.1	0.49	0.06	0.03	11.7	36000		0						Health	Health	
212	100 days	2022/1/17	18:45:59	38.8	1.12	3.64	5.12	10.6	969.6	0.58	0.17	0.1	12.1	14000		0						Health	Health	
213	100 days	2022/1/17	18:46:11	38.79	1.35	3.41	5.01	10.51	986.9	0.63	0.15	0.11	11	27000		0						Health	Health	
214	100 days	2022/1/17	18:46:23	38.13	1.51	3.22	4.57	9.86	1156.2	0.8	0.1	0.14	7.7	64000		41.6				++	++	subclinical mastitis	Suspected	
215	100 days	2022/1/17	18:46:35	37.95	0.93	3.36	5.48	10.46	858.9	0.49	0.16	0.13	12.9	6000		0						Health	Health	
216	100 days	2022/1/17	18:46:47	39.4	4.64	4.68	4.93	15.46	798.1	0.51	0.03	0.12	13.7	293000	Mild inflammation	76	Clinical mastitis	++	+	++	+++	Clinical mastitis	Suspected clinical mastitis	

Table S3 The used antibodies and dilution ratio in the present study.

NO.	Antibody	Full Name	Company	Cat No	Host / Isotype	Molecular weight	Application	Dilution
1	IgG(Alexa Fluor® 488)	Goat Anti-Rabbit IgG H&L (Alexa Fluor® 488)	Abcam, Cambridge, UK	ab150077	Goat/ IgG	——	IF	1:350
2	IgG(Alexa Fluor® 647)	Goat Anti-Rabbit IgG H&L (Alexa Fluor® 647)	Abcam, Cambridge, UK	ab150079	Goat/ IgG	——	IF	1:350
3	IgG(Alexa Fluor® 594)	Goat Anti-Rabbit IgG H&L (Alexa Fluor® 594)	Abcam, Cambridge, UK	ab150080	Goat/ IgG	——	IF	1:350
4	CK-18	Cytokeratin 18	Servicebio, Wuhan, China	GB11232	Rabbit / IgG	——	IF	1:300
5	NLRP3	NLR family pyrin domain containing 3	Proteintech, Wuhan, China	19771-1-AP	Rabbit / IgG	110 kDa	IHC<br>IF<br>WB	1:100<br>1:100<br>1:500
6	CASP1/P20	Caspase-1/P20	Proteintech, Wuhan, China	22915-1-AP	Rabbit / IgG	CASP1 45 kDa<br>CASP1 p20 20 kDa	IHC<br>IF<br>WB	1:200<br>1:100<br>1:4000
7	GSDMD	Gasdermin-D	Proteintech, Wuhan, China	20770-1-AP	Rabbit / IgG	GSDMD 50 kDa<br>GSDMD-N 30 kDa	IHC<br>IF<br>WB	1:200<br>1:200<br>1:5000
8	IL-1β	Interleukin-1-beta	Proteintech, Wuhan, China	16806-1-AP	Rabbit / IgG	32 kDa	WB	1:4000
9	IL-18	Interleukin-18	Proteintech, Wuhan, China	10663-1-AP	Rabbit / IgG	22 kDa	WB	1:6000
10	β-actin	Beta Actin	Bioss, Beijing, China	bs-0061R	Rabbit / IgG	42 kDa	WB	1:4000
11	IgG	Goat Anti-Rabbit IgG H&L/HRP	Bioss, Beijing, China	bs-80295G-HRP	Rabbit / IgG	——	WB	1:5000

Table S4 qRT-PCR primer sequences.

Species	Gene	Primer sequence(5'-3')	Gene entry number	Product length
Bos	*β-actin*	F: CCAAGGCCAACCGTGAGAA<br>R: CCAGAGGCATACAGGGACAG	NM_173979.3	102 bp
Bos	*NLRP3*	F: ACCACAACTCTGCTACCCTC<br>R: TCTCGCAGTCCACTTCCTTT	NM_001102219.1	168 bp
Bos	*CASP1*	F: TGCATCTTCAGGACCAGGAG<br>R: ATCAGCTCCGTCTCTTCTGG	XM_024975697.1	178 bp
Bos	*GSDMD*	F: TTTGTAGTGACCGAGGTGCT<br>R: TGGTGACCGTCTTCTTCTGG	NM_001046160.3	151 bp
Bos	*IL-1β*	F: TCAATAAAGTGCAAACTCCAGGACA<br>R: CTTGCACAAAGCTCATGCAGAA	NM_174093.1	133 bp
Bos	*IL-18*	F: TGGCAAACTTGAACCTAAGCT<br>R: CTGCACAGAGATGGTTACGG	NM_174091.2	196 bp
Bos	*TLR4*	F: CATCATCTTCATCGTCCTG<br>R: ATCTGCTGTTCCTTCTGG	NM_174198.6	190 bp
Bos	*TNF -α*	F: AAGCCTCAAGTAACAAGCCGGTAG<br>R: TCACACCGTTGGCCATGAG	NM_173966.3	108 bp
Bos	*IL-6*	F: CCTTCACTCCATTCGCTGTCT<br>R: TCCTGATTTCCCTCATACTCG	NM_173923.2	191 bp
Mus	*β-actin*	F: AGGGAAATCGTGCGTGACAT<br>R: GCTGGAAAAGAGCCTCAGGG	NM_007393.5	175 bp
Mus	*NLRP3*	F: AAGCAACAGATGGAGACCGG<br>R: CAAATTCCATCCGCAGCCAG	NM_145827.4	167 bp
Mus	*CASP1*	F: CTGAGGGCAAAGAGGAAGCA<br>R: GATCACATAGGTCCCGTGCC	NM_009807.2	163 bp
Mus	*GSDMD*	F: ACAGTTCCAGTGCCTCCATG<br>R: ACCTCGGTCACCACAAACAG	NM_026960.4	154 bp
Mus	*IL1β*	F: TGCCACCTTTTGACAGTGATG<br>R: ATGTGCTGCTGCGAGATTTG	NM_008361.4	136 bp
Mus	*IL-18*	F: ACTTTGGCCGACTTCACTGT<br>R: TTCACAGAGAGGGTCACAGC	NM_008360.2	196 bp

Table S5 The 17 significant biological processes (BPs) and 276 DEPs identified from DIA proteomics.

NO.	Classification	GO ID	Description	Down DEPs	Up DEPs	Con-vs-CM	All	pvalue	p.adjust	Genes
1	Cytokine production	GO:0001816	cytokine production	38	147	185	508	1.69E-05	3.60E-04	NP 001007817.1(FADD);NP 001011676.2(CD
2	Cytokine production	GO:0001817	regulation of cytokine production	33	135	168	441	2.06E-06	6.07E-05	NP 001007817.1(FADD);NP 001011676.2(CD
3	Cytokine production	GO:0001819	positive regulation of cytokine production	20	80	100	271	8.31E-04	8.97E-03	NP 001007817.1(FADD);NP 001011676.2(CD
4	Cytokine production	GO:0032612	interleukin-1 production	0	19	19	34	5.81E-04	6.64E-03	NP 001077180.1(TNFAIP8);NP 001095689.1(
5	Cytokine production	GO:0032652	regulation of interleukin-1 production	0	16	16	28	1.14E-03	1.15E-02	NP 001095689.1(NLRP3);NP 001179099.1(Tn
6	Cytokine production	GO:0032651	regulation of interleukin-1 beta production	0	13	13	24	6.14E-03	4.32E-02	NP 001095689.1(NLRP3);NP 001179099.1(Tn
7	Cytokine production	GO:0032731	positive regulation of interleukin-1 beta producti	0	9	9	14	5.02E-03	3.89E-02	NP 001095689.1(NLRP3);NP 777155.1(PYCA
8	Cytokine production	GO:0032732	positive regulation of interleukin-1 production	0	9	9	14	5.02E-03	3.89E-02	NP 001095689.1(NLRP3);NP 777155.1(PYCA
9	Immune and defense	GO:0002376	immune system process	185	482	667	1745	3.06E-23	1.06E-19	NP 001007817.1(FADD);NP 001012692.2(HL
10	Immune and defense	GO:0006952	defense response	71	276	347	886	8.48E-14	2.46E-11	NP 001007817.1(FADD);NP 001014908.1(TR
11	Immune and defense	GO:0002252	immune effector process	43	147	190	433	5.64E-13	1.51E-10	NP 001007817.1(FADD);NP 001012692.2(HL
12	Immune and defense	GO:0031347	regulation of defense response	18	153	171	423	1.63E-08	1.09E-06	NP 001014908.1(TRAFD1);NP 001015528.1(
13	Immune and defense	GO:0031348	negative regulation of defense response	3	24	27	61	4.75E-03	3.73E-02	NP 001014908.1(TRAFD1);NP 001015528.1(
14	Inflammatory response	GO:0006954	inflammatory response	9	39	48	108	1.83E-04	2.67E-03	NP 001019727.2(VNN1);NP 001033253.1(PR
15	Inflammatory response	GO:0002526	acute inflammatory response	4	27	31	55	9.24E-06	2.22E-04	NP 001033253.1(PRCP);NP 001039444.1(C6);
16	Inflammatory response	GO:0050727	regulation of inflammatory response	3	13	16	27	6.58E-04	7.32E-03	NP 001039444.1(C6);NP 001095336.1(IDO1);
17	Inflammatory response	GO:0002673	regulation of acute inflammatory response	3	10	13	17	4.63649E-05	0.000822031	NP 001039444.1(C6);NP 001095689.1(NLRP3

NO.	ID	Symbol	Description	Con1	Con2	Con3	CM1	CM2	CM3	log2(fc)	PValue	FDR
1	XP 005220161.3	ABR	PREDICTED: active breakpoint cluster region-re	279748	235274.14	324221.125	1974531	2551850	3129168.5	3.189344998	4.39862E-13	3.1E-11
2	XP 005213930.2	ACOD1	PREDICTED: cis-aconitate decarboxylase [Bos i	0.001	0.001	0.001	123591	153203	182815	27.19086926	2.50E-10	9.43E-09
3	NP 001028790.1	ACTG1	PREDICTED: actin, cytoplasmic 2 isoform X1 [	423817472	393034208	454600736	1.34E+09	1521549312	1708007552	1.844026121	7.61E-06	8.57E-05
4	NP 001095683.1	ACTR2	actin-related protein 2 isoform b [Homo sapiens]	3937580	4079338.8	3795821	7019903	7069040	7118174	0.844204863	8.12E-03	2.87E-02
5	NP 776409.1	AHSG	alpha-2-HS-glycoprotein precursor [Bos taurus]	16808216	24441378	9175054	9287462	10115191	10942920	-0.732643045	1.04458E-06	1.56E-05
6	NP 776410.1	AIF1	allograft inflammatory factor 1 [Bos taurus]	1148880	1252458.9	1045300.438	16867188	17961888	19056588	3.966639267	8.98E-03	3.10E-02
7	NP 776926.1	ALOX15	arachidonate 15-lipoxygenase [Bos taurus]	4904760	5899319	3910196.75	111488	83004.3	54520.50781	-5.884852548	1.22E-61	2.32E-58
8	NP 001192632.1	ALOX15B	arachidonate 15-lipoxygenase B [Bos taurus]	117611	132451.7	102769.7188	1929421	1650640	1371859.75	3.810933184	3.45E-12	2.02E-10
9	NP 786978.2	ANXA1	annexin A1 [Bos taurus]	32484317	35426960	29541674	1.05E+08	121668188	137989168	1.905136726	3.87E-05	3.38E-04
10	XP 005208208.1	ANXA3	PREDICTED: annexin A3 isoform X1 [Bos taur	4653170	4928358	4377984.5	31508304	33493932	35479560	2.847613691	6.63E-10	2.24E-08
11	NP 001070578.1	AP1S1	PREDICTED: AP-1 complex subunit sigma-1A [	437225	452039.38	422409.9688	1050757	1059790	1068815.375	1.277327995	4.05E-03	1.64E-02
12	NP 001157408.1	APOBEC3G	probable DNA dC->dU-editing enzyme APOBE	0.001	0.001	0.001	234721.6	354364	474005.5938	28.40065566	1.08E-04	7.71E-04
13	NP 001192581.1	ARFGEF2	brefeldin A-inhibited guanine nucleotide-exchan	1226690	1223188.5	1230190.375	441848.2	562845	683842.625	-1.123959963	3.08E-05	2.80E-04
14	NP 001092351.2	ARHGEF2	rho guanine nucleotide exchange factor 2 [Bos ta	190901	152249.94	229551.375	798546.7	993158	1187770	2.379200657	1.30E-06	1.89E-05
15	NP 001179271.1	ARL11	ADP-ribosylation factor-like protein 11 [Bos tau	0.001	0.001	0.001	155903.2	219819	283734.9375	27.71174123	8.18E-05	6.06E-04
16	NP 001192206.1	ARRB2	beta-arrestin-2 [Bos taurus]	224185	255457.94	192912.4531	839086.3	839308	839530.0625	1.904509921	2.36E-04	1.50E-03
17	NP 001091491.1	ATP6AP2	renin receptor precursor [Bos taurus]	215954	215808.09	216100.7813	409419.2	414771	420122.1563	0.941588243	2.28E-04	1.45E-03
18	NP 787010.1	ATPIF1	ATPase inhibitor, mitochondrial precursor [Bos t	0.001	0.001	0.001	1471147	2017010	2562872.25	30.90957091	5.64E-05	4.51E-04
19	NP 001029503.1	AZGP1	zinc-alpha-2-glycoprotein precursor [Bos taurus]	3947840	5332088	2563600.5	2020221	1437090	853955.5625	-1.457915458	3.53E-05	3.13E-04
20	NP 803478.1	B4GALT1	beta-1,4-galactosyltransferase 1 [Bos taurus]	6534690	7227829	5841551.5	1205939	1069146	932353	-2.611659957	3.75E-10	1.36E-08
21	NP 001071386.1	BAK1	bcl-2 homologous antagonist/killer [Bos taurus]	1053670	1063828.3	1043511.813	2205651	2579270	2952898.25	1.291541448	1.69E-04	1.13E-03
22	XP 002688165.2	BANK1	PREDICTED: B-cell scaffold protein with ankyr	71073	66809.695	75336.21875	526608.3	659022	791436.5625	3.2129543	7.75E-07	1.21E-05
23	NP 776319.1	BAX	apoptosis regulator BAX [Bos taurus]	246046	194544.41	297548.375	2470101	3381410	4292726	3.780624421	8.03E-03	2.84E-02
24	NP 001028799.1	BECN1	beclin-1 [Bos taurus]	145573	137310.53	153835.2969	447650.6	451768	455885.125	1.633839588	4.64E-03	1.82E-02
25	NP 001069793.1	BLOC1S6	biogenesis of lysosome-related organelles compl	903814	978241.38	829385.6875	2205318	2455580	2705839.5	1.441966024	8.47E-03	2.96E-02
26	NP 001012694.2	BoLA-DQB	major histocompatibility complex, class II, DQ b	561428	666213	456643.75	7328165	8591311	9854457	3.935704733	7.76E-07	1.21E-05
27	NP 776320.1	BPI	bactericidal permeability-increasing protein prec	0.001	0.001	0.001	107404.1	120660	133916.1719	26.84637346	1.17E-05	1.24E-04
28	XP 024850604.1	BRD4	PREDICTED: LOW QUALITY PROTEIN: bror	311026	337760.84	284292.0938	1456244	1628090	1799941	2.388073389	1.46E-04	9.99E-04
29	XP 005207185.1	C1R	PREDICTED: complement C1r subcomponent is	599655	763367.94	435941.5	1117391	972059	826726.5625	0.696911343	5.60E-03	2.12E-02
30	NP 001070018.1	C1S	complement C1s subcomponent precursor [Bos t	401839	458540.66	345136.875	878137.4	792337	706537.3125	0.979497562	8.67E-04	4.56E-03
31	NP 001077221.1	C3AR1	C3a anaphylatoxin chemotactic receptor [Bos tau	0.001	0.001	0.001	268183.5	271751	275318	28.01770916	1.52E-07	3.05E-06
32	NP 001159957.1	C4A	complement C4 precursor [Bos taurus]	5815220	7843474	3786956.5	2267495	2306230	2344975	-1.334294606	2.60E-16	2.64E-14

33	NP 776677.1	C4BPA	PREDICTED: C4b-binding protein alpha chain i:	3158580	3476494.8	2840674.75	1815049	1803960	1792869.25	-0.80811071	1.97E-04	1.28E-03
34	NP 001039444.1	C6	complement component C6 precursor [Bos tauru:	2772178	3473222.5	2071133.5	1386062	1351210	1316352.375	-1.036770165	2.19E-06	3.03E-05
35	NP 001039750.1	C8A	complement component C8 alpha chain precurso	1740230	2262942.5	1217515.875	1041774	1050550	1059326.375	-0.728132661	2.29E-06	3.14E-05
36	NP 001033647.1	Cadm1	cell adhesion molecule 1 precursor [Bos taurus]	1919120	2000767.3	1837465.5	912288.3	871397	830505.3125	-1.139041263	6.03E-04	3.39E-03
37	NP 001076949.1	CAPZA1	F-actin-capping protein subunit alpha-1 [Bos tau	22734248	23670092	21798404	47070832	54342292	61613752	1.257208132	1.10E-12	7.24E-11
38	NP 001103266.1	CARD11	PREDICTED: caspase recruitment domain-conta	312418	11855.802	612980.625	121875.8	160326	198777.0313	-0.962467461	7.17E-04	3.94E-03
39	NP 001070579.1	CARD9	caspase recruitment domain-containing protein 9	54560.7	66155.063	42966.25781	628866.1	791944	955021.5625	3.859464872	4.34E-07	7.41E-06
40	NP 788811.1	CASP13	caspase-4 [Bos taurus]	192519	189969.3	195068.0469	1254216	1444160	1634098.625	2.907157615	6.86E-03	2.49E-02
41	NP 776429.1	CAV1	caveolin-1 [Bos taurus]	9145590	7881187	10410002	1490567	1126710	762855.5625	-3.020959444	2.30E-04	1.47E-03
42	XP 005218775.1	Cbfb	PREDICTED: core-binding factor subunit beta is	166054	144815.84	187292.8125	345646.9	342526	339404.0938	1.044557722	1.09E-02	3.62E-02
43	NP 001040050.1	CCL14	C-C motif chemokine 14 precursor [Bos taurus]	12032.7	11765.834	12299.61719	0.001	0.001	0.001	-23.52045911	6.17E-07	9.96E-06
44	NP 776433.1	CD14	monocyte differentiation antigen CD14 precursor	1831820	2078287.8	1585345.625	4873842	5512970	6152095	1.589553438	4.16E-03	1.67E-02
45	NP 001030424.1	CD151	PREDICTED: CD151 antigen isoform X1 [Bos t	795448	829555.38	761339.625	214886.7	159858	104829.7109	-2.314974827	4.21E-04	2.49E-03
46	XP 010803837.1	CD163	PREDICTED: scavenger receptor cysteine-rich t	144171	123176.52	165165.3594	3774836	4680610	5586390.5	5.020844745	7.93E-17	8.39E-15
47	NP 001011676.2	CD2	T-cell surface antigen CD2 precursor [Bos taurus	31761.5	30362.945	33159.98047	529587	604565	679542.625	4.250547332	9.29E-04	4.83E-03
48	NP 776437.1	CD247	T-cell surface glycoprotein CD3 zeta chain precu	16529.8	17597.08	15462.50879	220321	322022	423723.8438	4.284019711	1.49E-05	1.52E-04
49	NP 001156884.1	CD274	programmed cell death 1 ligand 1 precursor [Bos	0.001	0.001	0.001	330173.5	445069	559965	28.72945432	2.13E-07	4.10E-06
50	NP 001193468.1	CD2AP	CD2-associated protein [Bos taurus]	1628380	1759711	1497048.25	1089015	1043440	997855.3125	-0.642093948	7.88E-05	5.89E-04
51	NP 776436.1	CD3E	T-cell surface glycoprotein CD3 epsilon chain pr	47296.2	27549.686	67042.8125	447920	492878	537836.625	3.381434019	8.44E-03	2.96E-02
52	NP 001096695.1	CD4	T-cell surface glycoprotein CD4 precursor [Bos t	79424.3	9942.2861	148906.3906	289376.7	406124	522871.5	2.354267631	6.14E-03	2.29E-02
53	NP 001099081.1	CD40	tumor necrosis factor receptor superfamily memb	14635	20255.555	9014.482422	276257.3	340402	404546.375	4.539743202	5.09E-03	1.97E-02
54	NP 777133.2	CD47	leukocyte surface antigen CD47 precursor [Bos t	671490	604777.06	738203.625	1083222	2246800	3410384.75	1.742434787	6.80E-03	2.47E-02
55	NP 001039467.1	CD48	CD48 antigen precursor [Bos taurus]	196683	264939.78	128426.9922	1598507	2013620	2428732.5	3.35584518	5.52E-04	3.15E-03
56	NP 776324.1	CD5	T-cell surface glycoprotein CD5 precursor [Bos t	60735.4	66658.109	54812.71484	781200.4	931830	1082458.75	3.939456585	1.59E-04	1.08E-03
57	XP 002686171.3	CD58	PREDICTED: lymphocyte function-associated ar	919602	881653.81	957549.4375	1568054	1708190	1848335.75	0.893390081	1.16E-05	1.23E-04
58	NP 001029907.1	CD74	HLA class II histocompatibility antigen gamma c	559659	550727.06	568591.875	4596280	4908584	5220888.5	3.132686132	4.04E-04	2.42E-03
59	NP 001035616.1	CFB	complement factor B precursor [Bos taurus]	9337400	12762401	5912401.5	3349712	3349770	3349831.5	-1.478958472	2.49E-10	9.43E-09
60	XP 024831741.1	CFH	complement factor H precursor [Bos taurus]	6914930	8965405	4864447.5	3315080	3940240	4565405	-0.811429978	8.46E-08	1.79E-06
61	NP 783630.2	CGN1	conglutinin precursor [Bos taurus]	449713	550044.63	349381.2813	0.001	0.001	0.001	-28.74442925	3.57E-20	6.63E-18
62	NP 001068901.1	CIB1	calcium and integrin-binding protein 1 [Bos taur	322884	364347.06	281420.9375	129650.9	64825.5	0.001	-2.316383075	6.83E-03	2.48E-02
63	NP 001076918.1	CIDEA	cell death activator CIDE-A [Bos taurus]	831011	1063380	598642.875	0.001	242829	485657.3438	-1.774928651	6.09E-04	3.42E-03
64	XP 002691277.2	Clec4f	PREDICTED: C-type lectin domain family 4 me	0.001	0.001	0.001	213507.1	377891	542275.25	28.49339536	2.02E-07	3.91E-06
65	NP 001029651.1	CLEC6A	C-type lectin domain family 6 member A [Bos ta	0.001	0.001	0.001	99772.87	141272	182771.5781	27.07390183	8.76E-05	6.45E-04
66	NP 001092869.1	CMTM3	PREDICTED: CKLF-like MARVEL transmemb	2.23776	3.0331051	1.442424178	1288603	1504820	1721045.125	19.3591047	4.06E-04	2.42E-03
67	NP 001070299.1	COL3A1	collagen alpha-1(III) chain precursor [Bos taurus	33767545	40328032	27207058	7911410	6628720	5346027.5	-2.348835257	6.90E-06	7.93E-05
68	NP 001095313.1	COLEC12	collectin-12 [Bos taurus]	169431	208769.73	130091.3828	20256.72	14667.1	9077.464844	-3.530040278	7.07E-03	2.55E-02
69	NP 001020520.1	CRIP1	cysteine-rich protein 1 [Bos taurus]	4256500	3181129.8	5331875	9051722	10245900	11440155	1.267310258	1.45E-13	1.10E-11
70	NP 001137569.1	CRP	C-reactive protein precursor [Bos taurus]	7663570	10448084	4879056	1981750	1711410	1441068.625	-2.162831716	5.48E-03	2.08E-02
71	NP 001068871.2	CSF1R	macrophage colony-stimulating factor 1 receptor	232360	247654.83	217064.8906	1801591	2038380	2275167	3.132989468	2.48E-08	5.97E-07
72	NP 001071326.1	Ctps1	PREDICTED: CTP synthase 1 isoform X1 [Bos	4982050	1809115.9	8154986	527988.8	599800	671610.5	-3.054186805	3.94E-07	6.81E-06
73	NP 001029557.1	CTSH	pro-cathepsin H precursor [Bos taurus]	1906700	1845768	1967633.125	36944924	40367366	43789808	4.404039396	2.30E-12	1.39E-10
74	NP 001028787.1	CTSS	cathepsin S precursor [Bos taurus]	2245290	2531885	1958698.75	33101126	40544039	47986952	4.174515315	8.35E-07	1.29E-05
75	NP 001106645.1	CXCL12	PREDICTED: stromal cell-derived factor 1 isofo	163992	170234.48	157749.5469	0.001	0.001	0.001	-27.28905029	1.35E-06	1.96E-05
76	NP 001106643.1	CXCL9	C-X-C motif chemokine 9 precursor [Bos taurus]	0.001	0.001	0.001	411405.4	488704	566001.75	28.86438481	7.02E-10	2.36E-08
77	NP 776459.1	CYBA	cytochrome b-245 light chain [Bos taurus]	728873	654172.06	803574.125	7799199	10221200	12643212	3.809753708	1.69E-06	2.40E-05
78	NP 776460.1	CYBB	cytochrome b-245 heavy chain [Bos taurus]	110838	116638.18	105036.8984	3599923	3181050	2762183.25	4.842983542	9.97E-16	9.37E-14
79	XP 002697074.2	DCAF1	PREDICTED: protein VPRBP [Bos taurus]	193143	183670.16	202615.1094	337532.9	310041	282550	0.682792854	9.43E-03	3.22E-02
80	NP 001015545.1	DHX58	probable ATP-dependent RNA helicase DHX58	0.001	0.001	0.001	42101.71	62889.8	83677.86719	25.90632257	1.24E-04	8.73E-04
81	XP 024850712.1	DLG1	PREDICTED: disks large homolog 1 isoform X1	2188380	2219564.5	2157195.75	1032427	1063830	1095242.5	-1.040591297	1.10E-07	2.28E-06
82	XP 024841745.1	DMBT1	PREDICTED: deleted in malignant brain tumors	886828	770092.19	1003564.25	10198512	14518454	18838396	4.033089467	6.34E-05	4.95E-04
83	NP 001029603.1	DMTN	dematin [Bos taurus]	172164	176161.5	168165.9063	67937.58	74798.2	81658.84375	-1.202706246	5.23E-03	2.01E-02
84	XP 002698626.2	DOCK1	PREDICTED: dedicator of cytokinesis protein 1	311117	299246.28	322987.625	545514.1	628679	711843.1875	1.014865916	6.54E-07	1.04E-05
85	XP 024837188.1	DOCK2	PREDICTED: dedicator of cytokinesis protein 2	183051	199745.45	166356.2969	1561459	2027210	2492965.5	3.469179651	1.01E-18	1.45E-16
86	NP 001178461.2	DOCK8	TPA: dedicator of cytokinesis 8 [Bos taurus]	243442	281141.66	205742.375	930865	1291100	1651334.875	2.406950642	9.77E-10	3.24E-08
87	NP 001099123.1	Elane	neutrophil elastase precursor [Bos taurus]	147032	211740.03	82324.64844	2610617	3233160	3855708	4.458741343	5.14E-08	1.16E-06
88	XP 005214684.1	ELMO2	PREDICTED: engulfment and cell motility prote	93425.9	84409.922	102441.8047	676680.5	990008	1303335.875	3.405546282	2.11E-04	1.36E-03
89	NP 001069391.1	ENPP3	ectonucleotide pyrophosphatase/phosphodiestera	711382	731433.19	691329.9375	21857.08	23652.7	25448.30273	-4.910547484	2.51E-15	2.30E-13

90	NP 001007817.1	FADD	FAS-associated death domain protein [Bos taurus	1154540	1118602.5	1190471.625	1951905	2101600	2251303	0.864174169	9.59E-05	6.96E-04
91	NP 001033148.1	FBXO7	F-box only protein 7 [Bos taurus]	329660	331656.59	327663.9063	681202	779340	877477.3125	1.241272867	6.38E-05	4.95E-04
92	NP 776962.1	FCER1G	high affinity immunoglobulin epsilon receptor su	0.001	0.001	0.001	7491318	7873650	8255979	32.87438525	1.52E-06	2.16E-05
93	NP 776963.1	FCGR1A	high affinity immunoglobulin gamma Fc recepto	65100.6	34234.563	95966.65625	620273.7	614641	609009	3.23900166	1.42E-03	6.91E-03
94	NP 001136389.1	FGB	fibrinogen beta chain precursor [Bos taurus]	56568500	75997320	37139680	29893682	36486037	43078392	-0.632654458	1.77E-05	1.76E-04
95	NP 001157250.1	FN1	fibronectin precursor [Bos taurus]	5885269	6684608	5085930	18846242	21300816	23755390	1.855728435	3.27E-03	1.38E-02
96	NP 786995.2	FST	follistatin precursor [Bos taurus]	96963.8	77513.063	116414.5	0.001	0.001	0.001	-26.53094272	4.10E-05	3.54E-04
97	NP 776487.1	FTH1	ferritin heavy chain [Bos taurus]	412860	335383.75	490335.3438	6575705	6183150	5790602.5	3.904619089	4.24E-05	3.63E-04
98	NP 001029206.1	GAPDH	glyceraldehyde-3-phosphate dehydrogenase [Bos	89456188	88791392	90120984	2.13E+08	242518136	272403840	1.438839452	3.20E-03	1.35E-02
99	XP 010801604.1	GBP5	guanylate-binding protein 5 [Bos taurus]	0.001	0.001	0.001	358686	316671	274656.0625	28.23840956	4.29E-03	1.71E-02
100	NP 001193143.1	GGT1	gamma-glutamyltranspeptidase 1 [Bos taurus]	20276479	22106970	18445988	3049260	1784050	518849.0625	-3.506576754	3.13E-05	2.83E-04
101	NP 851364.1	GNAS	GNAS complex locus isoform GNASL [Bos taur	1996820	1383016.3	2610624.5	2954271	5307480	7660681	1.410321858	3.75E-04	2.26E-03
102	NP 001106785.1	GNG7	guanine nucleotide-binding protein G(I)/G(S)/G(	407179	456461.16	357896.625	824201	818633	813065.3125	1.007554109	2.49E-03	1.10E-02
103	XP 005225779.1	GPAM	PREDICTED: glycerol-3-phosphate acyltransfer	2997280	3297782.3	2696769.5	368826.8	253941	139054.9219	-3.561087913	2.82E-28	9.36E-26
104	NP 777241.1	GPLD1	phosphatidylinositol-glycan-specific phospholipa	353468	342631.66	364304.9375	86059.01	88579.2	91099.48438	-1.996540046	4.46E-05	3.77E-04
105	NP 001029802.1	GRB2	growth factor receptor-bound protein 2 isoform 1	1578630	1547580	1609677.375	5655711	6140720	6625728	1.959735472	6.36E-05	4.95E-04
106	NP 001030229.1	HCLS1	hematopoietic lineage cell-specific protein [Bos t	728138	822928.94	633346.125	6190041	6338880	6487712.5	3.121944275	2.47E-07	4.63E-06
107	NP 001012692.2	HLA-DMA	HLA class II histocompatibility antigen, DM alp	382151	414231.88	350069.6875	3197812	3746160	4294501	3.293197396	1.06E-02	3.55E-02
108	NP 001012693.2	HLA-DQA2	major histocompatibility complex, class II, DQ a	876181	815842.06	936520.1875	5276854	5518470	5760085	2.654967275	9.56E-04	4.95E-03
109	NP 001012695.1	HLA-DRA	Mamu class II histocompatibility antigen, DR alp	3077030	2822473.3	3331588.5	16532572	18504244	20475916	2.588245507	7.41E-05	5.63E-04
110	XP 024839853.1	HLA-DRB1	PREDICTED: HLA class II histocompatibility a	902864	921254.13	884473.125	2526992	2395410	2263820.5	1.40769086	2.74E-04	1.71E-03
111	NP 001014912.1	HMOX1	heme oxygenase 1 [Bos taurus]	119499	84506.398	154491.5	1890813	1960130	2029440.875	4.035877796	1.17E-06	1.72E-05
112	NP 001069184.1	HPS1	Hermansky-Pudlak syndrome 1 protein [Bos taur	68028.5	62221.645	73835.375	290689.5	288124	285559.3438	2.082479925	1.30E-03	6.37E-03
113	NP 001029784.1	HPX	hemopexin precursor [Bos taurus]	2564450	2415504.8	2713393.75	19856544	18405662	16954780	2.843428574	8.73E-04	4.59E-03
114	NP 001229275.1	HRAS	GTPase HRas isoform 1 [Bos taurus]	0.001	0.001	0.001	189303.1	224452	259601.0156	27.74183191	2.25E-05	2.15E-04
115	XP 002685173.1	ICOSLG	PREDICTED: ICOS ligand isoform X1 [Bos tau	686557	668622.06	704491.625	0.001	0.001	0.001	-29.35480404	5.50E-07	9.03E-06
116	NP 001095336.1	IDO1	indoleamine 2,3-dioxygenase 1 [Bos taurus]	288789	358491.91	219086.4844	11719216	16406202	21093188	5.828081045	1.02E-16	1.05E-14
117	XP 010800355.1	IFIH1	PREDICTED: interferon-induced helicase C dom	50687.7	46698.684	54676.78516	707014.6	846766	986516.625	4.062254657	6.24E-03	2.32E-02
118	XP 015316084.1	IFIT1	PREDICTED: interferon-induced protein with te	0.001	0.001	0.001	408102.8	465310	522518.0313	28.79361776	2.02E-14	1.67E-12
119	NP 001069166.1	IFIT5	interferon-induced protein with tetratricopeptide	198377	177666.2	219087.2188	597978.9	692461	786942.5625	1.803489062	8.51E-05	6.28E-04
120	NP 001098727.1	Ikzf1	DNA-binding protein Ikaros [Bos taurus]	117194	129183.65	105204.3516	209022	245075	281127.7813	1.064324123	1.76E-03	8.24E-03
121	XP 002692113.2	IL15RA	PREDICTED: interleukin-15 receptor subunit al	8481.28	0.001	16962.55664	349107.3	460364	571621.25	5.762349951	1.29E-03	6.36E-03
122	XP 015327398.1	IL1RAP	PREDICTED: interleukin-1 receptor accessory p	587970	603647.38	572293.0625	1384472	1428850	1473222.75	1.281037811	3.78E-03	1.55E-02
123	NP 001095352.1	INPP5D	phosphatidylinositol 3,4,5-trisphosphate 5-phosp	624988	618562.44	631414.375	2795976	3737900	4679820	2.580326406	1.44E-08	3.75E-07
124	NP 001178190.1	IRF1	interferon regulatory factor 1 [Bos taurus]	0.001	0.001	0.001	340245.4	350299	360353.1875	28.3840124	3.14E-07	5.64E-06
125	NP 001193091.1	IRF4	interferon regulatory factor 4 [Bos taurus]	83889.8	167779.64	0.001	200804.5	334301	467797.75	1.994580547	9.47E-06	1.03E-04
126	NP 001030542.1	IRF5	interferon regulatory factor 5 [Bos taurus]	100776	0.001	201551.9219	803313.5	1010390	1217465.125	3.325687975	1.23E-02	3.98E-02
127	NP 001077238.1	IRF8	interferon regulatory factor 8 [Bos taurus]	20499	38221.156	2776.765625	268255	311929	355602.3438	3.927593021	3.06E-08	7.23E-07
128	NP 776791.1	ISG15	ubiquitin-like protein ISG15 [Bos taurus]	378824	406916.09	350732.0625	1980867	2030500	2080139	2.422236699	1.16E-07	2.40E-06
129	XP 002696560.1	ISG20	PREDICTED: interferon-stimulated gene 20 kD	46647	78321.07	14972.98633	563439.6	610094	656748.125	3.709174556	1.26E-03	6.23E-03
130	NP 777173.1	ITGA4	integrin alpha-4 precursor [Bos taurus]	189751	147659.27	231842.8438	856695.1	985437	1114178.375	2.376655601	3.64E-05	3.21E-04
131	NP 001103451.1	ITGA6	integrin alpha-6 precursor [Bos taurus]	2908880	2709385	3108379.5	968871.8	890600	812327.9375	-1.707615144	4.43E-12	2.48E-10
132	NP 937864.2	ITGAL	integrin alpha-L precursor [Bos taurus]	88114.2	90228.664	85999.71875	2025606	2787660	3549716.75	4.983536814	6.08E-37	4.64E-34
133	NP 001035046.1	ITGAM	integrin alpha-M precursor [Bos taurus]	135423	150953.81	119891.5625	1844424	2435610	3026788.75	4.168739289	2.61E-19	4.33E-17
134	XP 024841064.1	ITGAX	PREDICTED: integrin alpha-X [Bison bison bis	74804	110603.35	39004.60938	2007776	2802170	3596563.5	5.227285442	1.16E-27	3.67E-25
135	NP 786975.1	ITGB2	integrin beta-2 precursor [Bos taurus]	251171	224308.33	278034.1563	8026062	10072000	12118001	5.325538557	2.50E-12	1.50E-10
136	NP 001098835.1	ITGB7	integrin beta-7 precursor [Bos taurus]	94073.9	43481.695	144666.0938	334409.4	388463	442516.8125	2.04591106	3.04E-10	1.14E-08
137	NP 001098834.1	JAM3	junctional adhesion molecule C precursor [Bos t	109696	95999.297	123392.375	10164.4	5082.2	0.001	-4.43191285	1.34E-02	4.27E-02
138	NP 001014405.1	KCNAB2	voltage-gated potassium channel subunit beta-2 i	167213	175974.14	158451.6563	1157405	1342810	1528206.25	3.005493717	6.42E-07	1.02E-05
139	NP 786968.2	KNG1	kininogen-2 isoform I precursor [Bos taurus]	0.001	0.001	0.001	1137709	1229230	1320755.75	30.19510945	4.24E-06	5.33E-05
140	XP 010803889.1	LAG3	PREDICTED: lymphocyte activation gene 3 prot	0.001	0.001	0.001	134560.1	169987	205414.5781	27.34085099	4.17E-05	3.59E-04
141	NP 001098448.1	LAT	linker for activation of T-cells family member 1	0.001	0.001	0.001	894515.6	1192120	1489721.875	30.15088131	5.46E-11	2.39E-09
142	XP 002691716.2	LCN2	PREDICTED: neutrophil gelatinase-associated li	1050040	1362162	737917.25	3367190	3186980	3006769	1.601745834	1.44E-03	7.00E-03
143	XP 005213731.1	LCP1	PREDICTED: plastin-2 isoform X1 [Bos taurus]	13614089	13843446	13384732	69645112	82371196	95097280	2.59703949	1.56E-10	6.26E-09
144	NP 001070312.1	LCP2	lymphocyte cytosolic protein 2 [Bos taurus]	520116	528430.44	511801.0938	1982457	2555450	3128444.5	2.296672826	1.34E-04	9.26E-04
145	NP 001039419.1	LGALS8	galectin-8 [Bos taurus]	607105	789731.19	424479.7188	273931.3	364789	455647.0938	-0.734884011	7.93E-03	2.81E-02
146	NP 001193833.1	LIMK1	LIM domain kinase 1 [Bos taurus]	308918	41593.148	576243.5625	786257.8	933744	1081230.75	1.595802314	1.37E-03	6.68E-03

147	NP 776358.1	LPO	PREDICTED: lactoperoxidase isoform X2 [Bos t	11416550	12627150	10205950	1496712	785207	73701.6875	-3.861909938	9.19E-32	4.12E-29
148	NP 001020253.1	LPXN	leupaxin [Bos taurus]	117337	84181.453	150492.7031	1763458	2068230	2373006.75	4.139667237	2.29E-09	6.98E-08
149	NP 851341.1	LTF	lactotransferrin precursor [Bos taurus]	55433350	69473904	41392796	1.46E+08	94993568	43530272	0.777075636	9.17E-04	4.77E-03
150	NP 001071297.1	LYZ	TPA: lysozyme F1 [Bos taurus]	153706	167481.41	139930.7031	0.001	0.001	0.001	-27.19559858	7.58E-06	8.56E-05
151	XP 002697882.2	MALT1	TPA: mucosa associated lymphoid tissue lymphc	0.001	0.001	0.001	157914.9	1854230	3550538.25	30.7881713	8.10E-04	4.32E-03
152	NP 001014947.1	MAPK13	mitogen-activated protein kinase 13 [Bos taurus]	1094030	1005235.6	1182818.875	198091.6	172011	145931.2188	-2.669074763	6.40E-03	2.36E-02
153	NP 001029951.1	MAPKAPK3	MAP kinase-activated protein kinase 3 [Bos taur	374348	351510.03	397185.9063	1001738	1120950	1240157.75	1.582268099	7.31E-03	2.62E-02
154	NP 001179001.1	MASP2	mannan-binding lectin serine protease 2 precurso	131342	133393.25	129290.9766	71974.61	70786.4	69598.09375	-0.891785706	2.26E-04	1.45E-03
155	XP 010817533.1	MMP25	TPA: matrix metalloproteinase 25 preproprotein-	0.001	0.001	0.001	404913.8	526475	648036.125	28.9717897	4.60E-05	3.85E-04
156	NP 777169.1	MMP9	matrix metalloproteinase-9 precursor [Bos taurus	14880.7	0.001	29761.38281	233449.8	299617	365783.9063	4.331605181	9.92E-05	7.18E-04
157	NP 001106769.1	MPO	myeloperoxidase precursor [Bos taurus]	72693.2	38973.215	106413.1719	303810.6	266040	228268.875	1.871749983	3.78E-07	6.60E-06
158	NP 001177227.1	MR1	major histocompatibility complex class I-related	0.001	0.001	0.001	189518.8	188564	187608.4531	27.49047703	2.36E-08	5.72E-07
159	XP 003586820.2	MRC1	PREDICTED: macrophage mannose receptor 1 i	305817	343220.94	268413.125	1020569	1195330	1370098.5	1.966671447	2.85E-06	3.78E-05
160	NP 001039942.1	MSN	moesin [Bos taurus]	10047900	9042350	11053363	39413496	43650486	47887476	2.119107869	9.91E-03	3.35E-02
161	NP 001179111.1	MUL1	mitochondrial ubiquitin ligase activator of NFKE	31199.4	34665.719	27733.1582	67611.49	69779.3	71947.04688	1.161279234	6.39E-03	2.36E-02
162	NP 001015528.1	MVK	mevalonate kinase [Bos taurus]	2513920	2211019.5	2816823	383279.8	408466	433652.7813	-2.621650655	8.68E-06	9.53E-05
163	NP 776365.1	MX1	interferon-induced GTP-binding protein Mx1 [Bo	2561860	2687722.8	2435997.5	6964380	8004140	9043902	1.643554807	2.24E-07	4.28E-06
164	NP 776366.1	MX2	interferon-induced GTP-binding protein Mx2 [Bo	689938	439767.88	940108.5625	1489638	2159930	2830222	1.646445653	1.80E-23	4.57E-21
165	NP 001179691.2	MYH9	TPA: myosin, heavy chain 9, non-muscle [Bos ta	19696900	15751047	23642844	38572988	43273734	47974480	1.135520803	5.33E-06	6.47E-05
166	NP 001179677.1	MYO1F	unconventional myosin-If [Bos taurus]	117500	133607.44	101392.7188	1438645	1650603	1862561.375	3.812259866	5.84E-13	4.05E-11
167	XP 005205671.1	MYO1G	PREDICTED: unconventional myosin-Ig [Bos ta	71395.5	66918.22	75872.74219	805194.1	977219	1149244.875	3.774777167	5.72E-14	4.44E-12
168	XP 005208558.1	MYO9B	PREDICTED: unconventional myosin-IXb isofo	63924.5	74476.117	53372.96094	542200.1	616656	691112.75	3.27002508	1.04E-11	5.33E-10
169	NP 776544.1	NCF1	neutrophil cytosol factor 1 [Bos taurus]	470668	682023.75	259312.3438	2997647	4355580	5713521.5	3.21008404	6.69E-12	3.56E-10
170	NP 001039448.1	NCF4	neutrophil cytosol factor 4 [Bos taurus]	41174.9	69480.195	12869.53125	1068843	1625140	2181431.5	5.302654391	9.95E-10	3.28E-08
171	XP 010803130.1	NCKAP1L	PREDICTED: nck-associated protein 1-like isofo	117256	123814.29	110698.2891	1353592	1824760	2295920.5	3.95996876	2.72E-11	1.30E-09
172	XP 005219255.1	NECTIN2	PREDICTED: nectin-2 isoform X2 [Bos taurus]	386901	366037.28	407764.7813	701812.6	734252	766691.0625	0.92431055	2.10E-03	9.55E-03
173	XP 024848569.1	NFAM1	PREDICTED: NFAT activation molecule 1 [Bos	0.539444	0.001	1.078887939	318029.6	508943	699856.5	19.84670843	5.46E-05	4.41E-04
174	NP 001160087.1	NFATC1	nuclear factor of activated T-cells, cytoplasmic 1	143000	159704.56	126295.9453	297548.9	267100	236651.7656	0.901364331	8.17E-06	9.11E-05
175	XP 002692403.3	NFATC2	PREDICTED: nuclear factor of activated T-cells,	99712.5	104296.15	95128.76563	591349.1	621545	651740.9375	2.640013031	1.56E-04	1.06E-03
176	NP 001095571.1	Nfkb2	nuclear factor NF-kappa-B p100 subunit [Bos tau	505469	476666.88	534270.4375	3028098	4059630	5091153	3.005653282	5.10E-07	8.48E-06
177	XP 005218862.1	NLRC5	PREDICTED: protein NLRC5 isoform X1 [Bos t	97261.1	194522.17	0.001	278136.4	337034	395932.5	1.792960707	5.08E-04	2.92E-03
178	NP 001095689.1	NLRP3	NACHT, LRR and PYD domains-containing pro	0.001	0.001	0.001	157629.6	175751	193871.5156	27.38895513	9.91E-06	1.07E-04
179	NP 001095705.1	NLRX1	NLR family member X1 [Bos taurus]	313837	320526.91	307146.5313	1267044	1465160	1663272.75	2.222970496	1.50E-05	1.53E-04
180	NP 001032713.1	NUTF2	PREDICTED: nuclear transport factor 2 isoform	6934643	5976060	7893226	11304004	11586400	11868707	0.740535165	4.56E-03	1.79E-02
181	NP 001035696.1	OAS1	2'-5'-oligoadenylate synthase 1 [Bos taurus]	136506	178571.72	94440.00781	1648349	1986140	2323931	3.862932043	9.73E-09	2.61E-07
182	NP 001075128.1	OASL	2'-5'-oligoadenylate synthase-like protein [Bos ta	0.001	0.001	0.001	161501.4	196582	231662.625	27.55055606	2.99E-05	2.72E-04
183	NP 001093798.1	OTULIN	ubiquitin thioesterase otulin [Bos taurus]	356723	370854.56	342591.1563	997617.3	1066900	1136181.625	1.580548689	1.69E-05	1.69E-04
184	NP 001070296.1	PARP9	poly [ADP-ribose] polymerase 9 [Bos taurus]	256929	264535.97	249322.625	505746.7	510598	515449	0.990816713	2.13E-05	2.07E-04
185	NP 776568.1	PIGR	polymeric immunoglobulin receptor precursor [B	14592607	19076220	10108994	8948532	6422040	3895546.5	-1.184134203	1.52E-07	3.05E-06
186	XP 002698457.4	PIK3AP1	PREDICTED: phosphoinositide 3-kinase adapter	1182450	1214659.9	1150239.625	2947001	3243010	3539026.5	1.455555608	5.31E-03	2.03E-02
187	XP 005205479.1	PIK3CG	PREDICTED: phosphatidylinositol 4,5-bisphosp	77597	72123.758	83070.17969	415096.1	555650	696203.6875	2.840103869	6.74E-04	3.73E-03
188	XP 024834660.1	PLCG2	PREDICTED: 1-phosphatidylinositol 4,5-bispho	236201	199184.06	273218.5	699158.1	928756	1158354.625	1.975283796	1.75E-05	1.74E-04
189	XP 002687006.4	PODXL	PREDICTED: podocalyxin [Bos taurus]	1177260	1109217.3	1245312	359762.6	317407	275051.5625	-1.891030621	1.00E-03	5.15E-03
190	XP 005205627.1	PPIA	peptidyl-prolyl cis-trans isomerase A [Sus scrofa	130149696	119438176	140861216	52447300	47963708	43480116	-1.440156841	7.52E-06	8.50E-05
191	XP 002688937.1	PRAM1	PREDICTED: PML-RARA-regulated adapter mo	0.001	0.001	0.001	77914.21	112773	147631.2031	26.74884396	3.97E-03	1.62E-02
192	NP 001033253.1	PRCP	lysosomal Pro-X carboxypeptidase precursor [Bo	400840	307031.91	494647.8125	1071434	1106670	1141911.875	1.465129426	1.03E-03	5.25E-03
193	NP 001193562.1	PRG4	proteoglycan 4 precursor [Bos taurus]	302696	435575.72	169817.1563	2310334	1183670	57006.94922	1.967324402	1.44E-03	6.99E-03
194	NP 777012.1	PRKCB	protein kinase C beta type [Bos taurus]	823699	723361	924037.125	2609023	2962758	3316492.75	1.846751576	3.59E-10	1.31E-08
195	XP 024838248.1	PRKCD	TPA: protein kinase C delta type [Bos taurus]	234486	277065.03	191907.6406	967252.1	1169430	1371604.625	2.318229073	1.68E-03	7.94E-03
196	XP 002689189.2	PRTN3	PREDICTED: myeloblastin [Bos taurus]	114751	205566.2	23935.13477	791686.3	872838	953989.5625	2.927209858	5.26E-04	3.01E-03
197	NP 001029212.1	PSMB10	proteasome subunit beta type-10 precursor [Bos t	582409	552148.13	612669.875	1781813	1901670	2021519.75	1.707160463	6.27E-05	4.90E-04
198	NP 001029560.1	PSMB9	proteasome subunit beta type-9 precursor [Bos ta	1073370	891350.25	1255386.75	3820173	3956160	4092145.5	1.881954495	4.72E-05	3.91E-04
199	NP 001019680.2	PSME1	proteasome activator complex subunit 1 [Bos tau	6230324	5618642.5	6842005.5	27151584	33704844	40258104	2.435576852	4.00E-03	1.63E-02
200	NP 776870.1	PTGS2	prostaglandin G/H synthase 2 precursor [Bos tau	0.001	0.001	0.001	230891.5	317620	404349.125	28.24272746	6.89E-05	5.27E-04
201	NP 001179432.2	PTPN22	tyrosine-protein phosphatase non-receptor type 2	0.001	0.001	0.001	70536.85	89763.6	108990.2734	26.4196268	4.73E-05	3.91E-04
202	XP 005207187.1	PTPN6	PREDICTED: tyrosine-protein phosphatase non-	3453119	3765319.5	3140918.5	10086658	11882520	13678382	1.782868872	7.83E-04	4.23E-03
203	NP 001193452.1	PTPRC	receptor-type tyrosine-protein phosphatase C pre	394909	474976.97	314840.8125	1472627	1487320	1502009.375	1.913122143	9.01E-19	1.32E-16

204	NP 001192460.2	PTPRE	PREDICTED: receptor-type tyrosine-protein pho	61327.1	60622.684	62031.59766	453524.3	555595	657665.5	3.17943618	6.04E-18	7.54E-16
205	XP 024831686.1	PTPRJ	PREDICTED: LOW QUALITY PROTEIN: rece	61196.6	31167.184	91225.98438	199260	281149	363038.3438	2.199812322	1.37E-06	1.97E-05
206	NP 777155.1	PYCARD	apoptosis-associated speck-like protein containin	1277787	1241461.1	1314112.875	20200430	24208596	28216762	4.243800139	1.26E-05	1.32E-04
207	NP 786986.1	RAC2	ras-related C3 botulinum toxin substrate 2 precur	1348820	1465153	1232482.25	7148171	8373700	9599232	2.634169236	3.04E-04	1.87E-03
208	NP 001070375.1	RB1	retinoblastoma-associated protein [Bos taurus]	18233.8	17255.549	19212.14453	77988.95	91785.9	105582.7813	2.331654484	9.87E-05	7.15E-04
209	NP 001192963.1	RBM47	RNA-binding protein 47 [Bos taurus]	4230091	4661189	3798993	1346037	1595709	1845381.375	-1.40649112	1.03E-02	3.46E-02
210	NP 001035565.2	RBP4	retinol-binding protein 4 precursor [Bos taurus]	1980950	2512994.8	1448907.75	599037.9	585480	571922.25	-1.758501176	7.89E-04	4.24E-03
211	XP 005211338.1	RIPK3	PREDICTED: receptor-interacting serine/threoni	133696	118059.58	149333.2188	422578.4	492316	562053.75	1.88062558	1.54E-03	7.43E-03
212	NP 001091634.1	RNASEL	2-5A-dependent ribonuclease [Bos taurus]	39733	29152.959	50313.00781	451698.3	675547	899394.75	4.087646271	1.59E-08	4.01E-07
213	NP 001068644.1	RPA1	replication protein A 70 kDa DNA-binding subu	841559	866531.69	816586.6875	1624784	1781250	1937722.125	1.081755163	1.55E-03	7.45E-03
214	XP 005209604.1	RPS14	PREDICTED: 40S ribosomal protein S14 isofor	10203900	10124383	10283362	4720590	4448104	4175618.5	-1.197855649	4.54E-03	1.79E-02
215	NP 777076.1	S100A12	protein S100-A12 [Bos taurus]	810297	1268350.1	352243.6875	16717773	15269476	13821179	4.236056056	4.76E-15	4.21E-13
216	NP 001107197.1	S100A8	protein S100-A8 [Bos taurus]	958208	1424265.4	492150.2813	7080975	6682904	6284833.5	2.802064559	3.16E-10	1.18E-08
217	NP 001039793.1	S100A9	protein S100-A9 [Bos taurus]	1179790	1480030.9	879543	18512716	22305284	26097852	4.240786023	7.20E-13	4.85E-11
218	NP 001029727.1	S100B	protein S100-B [Bos taurus]	64168440	65725096	62611784	11767650	10104790	8441930	-2.666824571	5.10E-03	1.97E-02
219	NP 001192710.1	SAMD9	sterile alpha motif domain-containing protein 9 [	1822970	200995.19	3444940	624757.7	815937	1007116.563	-1.159759734	5.04E-09	1.43E-07
220	XP 024856386.1	SAMHD1	PREDICTED: deoxynucleoside triphosphate trip	800837	821316.75	780356.625	8147168	9743470	11339763	3.604855055	7.01E-19	1.09E-16
221	NP 001030481.1	SAMSN1	SAM domain-containing protein SAMSN-1 [Bos	35599	41734.012	29463.92383	459710.4	520491	581272.0625	3.86996593	4.87E-18	6.18E-16
222	NP 001095988.1	SARM1	sterile alpha and TIR motif-containing protein 1	2107540	2415309.8	1799777.375	7356273	8005430	8654591	1.925417469	5.77E-05	4.59E-04
223	XP 010819700.1	SASH3	PREDICTED: SAM and SH3 domain-containing	259827	384155.63	135498.0313	484386.1	588703	693019.875	1.179989208	3.34E-05	2.99E-04
224	NP 001095510.1	Satb1	DNA-binding protein SATB1 [Bos taurus]	0.001	0.001	0.001	114947.7	167836	220724.9688	27.32247879	2.25E-10	8.57E-09
225	NP 001029611.1	SBDS	ribosome maturation protein SBDS [Bos taurus]	1397930	1429858.9	1366007.5	2270468	2300130	2329793.5	0.718421402	5.00E-03	1.94E-02
226	NP 001071379.1	SBNO2	protein strawberry notch homolog 2 [Bos taurus]	212262	332998	91526.33594	858384.5	1090180	1321980.375	2.360649726	1.15E-02	3.78E-02
227	NP 776602.1	SCIN	adseverin [Bos taurus]	2674740	3044262.5	2305222	48381656	54254976	60128296	4.342284086	1.51E-14	1.29E-12
228	NP 001035594.1	SEC61A1	protein transport protein Sec61 subunit alpha iso	5300959	5522598	5079320	975387.6	887637	799887.125	-2.578211265	1.18E-02	3.86E-02
229	NP 001068760.1	SEC61B	protein transport protein Sec61 subunit beta [Bos	8796677	9140294	8453060	2785268	3017450	3249630.25	-1.543629013	1.36E-02	4.31E-02
230	NP 001035676.1	SEC61G	PREDICTED: protein transport protein Sec61 su	7839900	8696076	6983733	3181715	3432490	3683262	-1.191580708	2.03E-03	9.28E-03
231	NP 001068908.1	SEMA4A	semaphorin-4A precursor [Bos taurus]	282606	479620.97	85591.29688	434569.7	479371	524172.2188	0.762349961	1.22E-02	3.96E-02
232	NP 001193805.1	SENP1	sentrin-specific protease 1 [Bos taurus]	0.001	0.001	0.001	221886.6	821450	1421014.375	29.6135981	7.37E-04	4.02E-03
233	NP 001091522.1	SGPL1	sphingosine-1-phosphate lyase 1 [Bos taurus]	138587	144903.09	132271.125	503510.5	780017	1056523.125	2.492712643	6.64E-03	2.43E-02
234	NP 001075912.1	SIGIRR	PREDICTED: single Ig IL-1-related receptor iso	246344	253558.89	239128.2188	601790.3	1070570	1539350.375	2.119634939	1.06E-03	5.38E-03
235	NP 001033303.1	SKAP2	src kinase-associated phosphoprotein 2 [Bos taur	68685.5	91782.508	45588.46094	1243908	1514060	1784207.25	4.462271591	1.39E-07	2.81E-06
236	NP 001070320.1	SLC26A6	solute carrier family 26 member 6 [Bos taurus]	82181.1	73106.102	91256.03906	157251.3	168739	180227.5938	1.037917752	6.38E-03	2.36E-02
237	NP 963285.2	SOD2	superoxide dismutase [Mn], mitochondrial precu	29739996	33656528	25823464	1.31E+08	112729016	94666464	1.922382549	4.29E-07	7.36E-06
238	XP 002696250.4	SPHK1	PREDICTED: LOW QUALITY PROTEIN: sphi	0.001	0.001	0.001	64632.25	78700.1	92767.95313	26.22986218	3.31E-05	2.97E-04
239	NP 001096572.1	Spn	leukosialin [Bos taurus]	23809.7	22881.449	24737.93164	343178.6	449464	555749.6875	4.23858494	2.02E-03	9.24E-03
240	NP 001020497.1	SRGN	serglycin precursor [Bos taurus]	137242	109258.5	165225.0156	258956.1	337549	416142.2813	1.298377188	2.11E-03	9.57E-03
241	NP 001094701.1	Srsf4	serine/arginine-rich splicing factor 4 [Bos taurus]	531922	551630.13	512213.1563	477497.8	238749	0.001	-1.155719806	1.80E-04	1.20E-03
242	XP 024835133.1	SSC5D	PREDICTED: soluble scavenger receptor cystein	310990	234503.75	387475.8125	0.001	0.001	0.001	-28.21229227	2.59E-09	7.79E-08
243	XP 010814530.1	STAT3	PREDICTED: signal transducer and activator of	863966	930333.88	797598.75	2012503	2093350	2174201.75	1.276767844	4.40E-03	1.75E-02
244	NP 001179556.1	STK10	serine/threonine-protein kinase 10 [Bos taurus]	315765	317485.41	314044.5313	1694707	2031028	2367349	2.68528705	1.19E-14	1.03E-12
245	NP 001015602.1	STK4	serine/threonine-protein kinase 4 [Bos taurus]	399466	323671.25	475261.4063	2361367	2829250	3297142.5	2.824275867	1.48E-08	3.84E-07
246	XP 010805126.1	STXBP2	PREDICTED: syntaxin-binding protein 2 isofor	721050	762011.81	680088.4375	1623115	1897730	2172354.25	1.396105683	3.37E-03	1.41E-02
247	NP 001039350.1	TAPBP	tapasin precursor [Bos taurus]	2748150	2516757.8	2979549.75	8959279	8511820	8064366.5	1.631005889	3.53E-03	1.47E-02
248	NP 001071353.1	TCF12	transcription factor 12 [Bos taurus]	121052	132300.03	109804.5469	193328.6	193503	193677.2969	0.67672645	1.29E-02	4.14E-02
249	NP 001106723.1	TGFB2	transforming growth factor beta-2 precursor [Bos	330875	389194.81	272554.2188	111536.1	55768	0.001	-2.568774896	2.30E-03	1.04E-02
250	NP 001029900.1	THBS4	thrombospondin-4 precursor [Bos taurus]	169517	248197.17	90836	95611.16	58397.5	21183.74609	-1.537449902	1.94E-04	1.27E-03
251	NP 001091585.1	THEMIS	protein THEMIS [Bos taurus]	0.001	0.001	0.001	156899.7	205884	254868.4063	27.61725666	4.21E-03	1.69E-02
252	XP 002685734.1	THEMIS2	PREDICTED: protein THEMIS2 [Bos taurus]	89723.1	86612.125	92834.09375	1357039	1783950	2210859	4.31345133	3.21E-09	9.37E-08
253	XP 015315453.1	TINAGL1	PREDICTED: tubulointerstitial nephritis antigen	5484100	4153137.5	6815069	3325794	2880229	2434664	-0.929071927	4.54E-04	2.66E-03
254	XP 005228545.1	TLR8	PREDICTED: toll-like receptor 8 isoform X1 [B	0.001	0.001	0.001	281317.6	330353	379388.3438	28.29943316	1.01E-09	3.31E-08
255	NP 001098966.1	TMED7	transmembrane emp24 domain-containing protei	10636341	11803427	9469255	2481205	2086810	1692408	-2.349632507	3.10E-03	1.32E-02
256	NP 001068812.1	TMEM102	transmembrane protein 102 [Bos taurus]	370606	319535.66	421677.1875	20869.52	25635	30400.45703	-3.853700933	5.58E-07	9.14E-06
257	NP 001039822.1	TMEM173	stimulator of interferon genes protein [Bos taurus	785000	1205414.8	364585.0938	2920248	3005960	3091673	1.937061468	1.24E-05	1.30E-04
258	NP 001179099.1	Tnfaip3	tumor necrosis factor alpha-induced protein 3 [B	22424.8	44849.688	0.001	382778.7	430221	477663.7188	4.26190948	9.55E-03	3.25E-02
259	NP 001077180.1	TNFAIP8	PREDICTED: tumor necrosis factor alpha-induc	560219	432421.13	688017	2860181	3538080	4215980.5	2.658903981	8.04E-08	1.72E-06
260	XP 024833579.1	TNIP3	PREDICTED: TNFAIP3-interacting protein 3 [B	0.001	0.001	0.001	154758.2	564089	973420.0625	29.07134778	7.50E-04	4.08E-03

261	NP 001035050.1	TOLLIP	toll-interacting protein [Bos taurus]	2365280	2332553.8	2398001.5	1703126	1536680	1370242.875	-0.622190585	1.02E-03	5.24E-03
262	NP 001014908.1	TRAFD1	TRAF-type zinc finger domain-containing protei	101506	81699.336	121313.1719	345538.8	398972	452405.0313	1.974719862	2.59E-05	2.41E-04
263	XP 002697385.1	TRIM26	PREDICTED: tripartite motif-containing protein	449943	365115.13	534770.3125	938711.8	900631	862549.9375	1.001194322	6.05E-03	2.27E-02
264	NP 001040014.1	TUBB	tubulin, beta 5, partial [Mus musculus]	28271883	26252390	30291376	57005148	61177366	65349584	1.113630018	1.57E-09	4.96E-08
265	NP 001069557.1	TWSG1	twisted gastrulation protein homolog 1 precursor	183483	158219.59	208745.4531	402098.1	394344	386589.6563	1.103810484	1.22E-02	3.95E-02
266	NP 777052.1	TYROBP	TYRO protein tyrosine kinase-binding protein pr	14461.6	28923.279	0.001	422048.5	518296	614542.5625	5.163473637	9.13E-05	6.68E-04
267	XP 010807164.1	UACA	PREDICTED: uveal autoantigen with coiled-coil	324023	283002.56	365044.25	252777.1	206592	160406.2344	-0.649314568	8.86E-07	1.35E-05
268	NP 001039961.1	ube2d2	ubiquitin-conjugating enzyme E2 D2 isoform 1 [	6702851	7289983	6115719	11119135	13814600	16510014	1.043345245	4.23E-05	3.62E-04
269	NP 001068603.1	UBE2D3	ubiquitin-conjugating enzyme E2 D3 [Bos taurus	6702851	7289983	6115719	11119135	13814600	16510014	1.043345245	4.23E-05	3.62E-04
270	NP 001179012.1	UNC13D	protein unc-13 homolog D [Bos taurus]	46792.2	0.001	93584.45313	419245.6	625531	831816.25	3.740740765	3.79E-11	1.70E-09
271	NP 001071542.1	VAV1	proto-oncogene vav [Bos taurus]	229037	272977.09	185097.5781	512705.9	715092	917478.6875	1.642547194	2.43E-08	5.87E-07
272	NP 001019727.2	VNN1	pantetheinase precursor [Bos taurus]	747407	735287.5	759526.0625	135478.4	160629	185780.125	-2.218159916	1.21E-03	6.04E-03
273	NP 001039994.1	VSIG4	V-set and immunoglobulin domain-containing pr	42478	50241.574	34714.35156	462055.6	483924	505791.625	3.509992809	2.44E-05	2.30E-04
274	XP 024844398.1	WAS	PREDICTED: wiskott-Aldrich syndrome protein	581078	891403.88	270752.1563	3238393	3989440	4740488	2.779382634	1.97E-10	7.62E-09
275	NP 001074980.1	WASF2	Wiskott-Aldrich syndrome protein family membe	912687	907295	918078.5625	2718601	3105390	3492187.5	1.766583886	6.28E-03	2.33E-02
276	XP 003586054.1	ZC3HAV1	PREDICTED: zinc finger CCCH-type antiviral p	519024	543269.88	494777.5313	1127369	1489690	1852015.5	1.521141109	1.81E-05	1.79E-04

DEPs Screen (13)

NO.	ID	Symbol	Description	Con1	Con2	Con3	CM1	CM2	CM3	log2(fc)	PValue	FDR
1	XP 005220161.3	ABR	PREDICTED: active breakpoint cluster region-re	279748	235274.14	324221.125	1974531	2551850	3129168.5	3.189344998	4.39862E-13	3.1E-11
2	XP 005213930.2	ACOD1	PREDICTED: cis-aconitate decarboxylase [Bos i	0.001	0.001	0.001	123591	153203	182815	27.19086926	2.50E-10	9.43E-09
3	NP 001039444.1	C6	complement component C6 precursor [Bos tauru	2772178	3473222.5	2071133.5	1386062	1351210	1316352.375	-1.036770165	2.19E-06	3.03E-05
4	NP 788811.1	CASP13	caspase-4 [Bos taurus]	192519	189969.3	195068.0469	1254216	1444160	1634098.625	2.907157615	6.86E-03	2.49E-02
5	NP 776962.1	FCER1G	high affinity immunoglobulin epsilon receptor su	0.001	0.001	0.001	7491318	7873650	8255979	32.87438525	1.51648E-06	2.16E-05
6	NP 776963.1	FCGR1A	high affinity immunoglobulin gamma Fc recepto	65100.6	34234.563	95966.65625	620273.7	614641	609009	3.23900166	1.42E-03	6.91E-03
7	XP 015316084.1	IFIT1	PREDICTED: interferon-induced protein with te	0.001	0.001	0.001	408102.8	465310	522518.0313	28.79361776	2.02E-14	1.67E-12
8	NP 001179111.1	MUL1	mitochondrial ubiquitin ligase activator of NFKE	31199.4	34665.719	27733.1582	67611.49	69779.3	71947.04688	1.161279234	6.39E-03	2.36E-02
9	NP 001095689.1	NLRP3	NACHT, LRR and PYD domains-containing pro	0.001	0.001	0.001	157629.6	175751	193871.5156	27.38895513	9.91E-06	1.07E-04
10	NP 001095705.1	NLRX1	NLR family member X1 [Bos taurus]	313837	320526.91	307146.5313	1267044	1465160	1663272.75	2.222970496	1.50E-05	1.53E-04
11	NP 776870.1	PTGS2	prostaglandin G/H synthase 2 precursor [Bos tau	0.001	0.001	0.001	230891.5	317620	404349.125	28.24272746	6.89E-05	5.27E-04
12	NP 777155.1	PYCARD	apoptosis-associated speck-like protein containin	1277787	1241461.1	1314112.875	20200430	24208596	28216762	4.243800139	1.26E-05	1.32E-04
13	NP 001096572.1	Spn	leukosialin [Bos taurus]	23809.7	22881.449	24737.93164	343178.6	449464	555749.6875	4.23858494	2.02E-03	9.24E-03

Table S6 The three significant KEGG Pathways and 72 DEPs identified from DIA proteomics.

Pathway ID	KEGG A class	KEGG B class	Pathway	Down DEPs	Up DEPs	Con-vs-CM	All	Pvalue	Qvalue	Proteins
ko04621	Organismal Systems	Immune system	NOD-like receptor signaling pathway	7	114	121	305	1.48E-05	1.70E-04	NP_001007817.1(FADD);NP_001012688.1
ko05133	Human Diseases	Infectious diseases	Pertussis	32	21	53	127	9.47E-04	5.73E-03	NP_001014947.1(MAPK13);NP_00103504
ko00330	Organismal Systems	Immune system	C-type lectin receptor signaling pathway	5	71	76	223	0.040274	1.52E-01	NP_001014947.1(MAPK13);NP_00101967

NO.	ID	Symbol	Description	Con1	Con2	Con3	CM1	CM2	CM3	log2(fc)	PValue	FDR
1	XP 005207185.1	C1R	PREDICTED: complement C1r subcomp	599655	763367.9375	435941.5	1117390.9	972059	826726.6	0.696911	5.60E-03	2.12E-02
2	NP 001070018.1	C1S	complement C1s subcomponent precurso	401839	458540.6563	345136.875	878137.38	792337	706537.3	0.979498	8.67E-04	4.56E-03
3	NP 001159957.1	C4A	complement C4 precursor [Bos taurus]	5815220	7843474	3786956.5	2267494.5	2306230	2344975	-1.334295	2.60271E-16	2.64E-14
4	NP 776677.1	C4BPA	PREDICTED: C4b-binding protein alpha	3158580	3476494.75	2840674.75	1815048.9	1803960	1792869	-0.808111	1.97E-04	1.28E-03
5	NP 001070579.1	CARD9	caspase recruitment domain-containing pı	54560.7	66155.0625	42966.25781	628866.13	791944	955021.6	3.859465	4.34033E-07	7.41E-06
6	XP 002692967.2	CASP1	PREDICTED: caspase-1 isoform X1 [Bo	70847.2	74900.98438	66793.47656	297047	351947	406847.8	2.312576	6.92956E-09	1.92E-07
7	NP 788811.1	CASP13	caspase-4 [Bos taurus]	192519	189969.2969	195068.0469	1254215.8	1444160	1634099	2.907158	0.006855989	0.024883
8	XP 002698555.1	CASP7	PREDICTED: caspase-7 [Bos taurus]	265505	243534.6094	287475.7188	1426244	1631240	1836240	2.619159	3.52E-03	1.46E-02
9	NP 777250.1	CATHL1	cathelicidin-1 precursor [Bos taurus]	1800210	2264273.5	1336138.375	14698460	17646468	20594476	3.293145	3.04822E-07	5.5E-06
10	NP 777251.1	CATHL2	cathelicidin-2 precursor [Bos taurus]	1944620	2573909.25	1315336.875	31816760	36862738	41908716	4.244602	0.000283247	0.001761
11	NP 776426.1	CATHL3	cathelicidin-3 precursor [Bos taurus]	306429	489717.5	123140.7031	3971091.3	4634180	5297278	3.91869	0.000879829	0.00461
12	NP 776935.1	CATHL5	cathelicidin-5 precursor [Bos taurus]	489270	557303.9375	421236.25	705699.38	1249530	1793367	1.352685	0.001704725	0.008034
13	NP 777257.1	CATHL6	cathelicidin-6 precursor [Bos taurus]	286472	379113.2188	193830.2656	1580048.6	1527360	1474671	2.414575	0.001602901	0.007646
14	NP 777256.1	CATHL7	cathelicidin-7 precursor [Bos taurus]	322268	376598.2813	267938.1563	4049598.8	5012270	5974947	3.959131	0.00026148	0.001641
15	NP 776433.1	CD14	monocyte differentiation antigen CD14 pı	1831820	2078287.75	1585345.625	4873841.5	5512970	6152095	1.589553	4.16E-03	1.67E-02
16	NP 001069622.1	CFL2	cofilin-2 isoform 1 [Homo sapiens]	6150510	10565663	1735364.375	1743498.5	1510710	1277913	-2.025483	5.29E-03	2.02E-02
17	NP 001029651.1	CLEC6A	C-type lectin domain family 6 member A	0.001	0.001	0.001	99772.867	141272	182771.6	27.0739	8.76E-05	6.45E-04
18	NP 776459.1	CYBA	cytochrome b-245 light chain [Bos taurus	728873	654172.0625	803574.125	7799199	10221200	12643212	3.809754	1.69218E-06	2.4E-05
19	NP 776460.1	CYBB	cytochrome b-245 heavy chain [Bos tauru	110838	116638.1797	105036.8984	3599923.3	3181050	2762183	4.842984	9.96687E-16	9.37E-14
20	XP 005206906.1	DNM1L	PREDICTED: dynamin-1-like protein iso	1528480	1585003.625	1471962.75	2069759.9	2370170	2670580	0.632891	0.000772221	0.004178
21	NP 001007817.1	FADD	FAS-associated death domain protein [Bc	1154540	1118602.5	1190471.625	1951904.8	2101600	2251303	0.864174	9.58612E-05	0.000696
22	NP 776962.1	FCER1G	high affinity immunoglobulin epsilon rec	0.001	0.001	0.001	7491318	7873650	8255979	32.87439	1.52E-06	2.16E-05
23	XP 010801658.1	GBP2	PREDICTED: guanylate-binding protein	507773	542716.625	472828.9063	9495702	13359902	17224102	4.717582	1.09634E-18	1.55E-16
24	NP 001231158.1	GBP3	interferon-induced guanylate-binding pro	1070290	1096762.875	1043825.313	17418188	23324472	29230756	4.445767	9.94267E-07	1.49E-05
25	XP 002686315.2	GBP4	PREDICTED: guanylate-binding protein	125719	127273.1875	124164.4141	2914005.8	3778350	4642695	4.909483	3.62551E-08	8.42E-07
26	XP 010801604.1	GBP5	guanylate-binding protein 5 [Bos taurus]	0.001	0.001	0.001	358685.97	316671	274656.1	28.23841	0.004286145	0.017086
27	XP 002686313.3	GBP7	PREDICTED: guanylate-binding protein	413253	463721.9063	362783.9688	5269689.5	6714320	8158952	4.022144	3.30903E-05	0.000297
28	NP 001039625.1	GSDMD	gasdermin-D [Bos taurus]	330700	339010.8125	322389.9375	2414254.8	3307760	4201257	3.322257	0.001806896	0.008429
29	NP 001229275.1	HRAS	GTPase HRas isoform 1 [Bos taurus]	0.001	0.001	0.001	189303.08	224452	259601	27.74183	2.25E-05	2.15E-04
30	NP 001012688.1	HSP90AA1	PREDICTED: heat shock protein HSP 90	34696520	34459776	34933264	83807888	95039932	1.06E+08	1.453743	0.000409788	0.002437
31	NP 776516.1	IL18	interleukin-18 precursor [Bos taurus]	188264	307519.25	69007.89844	1591240.3	1686870	1782496	3.163521	0.000188412	0.001242
32	NP 001035645.1	IRAK1	interleukin-1 receptor-associated kinase 1	96620.7	103984.3828	89257.03906	27683.004	31538.3	35393.66	-1.615227	7.87E-03	2.79E-02
33	NP 001178190.1	IRF1	interferon regulatory factor 1 [Bos taurus	0.001	0.001	0.001	340245.38	350299	360353.2	28.38401	3.13655E-07	5.64E-06
34	NP 001077238.1	IRF8	interferon regulatory factor 8 [Bos taurus	20499	38221.15625	2776.765625	268254.97	311929	355602.3	3.927593	3.06E-08	7.23E-07
35	NP 001019677.1	IRF9	interferon regulatory factor 9 [Bos taurus	65156.1	44228.78125	86083.4375	827563.56	727376	627188.9	3.480729	0.003936481	0.016062
36	NP 001035046.1	ITGAM	integrin alpha-M precursor [Bos taurus]	135423	150953.8125	119891.5625	1844424.3	2435610	3026789	4.168739	2.61E-19	4.33E-17
37	NP 786975.1	ITGB2	integrin beta-2 precursor [Bos taurus]	251171	224308.3281	278034.1563	8026062	10072000	12118001	5.325539	2.50289E-12	1.5E-10
38	NP 777266.1	ITPR1	inositol 1,4,5-trisphosphate receptor type	2125580	2211752	2039405.5	4264922	3458660	2652396	0.702357	0.000572964	0.003252
39	NP 001068842.1	LSP1	lymphocyte-specific protein 1 [Bos taurus	3054650	3840713.75	2268587	16659470	20287651	23915832	2.731523	1.05E-06	1.57E-05
40	XP 002697882.2	MALT1	TPA: mucosa associated lymphoid tissue	0.001	0.001	0.001	157914.94	1854230	3550538	30.78817	8.10E-04	4.32E-03
41	NP 001092423.1	MAPK12	mitogen-activated protein kinase 12 [Bos	233527	205946.5156	261107.5469	0.001	0.001	0.001	-27.79901	1.6389E-12	1.02E-10
42	NP 001014947.1	MAPK13	mitogen-activated protein kinase 13 [Bos	1094030	1005235.563	1182818.875	198091.63	172011	145931.2	-2.669075	6.40E-03	2.36E-02
43	XP 010815011.1	NAIP	PREDICTED: baculoviral IAP repeat-cor	2235410	140049.3125	4330775	635490.06	865714	1095937	-1.368578	2.20274E-18	3E-16
44	NP 001160087.1	NFATC1	nuclear factor of activated T-cells, cytopl	143000	159704.5625	126295.9453	297548.91	267100	236651.8	0.901364	8.17E-06	9.11E-05
45	XP 002692403.3	NFATC2	PREDICTED: nuclear factor of activated	99712.5	104296.1484	95128.76563	591349.13	621545	651740.9	2.640013	1.56E-04	1.06E-03
46	NP 001069877.1	NFKB1	nuclear factor NF-kappa-B p105 subunit	1865870	2267410.5	1464336.375	3678343.5	3933713	4189083	1.076041	5.95E-05	4.71E-04

47	NP 001095571.1	Nfkb2	nuclear factor NF-kappa-B p100 subunit	505469	476666.875	534270.4375	3028097.8	4059630	5091153	3.005653	5.10E-07	8.48E-06
48	NP 001179252.2	NLRC4	NLR family CARD domain-containing pr	12394.9	0.001	24789.73828	1004573.4	1230830	1457079	6.63374	3.08544E-18	3.98E-16
49	XP 003587454.2	NLRP1	PREDICTED: NACHT, LRR and PYD d	0.001	0.001	0.001	50866.859	66500.9	82135.02	25.98687	5.93632E-05	0.00047
50	NP 001095689.1	NLRP3	NACHT, LRR and PYD domains-contair	0.001	0.001	0.001	157629.56	175751	193871.5	27.38896	9.91E-06	1.07E-04
51	NP 001095705.1	NLRX1	NLR family member X1 [Bos taurus]	313837	320526.9063	307146.5313	1267043.5	1465160	1663273	2.22297	1.50211E-05	0.000153
52	NP 001035696.1	OAS1	2'-5'-oligoadenylate synthase 1 [Bos tauru	136506	178571.7188	94440.00781	1648349.1	1986140	2323931	3.862932	9.733E-09	2.61E-07
53	NP 001070366.1	PAK1	serine/threonine-protein kinase PAK 1 [B	164755	156239.5625	173271.4219	632366.56	726381	820395.4	2.140401	7.68E-04	4.16E-03
54	NP 001192477.1	PIK3CD	phosphatidylinositol 4,5-bisphosphate 3-k	81146.1	77108.65625	85183.60156	394132.75	445639	497144.4	2.457281	1.29E-03	6.33E-03
55	NP 001178330.1	PLCB2	1-phosphatidylinositol-4,5-bisphosphate p	168012	171636.4219	164387.8281	196136.34	258549	320960.8	0.621871	3.43223E-11	1.58E-09
56	XP 024834660.1	PLCG2	PREDICTED: 1-phosphatidylinositol 4,5-	236201	199184.0625	273218.5	699158.13	928756	1158355	1.975284	1.75E-05	1.74E-04
57	XP 024838248.1	PRKCD	TPA: protein kinase C delta type [Bos tau	234486	277065.0313	191907.6406	967252.06	1169430	1371605	2.318229	1.68E-03	7.94E-03
58	NP 001179969.1	PSTPIP1	proline-serine-threonine phosphatase-inte	146253	154651.7969	137854.0781	774496.19	989068	1203640	2.757604	2.1878E-07	4.2E-06
59	NP 776870.1	PTGS2	prostaglandin G/H synthase 2 precursor [	0.001	0.001	0.001	230891.5	317620	404349.1	28.24273	6.89E-05	5.27E-04
60	NP 777155.1	PYCARD	apoptosis-associated speck-like protein co	1277787	1241461.125	1314112.875	20200430	24208596	28216762	4.2438	1.26E-05	1.32E-04
61	XP 002695213.2	RELB	PREDICTED: transcription factor RelB i	75851.3	46549.80859	105152.7891	557756.75	598651	639545.8	2.98047	8.97E-03	3.09E-02
62	XP 005211338.1	RIPK3	PREDICTED: receptor-interacting serine	133696	118059.5781	149333.2188	422578.38	492316	562053.8	1.880626	0.001543673	0.007425
63	NP 001091634.1	RNASEL	2-5A-dependent ribonuclease [Bos taurus	39733	29152.95898	50313.00781	451698.34	675547	899394.8	4.087646	1.58532E-08	4.01E-07
64	XP 005202627.2	STAT1	PREDICTED: signal transducer and activ	1463860	1434132.5	1493589.875	19975310	25182196	30389082	4.104554	8.61382E-18	1.06E-15
65	NP 001192618.1	STAT2	signal transducer and activator of transcri	215061	223570.5313	206552.1094	729380.31	846358	963336.5	1.976521	2.334E-07	4.42E-06
66	XP 005210492.1	SYK	PREDICTED: tyrosine-protein kinase SY	141941	122056.5938	161825.5469	1335928.4	1748610	2161285	3.622843	3.85557E-13	2.75E-11
67	NP 001179193.1	TANK	TRAF family member-associated NF-kap	121685	151956.2188	91414.14063	204742.45	228447	252150.9	0.908705	0.000324582	0.001978
68	NP 001039822.1	TMEM173	stimulator of interferon genes protein [Bo	785000	1205414.75	364585.0938	2920247.8	3005960	3091673	1.937061	1.24257E-05	0.00013
69	NP 001179099.1	Tnfaip3	tumor necrosis factor alpha-induced prote	22424.8	44849.6875	0.001	382778.69	430221	477663.7	4.261909	0.009554618	0.032507
70	XP 010799963.1	TRPM2	PREDICTED: transient receptor potential	134214	1.352110624	268425.7813	321977.75	363342	404705.8	1.436795	5.15625E-07	8.56E-06
71	NP 001019664.1	TRPV2	transient receptor potential cation channe	40423.2	0.001	80846.40625	762275.75	1061430	1360575	4.714678	3.7476E-08	8.68E-07
72	NP 001107236.1	TYK2	non-receptor tyrosine-protein kinase TYK	38718.1	8231.158203	69205.00781	389244.66	431415	473586.1	3.477997	0.00050087	0.002886

NOD-like receptor signaling pathway

NO.	ID	Symbol	Description	Con1	Con2	Con3	CM1	CM2	CM3	log2(fc)	PValue	FDR
1	NP 001070579.1	CARD9	caspase recruitment domain-containing pr	54560.7	66155.0625	42966.25781	628866.13	791944	955021.6	3.859465	4.34033E-07	7.41E-06
2	XP 002692967.2	CASP1	PREDICTED: caspase-1 isoform X1 [Bos	70847.2	74900.98438	66793.47656	297047	351947	406847.8	2.312576	6.92956E-09	1.92E-07
3	NP 788811.1	CASP13	caspase-4 [Bos taurus]	192519	189969.2969	195068.0469	1254215.8	1444160	1634099	2.907158	0.006855989	0.024883
4	NP 777250.1	CATHL1	cathelicidin-1 precursor [Bos taurus]	1800210	2264273.5	1336138.375	14698460	17646468	20594476	3.293145	3.04822E-07	5.5E-06
5	NP 777251.1	CATHL2	cathelicidin-2 precursor [Bos taurus]	1944620	2573909.25	1315336.875	31816760	36862738	41908716	4.244602	0.000283247	0.001761
6	NP 776426.1	CATHL3	cathelicidin-3 precursor [Bos taurus]	306429	489717.5	123140.7031	3971091.3	4634180	5297278	3.91869	0.000879829	0.00461
7	NP 776935.1	CATHL5	cathelicidin-5 precursor [Bos taurus]	489270	557303.9375	421236.25	705699.38	1249530	1793367	1.352685	0.001704725	0.008034
8	NP 777257.1	CATHL6	cathelicidin-6 precursor [Bos taurus]	286472	379113.2188	193830.2656	1580048.6	1527360	1474671	2.414575	0.001602901	0.007646
9	NP 777256.1	CATHL7	cathelicidin-7 precursor [Bos taurus]	322268	376598.2813	267938.1563	4049598.8	5012270	5974947	3.959131	0.00026148	0.001641
10	NP 776459.1	CYBA	cytochrome b-245 light chain [Bos taurus	728873	654172.0625	803574.125	7799199	10221200	12643212	3.809754	1.69218E-06	2.4E-05
11	NP 776460.1	CYBB	cytochrome b-245 heavy chain [Bos tauru	110838	116638.1797	105036.8984	3599923.3	3181050	2762183	4.842984	9.96687E-16	9.37E-14
12	XP 005206906.1	DNM1L	PREDICTED: dynamin-1-like protein iso	1528480	1585003.625	1471962.75	2069759.9	2370170	2670580	0.632891	0.000772221	0.004178
13	NP 001007817.1	FADD	FAS-associated death domain protein [Bo	1154540	1118602.5	1190471.625	1951904.8	2101600	2251303	0.864174	9.58612E-05	0.000696
14	XP 010801658.1	GBP2	PREDICTED: guanylate-binding protein	507773	542716.625	472828.9063	9495702	13359902	17224102	4.717582	1.09634E-18	1.55E-16
15	NP 001231158.1	GBP3	interferon-induced guanylate-binding pro	1070290	1096762.875	1043825.313	17418188	23324472	29230756	4.445767	9.94267E-07	1.49E-05
16	XP 002686315.2	GBP4	PREDICTED: guanylate-binding protein	125719	127273.1875	124164.4141	2914005.8	3778350	4642695	4.909483	3.62551E-08	8.42E-07
17	XP 010801604.1	GBP5	guanylate-binding protein 5 [Bos taurus]	0.001	0.001	0.001	358685.97	316671	274656.1	28.23841	0.004286145	0.017086
18	XP 002686313.3	GBP7	PREDICTED: guanylate-binding protein	413253	463721.9063	362783.9688	5269689.5	6714320	8158952	4.022144	3.30903E-05	0.000297
19	NP 001039625.1	GSDMD	gasdermin-D [Bos taurus]	330700	339010.8125	322389.9375	2414254.8	3307760	4201257	3.322257	0.001806896	0.008429
20	NP 001012688.1	HSP90AA1	PREDICTED: heat shock protein HSP 90	34696520	34459776	34933264	83807888	95039932	1.06E+08	1.453743	0.000409788	0.002437
21	NP 776516.1	IL18	interleukin-18 precursor [Bos taurus]	188264	307519.25	69007.89844	1591240.3	1686870	1782496	3.163521	0.000188412	0.001242
22	NP 001019677.1	IRF9	interferon regulatory factor 9 [Bos taurus	65156.1	44228.78125	86083.4375	827563.56	727376	627188.9	3.480729	0.003936481	0.016062
23	NP 777266.1	ITPR1	inositol 1,4,5-trisphosphate receptor type	2125580	2211752	2039405.5	4264922	3458660	2652396	0.702357	0.000572964	0.003252
24	NP 001092423.1	MAPK12	mitogen-activated protein kinase 12 [Bos	233527	205946.5156	261107.5469	0.001	0.001	0.001	-27.79901	1.6389E-12	1.02E-10

25	NP 001014947.1	MAPK13	mitogen-activated protein kinase 13 [Bos	1094030	1005235.563	1182818.875	198091.63	172011	145931.2	-2.669075	0.006398501	0.023645
26	XP 010815011.1	NAIP	PREDICTED: baculoviral IAP repeat-cor	2235410	140049.3125	4330775	635490.06	865714	1095937	-1.368578	2.20274E-18	3E-16
27	NP 001069877.1	NFKB1	nuclear factor NF-kappa-B p105 subunit		1865870	2267410.5	1464336.375	3678343.5	3933713	4189083	1.076041	5.95398E-05	0.000471
28	NP 001179252.2	NLRC4	NLR family CARD domain-containing pı	12394.9	0.001	24789.73828	1004573.4	1230830	1457079	6.63374	3.08544E-18	3.98E-16
29	XP 003587454.2	NLRP1	PREDICTED: NACHT, LRR and PYD d	0.001	0.001	0.001	50866.859	66500.9	82135.02	25.98687	5.93632E-05	0.00047
30	NP 001095689.1	NLRP3	NACHT, LRR and PYD domains-contair	0.001	0.001	0.001	157629.56	175751	193871.5	27.38896	9.91475E-06	0.000107
31	NP 001095705.1	NLRX1	NLR family member X1 [Bos taurus]	313837	320526.9063	307146.5313	1267043.5	1465160	1663273	2.22297	1.50211E-05	0.000153
32	NP 001035696.1	OAS1	2'-5'-oligoadenylate synthase 1 [Bos tauru	136506	178571.7188	94440.00781	1648349.1	1986140	2323931	3.862932	9.733E-09	2.61E-07
33	NP 001178330.1	PLCB2	1-phosphatidylinositol-4,5-bisphosphate ı	168012	171636.4219	164387.8281	196136.34	258549	320960.8	0.621871	3.43223E-11	1.58E-09
34	XP 024838248.1	PRKCD	TPA: protein kinase C delta type [Bos tau	234486	277065.0313	191907.6406	967252.06	1169430	1371605	2.318229	0.001679189	0.007936
35	NP 001179969.1	PSTPIP1	proline-serine-threonine phosphatase-inte	146253	154651.7969	137854.0781	774496.19	989068	1203640	2.757604	2.1878E-07	4.2E-06
36	NP 777155.1	PYCARD	apoptosis-associated speck-like protein cc	1277787	1241461.125	1314112.875	20200430	24208596	28216762	4.2438	1.25953E-05	0.000132
37	XP 005211338.1	RIPK3	PREDICTED: receptor-interacting serine	133696	118059.5781	149333.2188	422578.38	492316	562053.8	1.880626	0.001543673	0.007425
38	NP 001091634.1	RNASEL	2-5A-dependent ribonuclease [Bos taurus	39733	29152.95898	50313.00781	451698.34	675547	899394.8	4.087646	1.58532E-08	4.01E-07
39	XP 005202627.2	STAT1	PREDICTED: signal transducer and activ	1463860	1434132.5	1493589.875	19975310	25182196	30389082	4.104554	8.61382E-18	1.06E-15
40	NP 001192618.1	STAT2	signal transducer and activator of transcri	215061	223570.5313	206552.1094	729380.31	846358	963336.5	1.976521	2.334E-07	4.42E-06
41	NP 001179193.1	TANK	TRAF family member-associated NF-kap	121685	151956.2188	91414.14063	204742.45	228447	252150.9	0.908705	0.000324582	0.001978
42	NP 001039822.1	TMEM173	stimulator of interferon genes protein [Bo	785000	1205414.75	364585.0938	2920247.8	3005960	3091673	1.937061	1.24257E-05	0.00013
43	NP 001179099.1	Tnfaip3	tumor necrosis factor alpha-induced prote	22424.8	44849.6875	0.001	382778.69	430221	477663.7	4.261909	0.009554618	0.032507
44	XP 010799963.1	TRPM2	PREDICTED: transient receptor potential	134214	1.352110624	268425.7813	321977.75	363342	404705.8	1.436795	5.15625E-07	8.56E-06
45	NP 001019664.1	TRPV2	transient receptor potential cation channe	40423.2	0.001	80846.40625	762275.75	1061430	1360575	4.714678	3.7476E-08	8.68E-07
46	NP 001107236.1	TYK2	non-receptor tyrosine-protein kinase TYK	38718.1	8231.158203	69205.00781	389244.66	431415	473586.1	3.477997	0.00050087	0.002886

Pertussis

NO.	ID	Symbol	Description	Con1	Con2	Con3	CM1	CM2	CM3	log2(fc)	PValue	FDR
1	XP 005207185.1	C1R	PREDICTED: complement C1r subcomp	599655	763367.9375	435941.5	1117390.9	972059	826726.6	0.696911	5.60E-03	2.12E-02
2	NP 001070018.1	C1S	complement C1s subcomponent precurso	401839	458540.6563	345136.875	878137.38	792337	706537.3	0.979498	8.67E-04	4.56E-03
3	NP 001159957.1	C4A	complement C4 precursor [Bos taurus]	5815220	7843474	3786956.5	2267494.5	2306230	2344975	-1.334295	2.60271E-16	2.64E-14
4	NP 776677.1	C4BPA	PREDICTED: C4b-binding protein alpha	3158580	3476494.75	2840674.75	1815048.9	1803960	1792869	-0.808111	1.97E-04	1.28E-03
5	XP 002692967.2	CASP1	PREDICTED: caspase-1 isoform X1 [Bo	70847.2	74900.98438	66793.47656	297047	351947	406847.8	2.312576	6.92956E-09	1.92E-07
6	XP 002698555.1	CASP7	PREDICTED: caspase-7 [Bos taurus]	265505	243534.6094	287475.7188	1426244	1631240	1836240	2.619159	3.52E-03	1.46E-02
7	NP 776433.1	CD14	monocyte differentiation antigen CD14 pı	1831820	2078287.75	1585345.625	4873841.5	5512970	6152095	1.589553	4.16E-03	1.67E-02
8	NP 001069622.1	CFL2	cofilin-2 isoform 1 [Homo sapiens]	6150510	10565663	1735364.375	1743498.5	1510710	1277913	-2.025483	5.29E-03	2.02E-02
9	NP 001035645.1	IRAK1	interleukin-1 receptor-associated kinase 1	96620.7	103984.3828	89257.03906	27683.004	31538.3	35393.66	-1.615227	7.87E-03	2.79E-02
10	NP 001178190.1	IRF1	interferon regulatory factor 1 [Bos taurus	0.001	0.001	0.001	340245.38	350299	360353.2	28.38401	3.13655E-07	5.64E-06
11	NP 001077238.1	IRF8	interferon regulatory factor 8 [Bos taurus	20499	38221.15625	2776.765625	268254.97	311929	355602.3	3.927593	3.06E-08	7.23E-07
12	NP 001035046.1	ITGAM	integrin alpha-M precursor [Bos taurus]	135423	150953.8125	119891.5625	1844424.3	2435610	3026789	4.168739	2.61E-19	4.33E-17
13	NP 786975.1	ITGB2	integrin beta-2 precursor [Bos taurus]	251171	224308.3281	278034.1563	8026062	10072000	12118001	5.325539	2.50289E-12	1.5E-10
14	NP 001092423.1	MAPK12	mitogen-activated protein kinase 12 [Bos	233527	205946.5156	261107.5469	0.001	0.001	0.001	-27.79901	1.6389E-12	1.02E-10
15	NP 001014947.1	MAPK13	mitogen-activated protein kinase 13 [Bos	1094030	1005235.563	1182818.875	198091.63	172011	145931.2	-2.669075	6.40E-03	2.36E-02
16	NP 001069877.1	NFKB1	nuclear factor NF-kappa-B p105 subunit		1865870	2267410.5	1464336.375	3678343.5	3933713	4189083	1.076041	5.95E-05	4.71E-04
17	NP 001095689.1	NLRP3	NACHT, LRR and PYD domains-contair	0.001	0.001	0.001	157629.56	175751	193871.5	27.38896	9.91E-06	1.07E-04
18	NP 777155.1	PYCARD	apoptosis-associated speck-like protein cc	1277787	1241461.125	1314112.875	20200430	24208596	28216762	4.2438	1.26E-05	1.32E-04

C-type lectin receptor signaling pathway

NO.	ID	Symbol	Description	Con1	Con2	Con3	CM1	CM2	CM3	log2(fc)	PValue	FDR
1	NP 001070579.1	CARD9	caspase recruitment domain-containing pı	54560.7	66155.0625	42966.25781	628866.13	791944	955021.6	3.859465	4.34033E-07	7.41E-06
2	XP 002692967.2	CASP1	PREDICTED: caspase-1 isoform X1 [Bo	70847.2	74900.98438	66793.47656	297047	351947	406847.8	2.312576	6.92956E-09	1.92E-07
3	NP 001029651.1	CLEC6A	C-type lectin domain family 6 member A	0.001	0.001	0.001	99772.867	141272	182771.6	27.0739	8.76E-05	6.45E-04
4	NP 776962.1	FCER1G	high affinity immunoglobulin epsilon rec	0.001	0.001	0.001	7491318	7873650	8255979	32.87439	1.52E-06	2.16E-05
5	NP 001229275.1	HRAS	GTPase HRas isoform 1 [Bos taurus]	0.001	0.001	0.001	189303.08	224452	259601	27.74183	2.25E-05	2.15E-04
6	NP 001178190.1	IRF1	interferon regulatory factor 1 [Bos taurus	0.001	0.001	0.001	340245.38	350299	360353.2	28.38401	3.13655E-07	5.64E-06

7	NP 001019677.1	IRF9	interferon regulatory factor 9 [Bos taurus	65156.1	44228.78125	86083.4375	827563.56	727376	627188.9	3.480729	0.003936481	0.016062
8	NP 777266.1	ITPR1	inositol 1,4,5-trisphosphate receptor type	2125580	2211752	2039405.5	4264922	3458660	2652396	0.702357	0.000572964	0.003252
9	NP 001068842.1	LSP1	lymphocyte-specific protein 1 [Bos taurus	3054650	3840713.75	2268587	16659470	20287651	23915832	2.731523	1.05E-06	1.57E-05
10	XP 002697882.2	MALT1	TPA: mucosa associated lymphoid tissue	0.001	0.001	0.001	157914.94	1854230	3550538	30.78817	8.10E-04	4.32E-03
11	NP 001092423.1	MAPK12	mitogen-activated protein kinase 12 [Bos	233527	205946.5156	261107.5469	0.001	0.001	0.001	-27.79901	1.6389E-12	1.02E-10
12	NP 001014947.1	MAPK13	mitogen-activated protein kinase 13 [Bos	1094030	1005235.563	1182818.875	198091.63	172011	145931.2	-2.669075	0.006398501	0.023645
13	NP 001160087.1	NFATC1	nuclear factor of activated T-cells, cytopl	143000	159704.5625	126295.9453	297548.91	267100	236651.8	0.901364	8.17E-06	9.11E-05
14	XP 002692403.3	NFATC2	PREDICTED: nuclear factor of activated	99712.5	104296.1484	95128.76563	591349.13	621545	651740.9	2.640013	1.56E-04	1.06E-03
15	NP 001069877.1	NFKB1	nuclear factor NF-kappa-B p105 subunit	1865870	2267410.5	1464336.375	3678343.5	3933713	4189083	1.076041	5.95398E-05	0.000471
16	NP 001095571.1	Nfkb2	nuclear factor NF-kappa-B p100 subunit	505469	476666.875	534270.4375	3028097.8	4059630	5091153	3.005653	5.10E-07	8.48E-06
17	NP 001095689.1	NLRP3	NACHT, LRR and PYD domains-contair	0.001	0.001	0.001	157629.56	175751	193871.5	27.38896	9.91475E-06	0.000107
18	NP 001070366.1	PAK1	serine/threonine-protein kinase PAK 1 [B	164755	156239.5625	173271.4219	632366.56	726381	820395.4	2.140401	7.68E-04	4.16E-03
19	NP 001192477.1	PIK3CD	phosphatidylinositol 4,5-bisphosphate 3-k	81146.1	77108.65625	85183.60156	394132.75	445639	497144.4	2.457281	1.29E-03	6.33E-03
20	XP 024834660.1	PLCG2	PREDICTED: 1-phosphatidylinositol 4,5	236201	199184.0625	273218.5	699158.13	928756	1158355	1.975284	1.75E-05	1.74E-04
21	XP 024838248.1	PRKCD	TPA: protein kinase C delta type [Bos tau	234486	277065.0313	191907.6406	967252.06	1169430	1371605	2.318229	1.68E-03	7.94E-03
22	NP 776870.1	PTGS2	prostaglandin G/H synthase 2 precursor [	0.001	0.001	0.001	230891.5	317620	404349.1	28.24273	6.89E-05	5.27E-04
23	NP 777155.1	PYCARD	apoptosis-associated speck-like protein cc	1277787	1241461.125	1314112.875	20200430	24208596	28216762	4.2438	1.25953E-05	0.000132
24	XP 002695213.2	RELB	PREDICTED: transcription factor RelB i	75851.3	46549.80859	105152.7891	557756.75	598651	639545.8	2.98047	8.97E-03	3.09E-02
25	XP 005202627.2	STAT1	PREDICTED: signal transducer and activ	1463860	1434132.5	1493589.875	19975310	25182196	30389082	4.104554	8.61382E-18	1.06E-15
26	NP 001192618.1	STAT2	signal transducer and activator of transcri	215061	223570.5313	206552.1094	729380.31	846358	963336.5	1.976521	2.334E-07	4.42E-06
27	XP 005210492.1	SYK	PREDICTED: tyrosine-protein kinase SY	141941	122056.5938	161825.5469	1335928.4	1748610	2161285	3.622843	3.85557E-13	2.75E-11
